# Zero-PDG silicon photonic amplifier with high saturation power and low noise figure

**DOI:** 10.1038/s41467-026-74486-y

**Published:** 2026-06-30

**Authors:** Jan Lorenzen, Kai Wang, Muharrem Kilinc, Mikhail Pergament, Sonia M. Garcia-Blanco, Franz X. Kärtner, Neetesh Singh

**Affiliations:** 1 Center for Free-Electron Laser Science CFEL, Deutsches Elektronen-Synchrotron DESY, Hamburg, Germany; 2 Department of Physics, Universität Hamburg, Hamburg, Germany; 3 Integrated Optical Systems, MESA+ Institute for Nanotechnology, University of Twente, Enschede, The Netherlands

**Keywords:** Integrated optics, Solid-state lasers

## Abstract

High-power amplifiers are of great importance in many optical systems deployed in optical sensing, ranging, medical surgery and more. Likewise, high-gain, low-noise amplifiers with low polarization dependence are critical components of long-range optical communication systems. Integrated photonic solutions show great potential in challenging application fields thanks to their drastic reduction in size, weight and cost, but coming at the expense of low optical power due to reduced energy storage capacity in small devices. Recently, the large mode area (LMA) technology, which is known for dramatically increasing the energy storage capacity and saturation power of fiber amplifiers, has been brought to the chip-level, allowing for watt-level amplification directly from the chip. Here we demonstrate a silicon photonic LMA amplifier capable of both high-power amplification with output saturation powers > 115 mW and high small-signal gain up to 30 dB with a 3.6 dB noise figure on-chip, which we achieved by suppressing parasitic lasing with index-matching glue on the chip facets. Furthermore, we present a method to tune and completely nullify the polarization dependent gain (PDG). We believe that the PDG-tunability combined with the low-noise, high-gain and high-power amplification positions it as a potentially disruptive technology for next-generation integrated amplifiers across a range of applications.

## Abstract (web summary)

The authors present a silicon photonics large mode area (LMA) amplifier delivering both high-power amplification to the watt-level and high gain up to 30 dB with a low noise figure below 4 dB. Furthermore, the amplifier can be tuned to be completely polarization independent.

## Introduction

There are several key attributes that are highly desirable in optical amplifiers, including high net gain, high saturation power, low noise, and polarization insensitivity. High output power is essential for a wide range of applications, including optical sensing, detection and ranging, medical surgery, material processing, amplification of optical frequency combs, and mode-locked lasers^1–10^. In contrast, low noise and low polarization sensitivity are particularly critical for long-range optical telecommunications, where they directly impact signal integrity and average channel capacity^11^. While fiber-based amplifier systems exhibit many of these desirable characteristics, they tend to be bulky and are not well-suited for the growing demand for miniaturized and scalable platforms^12–14^. Additionally, their large form factor makes them unsuitable for challenging environments, such as deep space, where compact, lightweight solutions are required^15–18^. There is also increasing interest in expanding beyond the traditional C- and O-band telecom windows by leveraging the broad gain bandwidth available at 2 µm^10,19,20^. This interest is largely driven by the surging data demands of AI and high-throughput computing applications. Unlike fiber-based systems, chip-scale optical amplifiers offer the potential for multi-band amplification within a single integrated platform, significantly reducing system footprint while improving scalability. Recent advancements in semiconductor optical amplifiers (SOAs), particularly those hybridly or heterogeneously integrated onto silicon photonics platforms, have shown great promise. These devices benefit from electrical pumping, high optical gain, and improvements in fabrication yield and integration techniques^21–25^. However, their gain saturation power remains relatively low—typically limited to a few tens of milliwatts^22,23,26–29^—and this limitation is more pronounced in the 2 µm wavelength range^28^, where output powers are often significantly lower than in the C-band. In addition, achieving low-noise amplification with integrated SOAs remains challenging^27^, and their performance is often highly sensitive to the state of polarization of the input signal, leading to polarization-dependent gain (PDG)^26^, an issue that persists even in non-integrated SOAs^30^. Research on polarization-insensitive SOAs with carefully designed active layers has reached PDG values down to 0.2 dB^31^, however, commercially available SOAs are mostly still limited to >1 dB. Often, polarization diversity schemes employing polarization optics and two independent amplifiers are used to compensate for the intrinsic PDG of each amplifier, but these come with a far more complex device architecture^32,33^. Low-PDG amplifiers are particularly important in long-haul communication systems, where PDG accumulates across multiple amplifier stages and severely degrades overall channel capacity^11^.

Another important class of integrated optical amplifiers is based on rare-earth-doped (RE) gain media^34–50^. Interest in RE-based integrated amplifiers has grown in recent years, even though they are optically pumped, due to their superior performance compared to semiconductor-based counterparts, particularly in terms of lower noise, higher saturation power, reduced optical nonlinearities, and greater thermal stability. Over the past decades, numerous demonstrations of RE-based integrated amplifiers have been reported. However, due to the tight optical mode confinement typical in standard integrated photonics, the output saturation power (signal output power at which the gain is reduced by 3 dB from its small signal value) in these devices has typically remained in the few tens of milliwatts range, and the on-chip output power has generally been limited to less than 150 mW^49^. More recently, high-power amplification has been demonstrated on a silicon photonic platform using large mode area (LMA) waveguide technology^51–53^. This approach increased the on-chip optical mode area by a factor of 30 or more compared to conventional silicon photonics, while maintaining a >90% overlap with the gain medium. The LMA design enables interaction with a larger number of gain ions over a shorter device length, resulting in significantly increased saturation power and energy storage capacity, while still supporting single-mode propagation. In recent demonstrations^51,52^, such LMA-based devices were optimized for the power amplification regime, designed to amplify seed signals of several milliwatts to well over 1 W of output power on-chip. In these cases, the amplifier length was kept short, the doping concentration was very high, and the maximum net gain was limited to approximately 16 dB, constrained by parasitic lasing due to reflections from the waveguide facets.

In this work, we demonstrate LMA-based silicon photonic amplifiers capable of both high small-signal net gain, reaching up to 30 dB, and high output saturation power of 115 mW, enabling both high-gain amplification and power amplification within a single device. The amplifier length and concentration were optimized to achieve high performance in both amplifier regimes. Typically, in fiber amplifier technology, high-gain amplification and high-power amplification are achieved with separate devices and different amplifier designs. For example, in telecommunication links, pre-amplifiers target high-gain and low-noise amplification of very weak signals in the µW-range just before the receiver. These amplifiers focus on small mode areas to increase optical intensities for more intense gain extraction and longer gain sections to accumulate gain^54^. Power booster amplifiers, on the other hand, aim to amplify already relatively high-power signals from the transmitter in the few-mW range and boost them to several 100 mW or even Watt-level output powers. Such amplifiers employ shorter gain sections and large mode area designs (e.g., LMA or double-clad fiber) to improve gain saturation limitations and reduce signal intensities to avoid non-linear effects. In addition, compared to previous reports^51,52^, we demonstrate an ultra-low on-chip noise figure of ~3.6 dB at 30 dB gain and, importantly, present a method for achieving tunable polarization-dependent gain (PDG) in integrated photonics, with the ability to reduce the PDG to 0 dB. This level of polarization insensitivity surpasses even that of state-of-the-art single-mode fibers, which still exhibit residual PDG due to fabrication imperfections. We demonstrate complete PDG suppression across a wide range of input signal powers, from a few microwatts to several milliwatts, by adjusting either the pump power or the pump polarization state launched into the amplifier. In the following, section “Results” describes the device design and results on high net gain, noise figure, and the concept and results on PDG, and section “Discussion” contextualizes and summarizes the results.

## Results

**Fig. 1:**
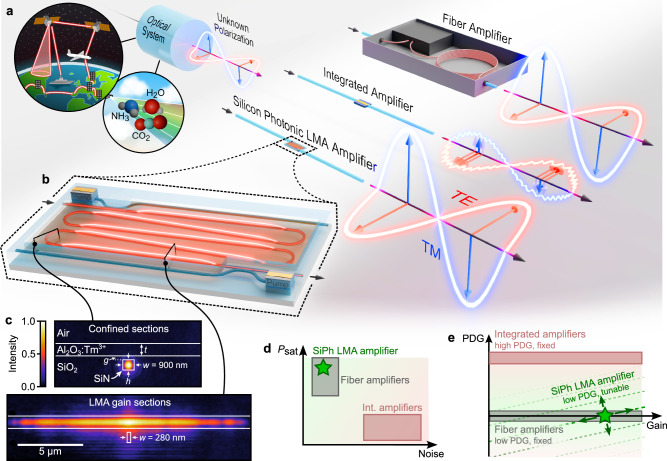
Overview of various amplifier technologies and the structure of an integrated LMA amplifier device. **a** Comparison of different optical amplifier technologies, namely fiber amplifiers, integrated amplifiers such as semiconductor optical amplifiers, and the silicon photonic LMA amplifier, illustrating the differences in power, polarization dependence, and noise of the signal output. **b** A conceptual LMA amplifier with co-integrated pump laser diodes. **c** Measured images of the TE mode at the pump wavelength in the high-confinement sections and gain sections, captured with an IR camera. The material layer stack is also highlighted. **d** Comparison of the typical saturation power vs. noise and **e** the PDG vs. gain of different amplifier types. The LMA amplifier has a high *P*_sat_ with low noise figure and, unlike other integrated amplifiers and fiber amplifiers, can be tuned to a desirable PDG for a large range of net gain values.

A conceptual schematic of the 10.7-cm-long LMA amplifier waveguide with possible future pump diode integration is shown in Fig. 1b and the waveguide cross-section is outlined in Fig. 1c. The waveguide consists of a silicon nitride (SiN) serpentine structure buried in silica (SiO_2_) and an RF-sputtered thulium-doped aluminum oxide top layer (Al_2_O_3_:Tm^3+^), which provides the gain. Exact dimensions of the layer stack are listed in the methods section. Although mainly erbium-doping is of interest for telecom applications, we have chosen thulium-doping due to its various medical, defense, and space applications as well as possible applications in telecommunication at 2 µm exploiting its large gain bandwidth^55–57^. The estimated thulium concentration and passive film loss are 4.0 × 10^20 ^cm^−3^ and ≤0.10 dB/cm at 1.61 μm, respectively. The serpentine amplifier waveguide combines high-confinement sections at the input, the output and the bends with long and straight LMA sections highlighted by the red-tinted waveguide sections in Fig. 1b. The vertical mode transition from high-confinement SiN elements into the Al_2_O_3_ gain layer is realized with adiabatic tapers, allowing for tight bends and a small device footprint <11 mm^2^ as well as the potential for seamless integration with other integrated photonic components. In the LMA sections, the TE-polarized pump (1.61 µm) and signal (~1.85 µm) light propagate mostly in the gain layer with effective mode areas (*A*_eff_) of 23 and 21 µm^2^, respectively. The mode profiles in both sections were measured with an IR camera and are shown in Fig. 1c (see the “Methods” section for details). This device was designed for operation with the fundamental TE mode, because the TM mode is significantly smaller (~9 µm²) and has much more overlap with the rough sidewalls of the SiN waveguide below the gain film leading to higher propagation loss (0.2% overlap with the SiN for TE and 2.2% for TM), which also means that the overall propagation loss in the LMA section is slightly higher than the pure Al_2_O_3_ film loss. A second amplifier waveguide design is presented in the section “Tunable polarization sensitivity”, which features an increased TM mode area, enabling low loss for both polarization modes. An in-band pumping scheme is used with the pump wavelength at 1.61 µm, and the broad emission of the Tm^3+^-doped Al_2_O_3_ provides gain from 1.7 to 2.1 µm. Similar to erbium-doped gain media, the gain with thulium doping can suffer from the energy-transfer upconversion process (ETU), a parasitic ion–ion interaction process^58,59^, which can significantly decrease the excited state lifetime and degrade the amplifier performance^41,60–66^. In our measurements, we have observed the presence of ETU and very small amounts of concentration quenching, although the high-gain benefits of a higher doping concentration far outweighed the negative side effects of ETU and quenching.

### Gain measurements

**Fig. 2:**
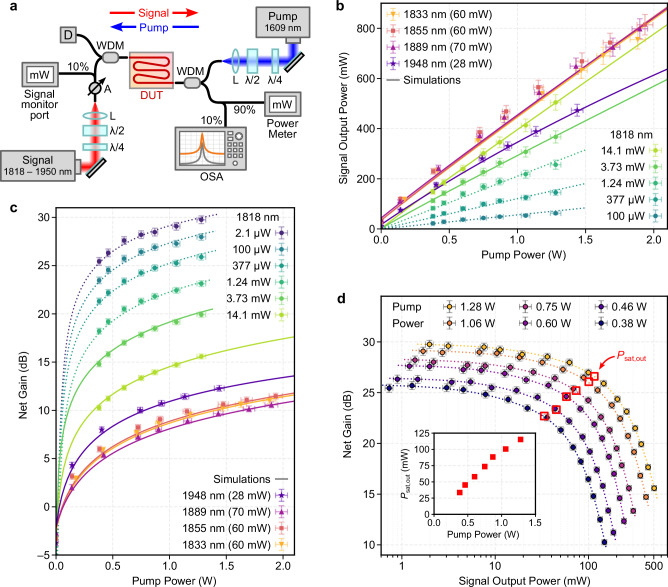
Experimental setup and measured gain, output power, and saturation power. **a** Gain measurement setup with a counter-propagating pump-scheme. **b** Amplified on-chip output power and **c** on-chip net gain as a function of pump power for various signal input power levels (symbols). Solid lines show simulated results for the high-power signals. Dotted lines are only a guide for the eye, as the simulations do not exactly match at low signal power due to not including the impact of forward and backward ASE in the simulations. **d** On-chip net gain versus signal output power for different pump power levels. Dotted lines are a guide for the eye. Red boxes indicate the output saturation power *P*_sat,out_ and the inset shows the *P*_sat,out_ as a function of on-chip pump power. All error bars indicate uncertainties in the fiber-to-chip coupling loss around the mean value estimated from at least five different measurements.

Two sets of gain measurements were performed, for which the measurement setup is shown in Fig. 2a. A counterpropagating pump setup was chosen to decouple pump and signal input. First, high-power amplification tests were carried out with high-power signals at various wavelengths to investigate the power-handling capability and the gain bandwidth of the amplifier. The second set of measurements was performed with low-power signals at a fixed wavelength of 1818 nm. To prevent parasitic lasing at high gain levels (above 16 dB), it is essential to minimize signal reflections from the waveguide facets back into the amplifier. This can be achieved primarily with two approaches: (a) designing input and output couplers with optimized facet angles and mode sizes to reduce reflectivity while maintaining efficient coupling, though this is often difficult to implement effectively for high suppression levels; or (b) applying anti-reflection (AR) coatings or using index-matching fluids that closely match the refractive index of the guided mode at the waveguide facet. To identify a suitable method, we measured the facet reflectivity using an optical frequency-domain reflectometer (OFDR) while testing a variety of index-matching fluids. Each fluid was evaluated individually for its ability to suppress reflections, particularly for the TE mode at 1818 nm, and its stability under high-power operation. From these measurements, we determined the effective refractive index at the facet to be ~1.46 for the TE mode at 1818 nm. We then applied Norland NOA148, an index-matching fluid with a closely matched refractive index, to the waveguide facets. This treatment successfully reduced facet reflections to approximately –35 dB, significantly mitigating the risk of parasitic lasing. For future device implementations, AR coatings are expected to provide a robust, practical alternative with a similar level of reflection suppression.

To accurately determine the on-chip net gain, the signal output power of the 10.7-cm-long amplifier waveguide was compared to the output power of a 2.2-cm-long low-loss passive straight reference waveguide on the same chip (propagation loss in the high-confinement SiN waveguide <0.15 dB/cm). As the taper couplers in both waveguides are identical and the rest of the setup remains unchanged, this method directly provides an accurate value of the on-chip net gain, which is independent of coupling and fiber component losses down the line (i.e., WDM and splitter losses). The on-chip signal power was subsequently calculated using the measured coupling loss (see methods). In this report, the depicted and discussed power levels are always the on-chip power levels, unless stated otherwise. The measured amplified signal power at the output and the on-chip net gain are shown in Fig. 2b and c, respectively, as functions of pump power for various signal input power levels and wavelengths. On-chip output powers reached up to 815 mW (335 mW fiber-coupled output power) with high-power input signals from 60 to 70 mW at 1.9 W pump power (3.4 W pump power off-chip). The on-chip power conversion efficiency (signal output power divided by launched pump power) at the maximum signal output is around 43%. High-power amplification to >500 mW was achieved over a broad bandwidth from 1818 to 1948 nm, owing to the broad and nearly flat emission spectrum of thulium. Signs of gain saturation with increasing pump were only noticeable in the 1948 nm signal, because this wavelength is further away from the gain maximum of thulium (1830–1880 nm). Still, a net gain of 12.4 dB was achieved for a 28-mW input signal at this wavelength, before parasitic lasing from the waveguide facets set in and suppressed any further gain at higher pump power levels. Measurements of the thulium emission spectrum further indicate that amplification at even longer wavelengths around 2 µm is possible, but may require small adjustments to the amplifier designs, for example, longer gain waveguides to compensate for the smaller emission cross-section at long wavelengths. Simulated amplifier results are shown as solid lines in Fig. 2b and c and are based on waveguide and mode parameters as well as measured spectroscopic properties, such as the excited state lifetime, emission and absorption cross-sections, and upconversion parameters (see section 4 in the supplementary material for details).

For the small-signal gain measurements at 1818 nm wavelength, index-matching glue was applied to the waveguide facets to prevent parasitic lasing, and the results are also shown in Fig. 2b and c. Signals with input powers ranging from 2.1 µW to 14.1 mW were tested, and a maximum on-chip net gain of 29.8 dB was measured for the lowest input power at 1.28 W pump power before parasitic lasing in the amplifier set. This corresponds to 25.4 dB fiber-to-fiber net gain after considering the 2.2 dB coupling loss per facet. As the signal input power is increased, the achievable net gain begins to saturate (also known as gain compression), because the strong signals significantly deplete the population inversion. This is highlighted in Fig. 2d, where we show the net gain as a function of signal output power. Initially, the small-signal gain is nearly constant for signal output powers up to ~ 10 mW, after which the gain begins to drop noticeably. From this data, we extracted the output saturation power *P*_sat,out_, which is taken as the signal output power at which the net gain has dropped by 3 dB from its small-signal gain value. This performance parameter should not be confused with the saturated output power, which is the maximum output power the amplifier can produce. The *P*_sat,out_ increases roughly linearly with pump power and reaches slightly more than 115 mW at the highest tested pump power. It is connected with the input saturation power via the gain at the saturation point: *P*_sat,out_ = *G*_sat_ × *P*_sat,in_^67^. In this device, the input saturation power is measured to be 0.25 mW and is independent of pump power. The high saturation power can be attributed to the LMA waveguide design, which significantly increases the intrinsic saturation power (*P*_sat_ ~ *A*_eff_) compared to high-confinement silicon photonic waveguides with small mode areas, and allows for the amplification of very high-power signals^3^.

### Noise figure

**Fig. 3:**
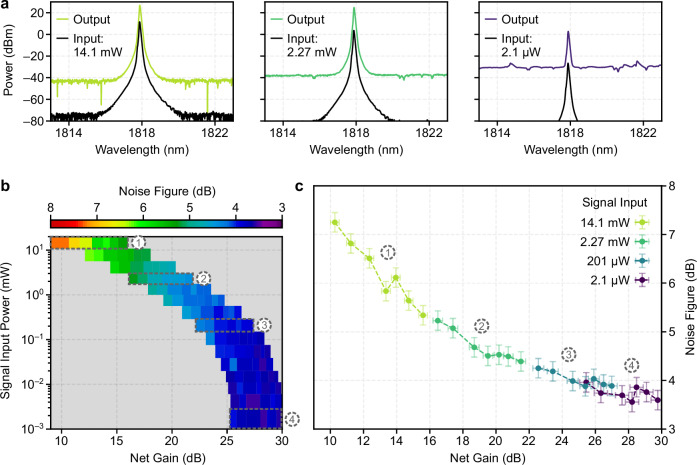
Amplified output spectra and noise figure of the amplifier. **a** Input and output signal spectra for various signal input power levels. **b** Colormap of measured noise figure values as functions of net gain and signal input power. **c** Noise figure values as a function of net gain for selected signal input power levels. The error bars indicate uncertainties in the fiber-to-chip coupling loss around the mean value estimated from at least five different measurements.

The amplified output signal is accompanied by a broad background of amplified spontaneous emission (ASE), as shown in the spectra in Fig. 3a. ASE noise is the main contributor to the noise of optical amplifiers. To quantify the noise properties, we determined the noise figure (NF) with the optical method from the measured output spectra using the relation NF = 2*P*_ASE_/(*G*_lin_*hν B*_0_) + 1/*G*_lin_^54,68^, in which *P*_ASE_ is the ASE power in the same polarization as the signal. *B*_0_ = 0.053 nm is the calibrated OSA equivalent noise bandwidth, *hν* is the signal photon energy, and *G*_lin_ is the gain factor in linear scale (output power divided by input power). The ASE power at the signal wavelength was interpolated from the shape of the ASE spectrum 4 nm away from the signal, which is a good approximation as the ASE spectrum is very flat around the signal. The signal gain and ASE power were adjusted to the on-chip conditions to obtain the on-chip amplifier noise figure. Fiber-to-fiber noise figure values would increase by 2.2 dB compared to the on-chip values due to a degradation of the input signal-to-noise ratio caused by the input coupling loss of 2.2 dB. The output coupling loss has only a negligible effect on the fiber-to-fiber noise figure, because the gain of the amplifier is much higher than the output coupling loss, which we discuss in more detail in Section 3 of the supplementary material.

The measured on-chip noise figure values are shown in Fig. 3b and c, highlighting the dependency on signal input power and on-chip net gain. Values as low as 3.6 dB were measured for small signals <10 µW. This corresponds to a fiber-to-fiber noise figure of 5.8 dB when including the 2.2 dB coupling losses per facet with the current coupler design. The low on-chip noise figure is the result of the high gain (>25 dB) and a low spontaneous emission factor *n*_sp_ ∝ NF, which is given by *n*_sp_ = *σ*_em_*N*_1_/(*σ*_em_*N*_1_− *σ*_abs_*N*_0_), in which *N*_0_ and *N*_1_ are the ground state and excited state populations, and *σ*_em_ and *σ*_abs_ are the emission and absorption cross-sections. The spontaneous emission factor is low because the amplifier is pumped very strongly and is thus well inverted (*N*_1_ » *N*_0_) throughout most of its length, and the weak signals are not strong enough to significantly reduce the population inversion. With higher input power > 100 µW, the amplified signal gets strong enough to deplete the population inversion towards the output section of the amplifier, which reduces the extractable gain and increases the reabsorption loss, leading to a higher noise figure^54^. The optimal noise figure in the high-gain regime may be estimated via NF ≈ 2*n*_sp_. Using measured cross-section values (supplementary Fig. S5) we calculate a lower limit of NF ≈ 2.083 = 3.18 dB. The reason for the low noise figure of the in-band-pumped thulium-based amplifier may be attributed to the low spectral overlap of the pump and emission bands. This results in large differences between *σ*_em_ and *σ*_abs_ at the pump wavelength, which enables a high population inversion as *N*_1_/(*N*_0_ + *N*_1_) ≈ *σ*_abs_/(*σ*_em_ + *σ*_abs_), and at the signal wavelengths leading to the low *n*_sp_. Low noise figures very close to the high-gain signal-spontaneous noise quantum limit of 3 dB have also been demonstrated with an in-band pumped TDFA^69^. This is, for example, very challenging with in-band-pumped erbium-doped amplifiers, as the emission and absorption bands have a much stronger overlap. Therefore, *σ*_em_ and *σ*_abs_ are not very different at the usual pump (~1480 nm), and signal wavelengths (~1550 nm) and noise figures are typically noticeably higher than 3 dB^67^. Improved noise figures can be achieved by pumping the erbium ions to a higher excited state using, for example, a 980 nm pump laser (or a 790 nm pump laser in the thulium case), which allows for an inversion approaching unity and noise figures very close to 3 dB^67,70^.

Lastly, the fiber-to-fiber noise figure can be improved significantly by optimizing the fiber-to-chip coupler design. In this case, the input coupling loss was 2.2 dB due to a non-optimal mode-matching condition at the waveguide facet with the index-matching glue, which led to an increase of the fiber-to-fiber noise figure to 5.8 dB. With an improved coupler design, the coupling loss can be reduced significantly, and losses down to 0.2 dB per facet have been demonstrated^71^, which could reduce the fiber-to-fiber noise figure of a packaged amplifier device to below 4.0 dB.

### Tunable polarization sensitivity

**Fig. 4:**
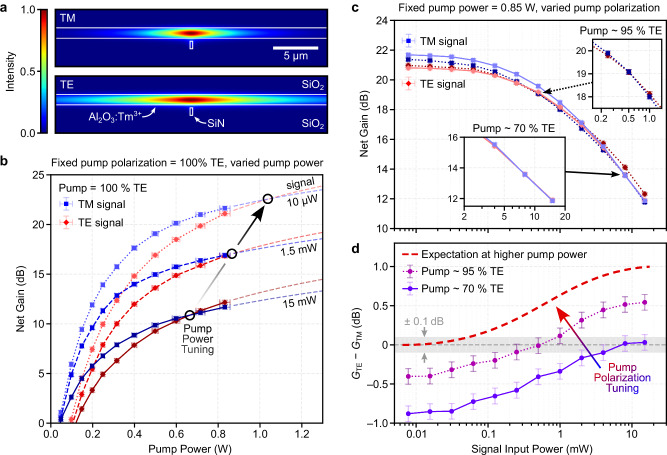
Demonstration of a tunable polarization dependence with the LMA amplifier. **a** Mode profiles of the TM and TE modes in the LMA gain section of the tunable-PDG-amplifier. **b** Measured on-chip net gain of TE (red, diamonds) and TM (blue, squares) signals as a function of coupled pump power for three signal input powers. In this measurement, the pump light was fully TE polarized. Faint dashed lines are extrapolations of the gain data at higher pump power. 0-dB-PDG points are marked with circles. **c** Measured on-chip net gain of TE signals (red, diamonds) and TM signals (blue, squares) as a function of signal input power with two different mixed pump polarizations (solid lines: 95% TE/5% TM; dotted lines: 70% TE/30% TM). Here, the on-chip pump power was fixed at 0.85 W. **d** Difference between TE and TM signal gain (PDG) extracted from the data shown in **c**. The dashed line is the expected PDG curve with 100% TE pump polarization and ~20% more pump power, to achieve 0 dB PDG at low signal power, which was extrapolated from the data in **b**. All errorbars indicate uncertainties in the fiber-to-chip coupling loss around the mean value estimated from at least five different measurements.

A second amplifier device was fabricated to demonstrate tunable polarization dependence with the integrated LMA technology. Here, we define the polarization-dependent gain (PDG) as the gain difference between a TE signal and a TM signal, PDG = *G*_TE_–*G*_TM_, specifying that positive PDG values indicate stronger TE gain, while negative values indicate stronger TM gain. The device was fabricated with a slightly thicker gain layer and a protective SiO_2_ top cladding, leading to large mode areas for both the TE and TM polarized fundamental modes (TE: 57 μm^2^, TM: 26 μm^2^) and thus high gain for both polarizations. Simulated mode profiles are shown in Fig. 4a. The TE mode experiences slightly more propagation loss than the TM mode, mostly due to a larger overlap with the lossy SiO_2_ top cladding and to a lesser extent due to a higher probability of interacting with any defects in the gain layer because of the larger mode, which can be improved with film deposition by reducing film stress and microcracks. Due to the different mode sizes, the TE and TM signal gains are not generally identical but can be equalized to achieve polarization-independent gain (PDG of exactly 0 dB) by tuning the pump parameters, specifically the pump power and pump polarization. Tuning of the pump conditions changes the spatial overlap of the pump mode with the TE and TM signal modes, since the two are of very different mode sizes, leading to a differential change in gain for the two polarizations.

To investigate this polarization-dependent behavior, we measured the net gain of TE and TM signals at three signal power levels ranging from 10 µW to 15 mW with TE polarized pump light, and the data is shown in Fig. 4b. The same measurements were also repeated with TM polarized pump light, but are not shown here for clarity in the graphs (more details in supplementary section 5). Initially at low pump power, the TM signal gain is stronger than the TE gain, mainly due to the slightly higher loss with the larger TE mode. Additionally, the smaller TM mode overlaps mostly with the high-intensity parts of the TE pump mode, where the population inversion is highest, while the TE signal mode also overlaps with the low-intensity wings of the pump mode, which leads to more reabsorption loss for the TE signal. However, as the pump power is increased, the gain medium is well inverted even in the wings of the pump mode, and the large TE mode effectively interacts with more excited ions to extract more gain than the TM mode. This behavior has a noticeable dependency on the signal power, because the point where the TE gain overtakes the TM gain shifts to higher pump power when the signal power is lowered, as highlighted in Fig. 4b. This is the result of the higher saturation power of the TE signal due to the larger mode area (*P*_sat_ ~ *A*_eff_), meaning that a high-power TE signal can still extract more gain while the TM signal is already saturated (gain compression). This is not the case with low signal power, where both the TE and TM signals are far from compression, and therefore the TM gain remains high due to the advantages mentioned above. As a result of the crossing of the TE and TM gain curves, one can find a specific pump power for any given signal power at which the PDG is exactly 0 dB, and the determination of this point is only limited by the accuracy of the gain measurement. For example, with this device, the 0-dB-point is around 0.66 W pump power for a high-power 15 mW signal, which can be tuned to 0.86 W pump power for an intermediate signal power of 1.5 mW and may be further tuned to ~1.03 W pump power for a low-power 10 µW signal. We could not confirm the small-signal 0-dB-point directly by measurement, because we avoided pump powers >0.9 W since the index-matching fluid on the waveguide facets began to degrade from the prolonged high-power pumping, which can be circumvented with optimized high-power index-matching glue, angled inverse taper couplers, or antireflective coating. Nonetheless, from the measured data up to this pump level, the 0-dB point can be extrapolated.

Further tests confirmed that clean TE and TM signal input polarizations correspond to the polarization orientations for minimum and maximum gain extraction at every pump condition. Any arbitrary input polarization falls in between the two extremes, converging to the same zero-PDG point (more details in supplementary section 5.4).

Alternatively, with the same device, the PDG can also be tuned by changing the polarization state of the pump light while keeping the pump power fixed. We demonstrated this by measuring the TE and TM signal gain with a fixed pump power (0.85 W on-chip) and two mixed polarization states of the pump. In these measurements, we explicitly tested the dependence on the signal power ranging from 8 µW to 15 mW, and the results are shown in Fig. 4c, d. The first pump polarization state was mostly TE-polarized (~95%), which was optimized for 0 dB PDG around 0.5 mW signal power. For the second pump state, we tuned the polarization more towards TM (~70% TE, 30% TM) for an optimized PDG at 10 mW signal power. The shift of the PDG with varying pump polarization is the result of the spatial overlap of pump and signal modes. The best gain for each signal polarization is achieved when pump and signal are in the same polarization, as then both modes are of similar size and overlap well, while the gain is lowest when pump and signal are in orthogonal polarizations. This effect can be used to lower the gain of one signal polarization while simultaneously increasing the gain for the other. For example, with this device at 0.85 W pump power and TE pump polarization, the high-power signal gain is slightly stronger in TE than in TM signal polarization. To equalize the gain, the pump can be tuned to be partially TM polarized, which effectively reduces the pump mode overlap with the TE signal and improves the overlap with the TM signal, thus bringing the gain for both polarizations closer together. This is highlighted in Fig. 4d, demonstrating how the PDG can be optimized for various signal powers by tuning the pump polarization. Achieving a PDG of 0 dB at low signal powers ~10 µW with this device would require ~20% more pump power, as extrapolated from the data in Fig. 4b.

When operating in the high-gain regime (>0.8 W pump power in this case), which is typically desired for the benefits of low noise, the PDG stays below 1 dB over a large range of signal powers from 8 µW to 15 mW (>30 dB range). After optimization via pump power or polarization tuning, the PDG is robust against small signal power fluctuations with PDG variations of <±0.1 dB within a ~6 dB range of signal powers. If required, the PDG may also be increased significantly by lowering the pump power and tuning the polarization to mostly TM. In this case, depending on the signal power, the TM signal gain can be up to 8 dB stronger than the TE gain (more details in supplementary section 5). To make the amplifier overall more polarization-insensitive, the LMA waveguide design can be optimized to make the TE and TM modes similar in size and gain saturation behavior. This would stabilize the zero-PDG point more against signal power fluctuations, but it would also limit its tunability. While tuning of the pump power can be realized rather effortlessly in a fully integrated amplifier device, e.g., with hybridly integrated pump laser diodes, the polarization tuning on-chip remains challenging. An approach may be to integrate two pump diodes with orthogonal polarization, either bidirectionally or codirectionally, using an on-chip polarization combiner^72^, such that the overall pump polarization state can be tuned through the power of each diode.

### Long-term power stability

**Fig. 5:**
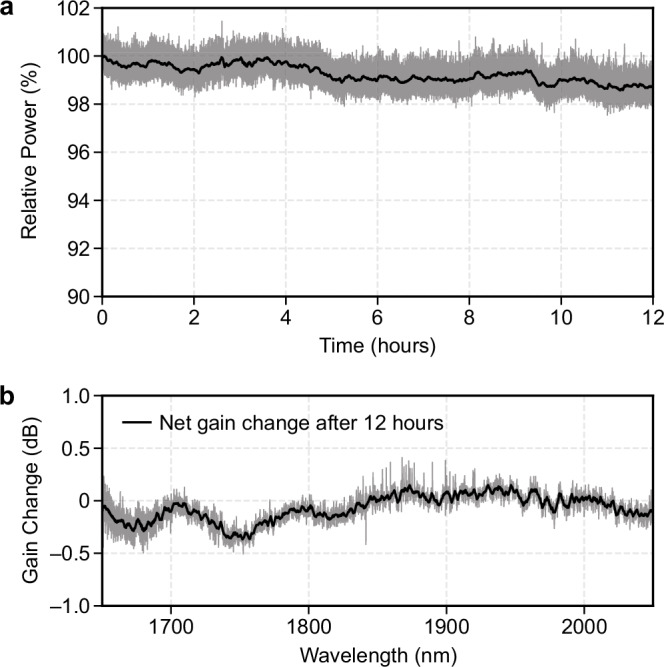
Long-term power stability. **a** Amplified signal output power of a 6.7 cm-long amplifier device with 0.5 mW signal input power and continuous high-power pumping with 0.7 W on-chip pump power. The small drop of power over time is due to slight fiber-to-chip coupling misalignment due to thermal drift of the coupling stage. Shown in light gray is the output power taken in 300-µs-intervals, which shows fast fluctuations due to seed laser intensity noise. Shown in black is the power averaged over 10 s. **b** Change of the net gain before and after the 12-h long test. Shown in light gray is the data taken on the spectrum analyzer, while the black line is a post-processed moving average to improve visual clarity.

We have furthermore performed an uninterrupted 12-h-long gain test on a 6.7-cm-long amplifier device to demonstrate the long-term power stability under continuous high-power pumping. Applying index-matching glue with a high viscosity (Norland NOA 148) helped to reduce thermal drifts and mechanical vibrations in the fiber coupling setup, which would otherwise move out of alignment within a few minutes and make prolonged stability tests without glue very difficult. For this gain test, we used a supercontinuum laser as the seed laser with a total coupled signal power of 0.5 mW and 0.7 W on-chip pump power, where we observed the amplification in the entire thulium emission band from 1650 to 2050 nm. We monitored the amplified signal output power on a power meter (Thorlabs PM100D) in 300-µs detection intervals. The relative output power over time compared to the start of the test is shown in Fig. 5a. A slow power drop of about 1% can be observed, which was due to a still small drift of the coupling setup over time, which could be fully restored by reoptimizing the fiber-to-chip coupling afterward. Figure 5b shows the change in the gain spectrum before and after the 12-h test. Small fluctuations in the gain spectrum are due to a power instability of the signal laser, which is transferred to the wavelength-axis due to the slow wavelength sweep speed of the spectrum analyzer. This test followed several weeks of high-power gain tests on the same device without any signs of gain or material degradation, indicating the suitability for extended field deployment of the integrated Al_2_O_3_ gain medium.

## Discussion

The results show that a single integrated LMA amplifier is suitable for both small-signal amplification with high net gain and low noise figures as well as high-power amplification with signal powers approaching the watt-level. Such high power handling, together with the capability to tune the PDG to exactly 0 dB, are two aspects that were previously not achievable with high-confinement silicon photonic amplifiers due to typically low saturation powers and high polarization sensitivity. The broad emission spectrum of the thulium gain ions enables very broadband amplification up to 2 µm in wavelength, making the amplifier suitable for multiple applications, especially in gas sensing and medical fields due to absorption lines of several molecules in this spectral range, such as H_2_O, CO_2_, or NH_3_. Output saturation powers > 115 mW (at the 3-dB gain compression point) have been demonstrated with this device, which is almost one order of magnitude higher than any other integrated amplifier to date. Higher saturation powers can be achieved by increasing the mode area even further with a thicker gain layer and a thicker interlayer oxide between the SiN and gain layer. With this, mode areas >100 µm^2^ are achievable. Such large mode areas are beneficial for high-power amplification within a short length, as in power amplifier operation, but are less desirable for small-signal amplification, where longer devices are needed for the gain to build up. The power conversion efficiency with the 10.7 cm-long amplifier tested here was limited to 43%, which can be improved to >50% and even ~60% by reducing the total propagation losses and optimizing the length and doping concentration, as we have recently demonstrated with a short, high-concentration device^51,52^. Despite the high thulium concentration, the effects of energy-transfer upconversion and concentration quenching had only a negligible impact on the amplifier performance, implying that the concentration may be further increased to improve the gain even more.

The low noise figure and dynamic tuning of PDG are interesting for applications in telecommunication, where currently erbium-doped fiber amplifiers (EDFA) deliver high-quality signal amplification but are envisioned to be replaced by more power and space-efficient chip-scale devices for certain applications. The current amplifier is fabricated with thulium doping, but future devices will also employ erbium doping, which has already been demonstrated with great success in the aluminum oxide host material^50^. Combined with the LMA waveguide architecture and further design optimization, we expect similar, if not better, high-power and low-noise amplification in the C-band that is on par with EDFA in performance, while reducing the device footprint by several orders of magnitude. Our proof-of-concept measurements demonstrate the ability of the LMA amplifier to tune the PDG to exactly 0 dB for a wide range of signal power levels by modifying the pump power or pump polarization, even when the modes are of different sizes (unlike in fiber amplifiers and SOA). This is a big challenge in integrated SOAs and high-confinement RE-based amplifiers, where the PDG is fixed for a given device geometry and pump conditions, and the PDG cannot be easily adjusted once fabricated. Further tests are planned to explore the effects of polarization hole burning (PHB) in the LMA amplifier, which is a main driving mechanism behind non-zero PDG in amplifier chains^73,74^. With an adjusted design, the PDG can also be increased significantly to suppress one signal polarization compared to the other. For example, with a thinner gain film, the TM mode has a stronger overlap with the rough SiN waveguides, which leads to much higher propagation loss compared to the TE mode. This can lead to a PDG > 15 dB, which effectively turns the amplifier into a polarizing element heavily favoring TE mode propagation and gain.

In conclusion, we demonstrate an integrated thulium-doped amplifier based on large mode area (LMA) technology, achieving both high net-gain up to 30 dB and high output saturation power exceeding 115 mW from a single device. The amplifier also exhibits a low noise figure, reaching 3.6 dB at 30 dB net gain. Additionally, we introduce a method for tunable polarization-dependent gain (PDG) across a broad range of input signal powers. Our current implementation enables precise tuning of the PDG to 0 dB, and even under fixed pump conditions, the PDG remains below 0.1 dB across a wide input power range. These results represent a significant advancement toward low-noise, polarization-insensitive integrated amplifiers, paving the way for their use in long-range telecommunications and other demanding optical systems.

## Methods

### Chip layer stack and fabrication

The dimensions of the chip layer stack shown in Fig. 1c are *h* = 800 nm for the SiN thickness, *t* = 1000 nm for the Al_2_O_3_ gain layer thickness, and *g* = 300 nm for the SiO_2_ spacing layer between the SiN and gain layers. The waveguide width in the high-confinement sections is *w* = 900 nm, and in the LMA section it is *w* = 280 nm. In the device used for the PDG characterization, the gain layer was slightly thicker at *t* = 1100 µm, and a 1 µm-thick SiO_2_ top cladding layer was added. All passive components were fabricated in a silicon-nitride foundry (LIGENTEC SA), and the gain film was deposited using reactive RF co-sputtering at the University of Twente (more details on the fabrication process are provided in section 1 of the supplementary material). The images of the modes shown in Fig. 1c in the LMA and confined sections were captured with two aspheric lenses, one with 8 mm focal length to collimate the waveguide output and the other with 300 mm focal length to create the image on an infrared camera (ICI SWIR 320).

### Details of the measurement setup and coupling losses

The gain measurement setup (see Fig. 2a) consists of a high-power CW pump laser at 1609 nm wavelength and a homebuilt high-power tunable CW seed laser based on a Tm-doped YLF crystal, tunable from 1830 to 1950 nm via a birefringent filter (ref. ^75^ for details). Alternatively, an interference-based filter was used to tune to shorter wavelengths (1818 nm). Signal and pump light polarizations were controlled via half- and quarter-wave plates before coupling into fiber-based wavelength division multiplexers (WDM) to obtain the desired polarization on-chip. The signal output power was monitored on a power meter through the 90% port of a fiber-based power splitter, and the amplified spectra were recorded on a calibrated optical spectrum analyzer (OSA) through the 10% port. The fiber-to-chip coupling loss was determined from the total insertion loss of a 2.2-cm-long passive waveguide next to the amplifier waveguide on the same chip. For the high-gain device, the coupling losses were 2.6 dB per facet for the pump and 3.9–4.2 dB for the signal light (1830–1950 nm) in TE polarization. With the index-matching glue (Norland NOA 148) applied, the coupling losses were measured to be 2.0 dB for the pump and 2.2 dB for the signal light. For the tunable PDG device, a different index-matching glue was applied (Luvantix SH-548HT), and the coupling losses were 4.7 and 7.1 dB for the TE and TM polarized pump light, and 3.9 and 4.9 dB for the TE and TM polarized signal light, respectively.

## Supplementary information

Supplementary Information

Transparent Peer Review file

## Data Availability

The data generated in this study, as well as, the data analysis code, have been deposited in the Zenodo repository under accession code 10.5281/zenodo.20072573.
