# Supplementary material for: Zero-PDG silicon photonic amplifier with high saturation power and low noise figure
Source: Nat Commun. 2026 Jun 30;17:5670. doi: 10.1038/s41467-026-74486-y (PMC13319230; doi:10.1038/s41467-026-74486-y)
Supplement: Supplementary file 1 — Supplementary Information [file 41467_2026_74486_MOESM1_ESM.pdf]

Supplementary Material for:

Zero-PDG silicon photonic amplifier with high saturation power and low noise figure

Jan Lorenzen[1,2,#], Kai Wang[3], Muharrem Kilinc[1], Mikhail Pergament[1], Sonia M. Garcia-Blanco[3], Franz X. Kärtner[1,2] and Neetesh Singh[1,*]

[1] *Center for Free-Electron Laser Science CFEL, Deutsches Elektronen-Synchrotron DESY, Germany*
[2] *Department of Physics, Universität Hamburg, Jungiusstr. 9, 20355 Hamburg, Germany*
[3] *Integrated Optical Systems, MESA+ Institute for Nanotechnology, University of Twente, 7500AE, Enschede, The Netherlands*
*# jan.lorenzen@desy.de, * neetesh.singh@desy.de*

1. Device design and fabrication details

A cross-section of the amplifier waveguide is shown in Fig. S1a. The waveguide consists of a bottom silicon nitride (SiN) layer buried in silica (SiO_2) and a thulium-doped aluminum oxide (Al_2O_3:Tm^{3+}) top layer, which provides the gain. The SiN layer thickness is h = 800 nm and the top gain layer is t = 1000 nm thick. Between the SiN and the gain layer is an interlayer oxide spacer with thickness g = 300 nm. All the passive components of the device (all layers except for the Tm-doped Al_2O_3) were fabricated in a silicon photonics foundry (Ligentec SA) on a silicon-nitride-on-silicon platform. The SiN waveguide patterns were created with deep-UV lithography. The 300 nm thick interlayer oxide was obtained by etching a 3.3-µm-thick top oxide layer down to the desired thickness within a designated region to create a local oxide opening, which is highlighted as the Tm-doped region (yellow box) in Fig. S1b. Finally, the Al_2O_3:Tm^{3+} gain layer was deposited with a radiofrequency (RF) sputtering tool (AJA ATC 1500) at a deposition rate of 5 nm/min, with the substrate heater set to 700°C (corresponding to an estimated substrate temperature of approximately 400-450°C based on earlier calibration of the system) University of Twente. Special care was taken to deposit the film as cleanly as possible. An up-sputtering configuration was chosen to reduce the risk of flaking or dust falling onto the sample surfaces. For this purpose, the samples were secured in a holder which was mounted to a rotating heated holder mount. The samples in the holder are introduced through a loadlock. A base

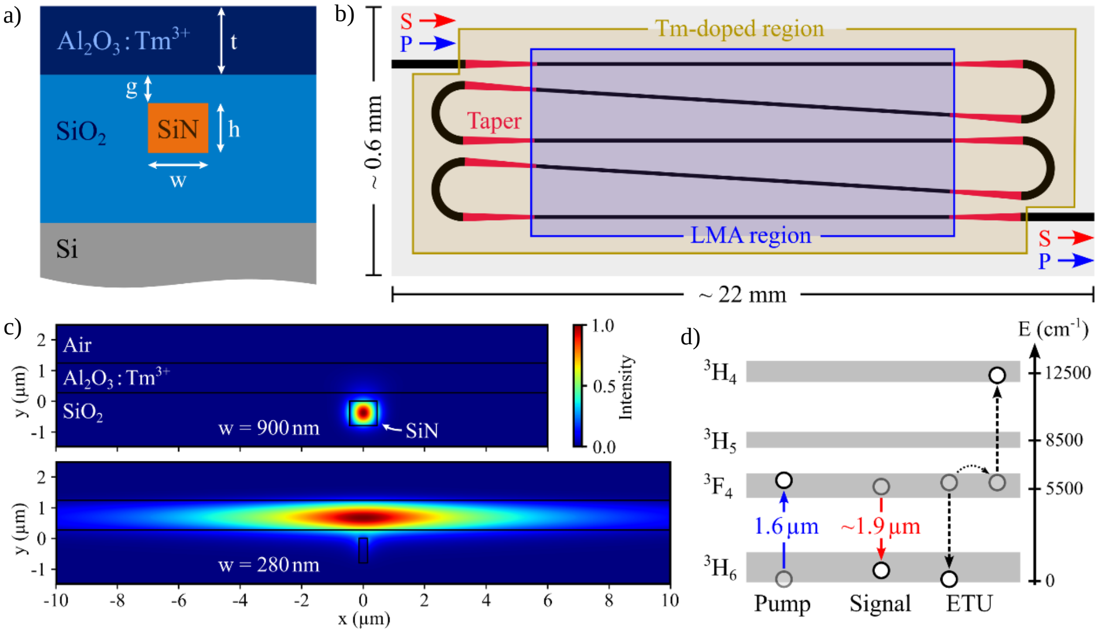

Fig. S1: LMA amplifier design. a) Cross-section of the chip. b) Schematic of the on-chip amplifier. c) Simulated profiles of the TE mode in the confined and LMA regions at 1850 nm wavelength. d) Simplified energy level diagram of Tm^{3+} (ETU: energy-transfer upconversion).

vacuum pressure of 0.1 µTorr is achieved with a turbomolecular pump. Subsequently, argon and oxygen gas are injected at 25 and 3 sccm flow rate, respectively. The reactive co-sputtering process used separate metallic aluminum and thulium targets which together with the optimized injection flow rate of oxygen gas create a high-quality oxide film on the sample. Flexible doping concentrations can be achieved by controlling the thulium sputter rate via the power applied to the thulium target, which ranges from 15 to 21 W depending on the desired concentration. The aluminum target is powered with 200 W RF. The final doping concentration in the film was calibrated with RBS measurements and cross-checked with optical loss measurements using a prism coupling instrument (Metricon 2010/M) with a laser in the thulium absorption window to extract the concentration-dependent absorption loss. The thulium concentration estimated in this way was 4.0×10^{20} cm^{-3} and the passive film loss was ≤ 0.10 dB/cm at 1.61 µm. More details on the gain film deposition and optimization process are outlined in the supplementary material of reference [1] and in reference [2].

The serpentine amplifier waveguide structure consists of high-confinement sections at the input, the output and the bends with a SiN width w = 900 nm, as well as low-confinement LMA regions in the long straight sections with w = 280 nm, highlighted by the blue box in Fig. S1b. High-confinement and LMA sections are connected via adiabatic tapers (highlighted in red). The high confinement allows for tight bends with a radius of 85 µm and bending losses < 0.001 dB per 180° bend to achieve a device footprint < 12 mm^2. In the LMA sections, the pump (1.61 µm) and signal (~ 1.85 µm) light propagate mostly in the gain layer with gain overlap factors of 89.5 and 86.3% and effective mode areas of 22.8 and 21.3 $µm^2$, respectively. Simulated mode profiles are shown in Fig. S1c and the overlap of the pump and signal mode is > 98%. This device was designed for operation with the fundamental TE mode, but the same principle can also be applied for TM operation [1,3]. The mode area may be further increased with a thicker gain layer (t) and thicker interlayer oxide (g), and mode areas > 100 $µm^2$ are achievable. The total length of the amplifier device is 10.7 cm. An in-band pumping scheme is used with the pump wavelength at 1.61 µm, which helps to reduce the quantum defect (the difference between pump and signal photon energies) and thus improves the conversion efficiency compared to pumping at 780 nm [4]. The broad energy levels of thulium-doped aluminum oxide also allow for a relaxed choice of the pump wavelength between 1.55 and 1.65 µm and provide broad signal generation from 1.7 to 2.1 µm. A simplified energy level diagram of thulium is shown in Fig. S1d, highlighting also the energy-transfer upconversion process (ETU) as a parasitic ion-ion-interaction. During ETU, an excited ion transfers its energy non-radiatively to another nearby excited ion through dipole-dipole interactions [5,6]. The second ion is then upconverted to a higher excited state while the first ion drops to the ground state. Subsequently, the upconverted ion might drop to the ground state or to the first excited state. It can also initiate a cross-relaxation (CR) process (which is essentially the inverse of the ETU process) and repopulate the first excited state [4]. Overall, the ETU process effectively depopulates the excited state, which can significantly degrade the amplifier performance and decrease the excited state lifetime [7–14].

2. Gain measurement details

2.1 Measurement setup

The gain measurement setup is shown schematically in Fig. S2. It consists of a high-power CW pump laser at 1609 nm wavelength (Alnair labs TLG 220 with IPG EAR-10-1610-LP-SF 1.6 µm amplifier) and a homebuilt high-power tunable CW seed laser based on a Tm-doped YLF crystal, tunable from 1830 to 1950 nm via a birefringent filter [15]. Alternatively, an interference-based filter was used to reach shorter wavelengths (1818 nm) at the expense of lower signal power. A variable attenuator was used to control the signal input power, which was also monitored on a 10% tap. Pump and signal light were combined with fiber-based wavelength multiplexers and then coupled to the 10.7 cm long amplifier waveguide via AR-coated lensed fibers with 2.5 µm spot size. A counterpropagating pump setup was chosen to decouple the pump and signal input and allow for the separate optimization of pump and signal

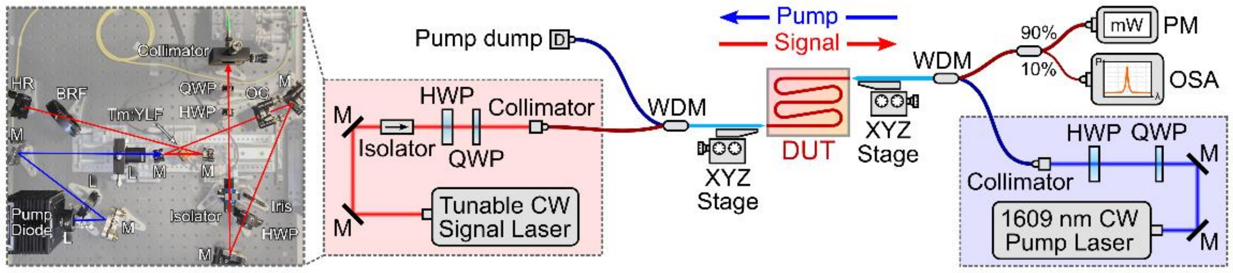

Fig. S2: Detailed amplifier characterization setup. Schematic of the gain measurement setup. Red lines indicate signal light paths, blue lines indicate pump light paths. The left box shows a top-down photo of the homebuilt tunable Tm:YLF laser with annotations of all components. Abbreviations: M = mirror; L = lens; HR = high-reflective mirror; BRF = birefringent filter; QWP = quarter-wave plate; HWP = half-wave plate; OC = output coupler; WDM = wavelength division multiplexer; PM = powermeter.

coupling. Both signal and pump light polarization were controlled via half- and quarter-wave plates to obtain TE polarization on-chip. The signal output power was monitored on a power meter through a 90% port of a fiber-based power splitter and the amplified spectra were recorded on a calibrated optical spectrum analyzer (OSA, Yokogawa AQ6376) through the 10% port.

Two sets of gain measurements were performed. First, high-power amplification tests were carried out with high-power signals at various wavelengths to investigate the power-handling capability and the gain bandwidth of the amplifier. The second set of measurements were performed with low-power signals at a fixed wavelength of 1818 nm, which required small adjustments to the setup. For one, the birefringent filter was replaced with an interference-based filter, which lowered the signal power but improved the wavelength and power stability of the seed laser. Additionally, index-matching glue (Norland NOA148) was applied to the tip of the lensed fibers and waveguide facets to reduce facet reflections from -15 dB to roughly -32 dB. This significantly lowered the threshold for facet-reflection-based lasing in the amplifier, which is crucial when operating in the high-gain regime. It should be noted that such reflections can also be avoided by using angled taper couplers instead of straight couplers, which can reduce facet reflections to -35 dB. The fiber-to-chip coupling loss was determined from the total insertion loss of a 2.2 cm long passive waveguide with identical taper couplers and no fiber components on the input and output side (no WDM nor splitters). With this, the only contributions to the waveguide insertion loss are the input and output coupling losses and the propagation loss of the waveguide (estimated to be 0.2 dB/cm), resulting in 2.6 dB coupling loss per facet for the pump and 3.9 – 4.2 dB for the signal light (1830 – 1950 nm). With the index-matching glue applied, the coupling losses were measured to be 2.0 dB for the pump and 2.2 dB for the signal light.

To determine the on-chip net gain, the signal output power of the 10.7 cm long amplifier waveguide was compared to the output power of a 2.2 cm long passive reference waveguide on the same chip. As the taper couplers in both waveguides are identical and the rest of the setup remains unchanged, this method directly provides an accurate value of the on-chip net gain, which is independent of coupling and fiber component losses down the line (WDM and splitter losses). To calculate the on-chip signal output power, first the on-chip input power was determined from the signal power in the lensed fiber adjusted by the input coupling loss, and subsequently the before-measured net gain was added to obtain the on-chip output power. This approach also has the advantage of being independent of fiber component losses, which would have to be measured at every signal wavelength, but it requires accurate measurements of the coupling losses and net gain. Nevertheless, the obtained output power values were double-checked for the small-signal measurements at 1818 nm by independently measuring the loss contributions of the WDM and the 90:10 splitter on the signal output side, which resulted in the same power values to < 0.2 dB uncertainty and confirmed the validity of the gain-based approach. In this report, the depicted and discussed power levels are always the on-chip power levels, unless stated otherwise.

2.1 Measuring gain with the signal enhancement method

A different approach of measuring the small-signal net gain at 1818 nm wavelength was also tested, which is based on the signal enhancement through the amplifier. Here, the signal output power of the pumped amplifier (P_{on}) is compared to the un-pumped case (P_{off}), which is called the signal enhancement (SE = P_{on} - P_{off}, in dB-scale). In the un-pumped case, the signal experiences not only propagation loss (α_{prop}), but also absorption loss (α_{abs}) from the ground state ions. When the amplifier is pumped, most of the ions are excited and thus the absorption loss is converted into gain at the signal wavelength. Therefore, the device net gain (G) in dB-scale can be calculated with $G = \mathrm{SE} - \alpha_{abs} L_{act} - \alpha_{prop} L_{tot}$, in which L_{tot} = 10.7 cm is the total length of the amplifier and L_{act} = 9.83 cm is the length of the active waveguide part, in which the light interacts with the rare earth ions [16]. The propagation loss in the amplifier at the signal wavelength was extracted from the power-dependent transmission of a 6.5 cm long device at 1855 nm wavelength, as shown in Fig. S5a, resulting in α_{prop} = 0.22 dB/cm. The absorption loss was calculated using the relation $\alpha_{abs} = 10 \log(e)\, \Gamma N_0 \sigma_{abs}$, in which $\sigma_{abs} = 0.95\times10^{-21}$ cm^2 is the measured absorption cross-section at 1818 nm, as shown in Fig. S5b, Γ = 86.3% is the mode overlap with the Tm-doped region, which was calculated with Lumerical's FDE solver, $N_0 \approx N_d = 4.0\times10^{20}$cm^{-3} is the ground state population density, which can be assumed to be equal to the doping concentration N_d as long as a weak signal (< 0.1 mW) is used for the absorption measurement, and the factor 10 log(e) transforms the absorption loss coefficient into dB-scale.

The net gain achieved with the signal enhancement method was nearly identical to the method discussed earlier. As an example, the measured signal enhancement of a 2.2-μW input signal at 1818 nm wavelength with 1.24 W pump power was 47.1 dB. The total absorption and propagation losses at this wavelength in the 10.7 cm long amplifier are 14.0 dB and 3.2 dB, respectively. Subtracting the losses from the signal enhancement results in a net gain of 29.9 dB, which is slightly more than the 29.8 dB measured with the reference waveguide method. Care must be taken when using the signal enhancement method with high input power. In this case, the signal itself is strong enough to bleach the amplifier and experience almost no absorption loss, as seen later in Fig. S5a. For example, the

60-mW high-power input signal at 1855 nm wavelength experiences mostly a propagation loss of 3.2 dB and only negligible absorption loss < 0.3 dB. The signal enhancement measured for this signal was 14.4 dB, amounting to 10.9 dB net gain, which also matches well with the 10.8 dB net gain measured with the reference waveguide method.

3. Additional information on the amplifier noise figure

3.1 Noise figure limit estimation for in-band pumped thulium-doped amplifiers

The noise figure (NF) was determined from the measured output spectra by interpolating the ASE power at the signal wavelength and using the relation NF = $2P_{ASE}$ / (G_{lin} $h\nu$ B_0) + 1/G_{lin} [17,18], in which P_{ASE} is the ASE power in the same polarization as the signal within the equivalent noise bandwidth B_0 = 0.053 nm of the optical spectrum analyzer, $h\nu$ is the signal photon energy, and G_{lin} is the linear gain factor (output power divided by input power). ASE in the waveguide is generated only in the guided TE and TM modes and the measured ASE power on the OSA is the combination of both ASE polarizations. However, due to higher output coupling loss for TM polarized light and lower gain for the TM mode in the waveguide, the TE-polarized ASE component detected on the OSA is significantly stronger than the TM component. Our calibrations with polarizers show that the TM-polarized ASE component measured on the OSA is approximately 3.7 dB weaker than the TE component, and this correction factor has been taken into account to use only the TE-polarized ASE power for the noise figure calculation, because only the ASE in the same polarization as the signal can generate the signal-spontaneous beat noise [18]. Noise figure values as low as 3.6 dB were measured for small signals < 10 µW, which is due to the high gain ~ 30 dB and a low spontaneous emission factor n_{sp}, because NF ≈ 2 n_{sp}. The spontaneous emission factor can be calculated using n_{sp} = σ_{em} N_1 / (σ_{em} N_1 - σ_{abs} N_0), in which N_0 and N_1 are the ground state and excited state populations, and σ_{em} and σ_{abs} are the emission and absorption cross-sections. A lower limit to the achievable noise figure may be estimated with NF ≈ 2 n_{sp} assuming a simplified two-level-amplifier system [17]. The highest achievable population inversion is given by N_1 / (N_0 + N_1) ≈ 1 / (σ_{em}/σ_{abs} + 1) in the limit of infinitely high pump power [19]. When using an in-band pumping scheme (as it was used here: 1610 nm pump, 1818 nm signal), the level of population inversion is limited by stimulated emission at the pump wavelength. Thus, even an infinite amount of pump power cannot excite every gain ion. With the emission and absorption cross-sections σ_{em} = 0.35×10^{-21} cm^2 and σ_{abs} = 2.1×10^{-21} cm^2 at the pump wavelength, this leads to a maximum population inversion of N_1 / (N_0 + N_1) ≈ 86%. At this level of inversion and with the cross-sections σ_{em} = 4.0×10^{-21} cm^2 and σ_{abs} = 0.95×10^{-21} cm^2 at the signal wavelength, this results in a lower limit of NF ≈ 2.083 = 3.18 dB. The reason for the low noise figure of in-band-pumped thulium-based amplifiers may be found in the strong separation of pump and emission bands. This results in large differences between σ_{em} and σ_{abs} at the pump and signal wavelengths, leading to the low n_{sp}. This is for example not the case with in-band-pumped erbium-based amplifiers, as the emission and absorption bands have a much stronger overlap. Therefore, σ_{em} and σ_{abs} are not very different at the usual pump (1470 – 1490 nm) and signal wavelengths (~ 1550 nm), which typically limits the population inversion to N_1 / (N_0 + N_1) < 75 % and noise figures are usually > 4.0 dB. This number can be improved by pumping to a higher excited state using e.g. a 980 nm pump laser, which allows for an inversion approaching unity, as the pump absorption and emission bands are now spectrally separated, but the n_{sp} still suffers from the high absorption cross-section at the signal wavelength, which increases re-absorption losses.

3.2 Fiber-to-fiber noise figure and calculation with Friis' formula

The noise figure was extracted with gain and power levels calibrated to the on-chip conditions. This means explicitly, that the signal input power was considered to be the coupled on-chip input power, and for the signal output power, the output coupling loss and additional fiber component losses after the amplifier were added back to the power levels measured on the OSA to obtain the on-chip signal output level and on-chip gain. The coupling loss per facet was 2.2 dB and the total losses on the output side (output coupling loss, WDM loss, spectrum measured on a 10% drop port, connector losses, and insertion loss to the OSA) were measured to be 25.4 dB. Alternatively, we also used the measured fiber-to-OSA gain and power levels to obtain the fiber-to-OSA noise figure and subsequently calculate the corresponding on-chip noise figure by de-embedding the coupling losses and fiber component losses according to Friis' formula. We saw that both approaches lead to the same results. In the following we would like to discuss in more detail the differences between on-chip, fiber-to-fiber and fiber-to-OSA noise figure calculation.

As we understand, it is important to specify the relevant reference planes for the input and output power level of the signal and ASE to properly evaluate either the on-chip or fiber-to-fiber NF value. In a practical implementation of the amplifier system, all external losses can ideally be reduced to only the fiber-to-chip coupling loss of 2.2 dB. Therefore, for the fiber-to-fiber noise figure, we consider only the input and output coupling fibers as the reference

planes, while all the extra fiber component losses on the output side for performance monitoring were combined into a correction factor to the measured power levels. We understand that most important are any losses before the amplifier, as any lossy element would lead to a degradation of the input SNR equal to the loss of the element [20]. As the input coupling loss to the chip is 2.2 dB, this would already lead to an increase of the fiber-to-fiber NF by 2.2 dB. Any lossy element after the amplifier has to be treated with Friis' formula for cascaded amplifier/loss components. We also compare this with a similar example in [20]. As we noticed, the additional loss after the amplifier has only a minor effect on the total noise figure of the system, unless the additional output loss becomes similar in size to the amplifier gain. We use Friis' formula for the total noise factor of a cascade of three amplifiers/loss components as given by

$$F_{\mathrm{tot}} = F_1 + \frac{F_2 - 1}{G_1} + \frac{F_3 - 1}{G_1 G_2} \quad (1)\,.$$

In our case, $F_1 = 1/L$ and $F_3 = 1/L$ are the noise factors due to the coupling loss L (decreasing SNR due to shot-noise) at the input and output, respectively [20]. F_2 is the isolated on-chip noise factor (calculated with NF equation 2 below, using on-chip power levels) of the amplifier (signal-spontaneous beat noise + the shot-noise component $1/G_2$ with the amplifier gain factor G_2), and $G_1 = L$ is the input coupling loss. All units are in linear scale meaning output power divided by input power. The measured coupling loss was $L = 0.60$ (-2.2 dB) and the lowest tested amplifier gain was $G_2 = 10$ (10 dB) with a corresponding on-chip noise factor of $F_2 = 5.4$ (7.3 dB). Using equation 1, the resulting fiber-to-fiber noise figure is $\mathrm{NF}_{\text{f-f}} = 9.55$ dB, which includes now the 2.2 dB coupling loss per facet and is 2.25 dB higher than the measured on-chip noise figure of 7.3 dB. Crucially, the output coupling loss only adds 0.05 dB to the total noise figure. The best measured on-chip noise figure of 3.6 dB at 29.8 dB gain corresponds to a fiber-to-fiber noise figure of 5.8 dB. In that case, the output coupling loss adds practically nothing to the noise figure, because $G_2 >> L$, such that the output coupling loss becomes negligible (third component in eq. 1). We see that even if the entire 25.4 dB loss of the fiber test setup on the output side were considered (meaning there is almost no fiber-to-OSA net gain) the noise figure would still only increase by another 0.6 dB, because still $G_2 > L$.

To verify these noise figure values, we also calculated directly the fiber-to-OSA noise figure with the output signal as measured on the OSA, which includes all 25.4 dB fiber component losses on the output side and also the input coupling loss. In that case, the total fiber-to-OSA net gain measured on the OSA was only $G_{\mathrm{tot}} = 2.15$ dB, which matches to the 29.8 dB on-chip gain with -25.4 dB output loss and -2.2 dB input coupling loss. The measured ASE power at the signal wavelength was $P_{\mathrm{ASE}} = 3.28$ nW in the equivalent noise bandwidth of the OSA $B_0 = 0.053$ nm = 4.81 GHz (at 1818 nm signal wavelength), and $G_{\mathrm{lin}} = 1.64$ (2.15 dB). Using the noise figure formula

$$NF = \frac{2\,P_{\mathrm{ASE}}}{G_{\mathrm{lin}}\, h\nu\, B_0} + \frac{1}{G_{\mathrm{lin}}} \quad (2)\,,$$

we calculate directly a fiber-to-OSA noise figure of 6.4 dB. Inverting Friis' formula and using $G_{\mathrm{tot}} = G_2 - 25.4$ dB – 2.2 dB (G_{tot} is the fiber-to-OSA gain, G_2 is the isolated on-chip gain) to obtain the isolated on-chip gain value, we obtain an on-chip noise figure of 3.6 dB, which is identical to the value reported in the main manuscript.

We also performed a simple test to confirm that additional losses after the amplifier do not significantly increase the noise figure, as long as the amplifier gain is higher than the losses, which is shown in Fig. S3. We believe this confirms that we calculate on-chip and off-chip NF values correctly. For this, we measured the noise figure of a 6-cm-long amplifier using a similar setup to the one described in the main manuscript, only that we now inserted additional losses directly before the OSA in the form of fiber attenuators with fixed losses. We did not use the chip and the amplifier devices presented in the main manuscript, because their performance has degraded from extensive tests in a non-clean room environment and excessive manual handling. For the chip that was tested here, the facets were not protected during gain film deposition, so the high-index Al_2O_3 film also covered the input and output facets, leading to an earlier onset of parasitic lasing. Additionally, the 10.7-cm long device was not used due to small fabrication defect in the gain film, which impacted the device performance slightly. Due to the early onset of lasing, we could only test the amplifier in low-gain operation with 11.4 dB on-chip net gain. The coupling losses were 2.6 dB per facet and the total losses after the amplifier (including output coupling loss, fiber components and the additional fiber attenuators) ranged from 10 to 29 dB. We measured the noise figure off-chip (fiber-to-OSA) by taking the ASE power and fiber-to-fiber gain directly from the OSA data without any power level adjustments (Red dots in Fig. S3). From this measured off-chip NF, we calculated the corresponding on-chip NF by inverting Friis' equation as above (+ symbols in Fig. S3). Then, for comparison, we reversed the procedure and extracted first the on-chip noise figure by adjusting the gain and the measured power levels on the OSA with the coupling and fiber losses (Green diamonds in Fig. S3). Subsequently we used the so-measured on-chip NF in Friis' formula again to calculate the corresponding off-chip NF including all input and output losses (x symbols in Fig. S3). As we expected from Friis' equation, both methods yield the same results, as it is only a matter of changing the power levels to the

relevant reference points in the system. We can also identify that the additional losses after the amplifier only become significant when they become similar or larger than the amplifier gain, which is around 10 dB in this case. In the case of 0 additional output losses, the on-chip and fiber-to-fiber noise figure differ by exactly the input coupling

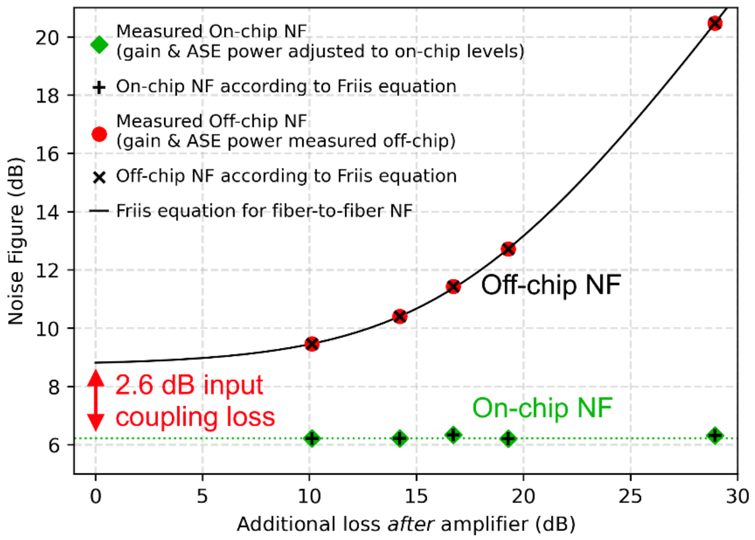

Fig. S3: Degradation of the noise figure due to additional losses at the amplifier output. Measured noise figure of a 6-cm-long amplifier according to different reference planes: On-chip and off-chip (fiber-to-fiber) power levels and comparison with corresponding values calculated with Friis' formula. The on-chip net gain of the amplifier was 11.4 dB.

loss.

4. Spectroscopic analysis of the gain film

An in-depth spectroscopic analysis of the thulium-doped gain film has been performed on the two amplifier devices. Important parameters for the design and simulation of the amplifiers were determined including the excited state lifetime, absorption and emission cross-sections and energy transfer upconversion parameters. An overview of the measurements and results are presented in the following.

4.1 Photoluminescence measurements and excited state lifetime

The excited state lifetime was extracted from the time response of the photoluminescence (PL) signal upon excitation with a short rectangular pump pulse. The pump light excites the thulium ions in the gain film of the amplifier device to the first excited state. The excited ions spontaneously transition back to the ground state with a characteristic transition rate, typically expressed through its inverse quantity, the excited state lifetime. Several mechanisms can lead to the de-excitation of a thulium ion, which are categorized into radiative decay and non-radiative decay mechanisms. The radiative decay rate is a material constant, which is dependent on the type of rare earth ion and the host matrix and is the result of de-excitation through the spontaneous emission of a photon in the characteristic emission bandwidth (PL emission). Non-radiative decay can mostly be divided into two components, multi-phonon relaxation and energy transfer upconversion (ETU). In the case of multi-phonon relaxation, the excited ion transfers its energy to multiple phonons or vibrational modes of the host material. Like the radiative decay rate, the multi-phonon decay rate is also a characteristic material property and depends on the specific host configuration [7,21]. The upconversion process, however, is a very different decay mechanism, which is depicted in Fig. S1d. Here an excited ion transfers its energy through dipole-dipole interactions to another excited ion [9]. Therefore, the first ion drops to the ground state while the second ion is promoted to an even higher excited state, which effectively reduces the excited state population by one. This process requires two nearby ions to be excited at the same time and therefore depends nonlinearly on the excited state population. Thus, the ETU-based decay is accelerated at high pump powers where the level of inversion is high. Furthermore, energy can be transferred from an excited ion to a ground state ion multiple times before reaching another excited ion, which is also referred to as energy hopping or excitation hopping and further facilitates the upconversion process [8]. PL measurements provide a powerful tool to extract the different excited state decay components as well as parameters to model the ETU process.

A schematic of the PL measurement setup is shown in Fig. S4a. 1.61 µm pump light is edge-coupled into the amplifier device via a lensed fiber. The pump light is chopped (Thorlabs MC2000B-EC with MC1F2P10 blade) at a 50 Hz chopping rate and 10% duty cycle (2 ms pump-on-time, 18 ms pump-off-time). The 2 ms pump pulse is long enough for the PL signal to reach a steady-state condition before the pump is blocked and the PL signal decays with the characteristic decay time. To avoid the effects of reabsorption and radiation trapping inside the waveguide, the PL is collected with an out-of-plane multimode fiber (400 µm core Thorlabs M124L02) from the top of the chip. The collected PL is collimated to a free-space beam, a bandpass filter (Thorlabs FB2000-500) is used to remove scattered pump light, and the signal is then detected with an amplified extended InGaAs detector (Thorlabs PDA10D2) and recorded on an 8-bit digital oscilloscope (Siglent SDS 1304CFL). Additionally, a 20 kHz electrical low-pass filter (Thorlabs EF122) is placed after the detector to filter out high-frequency noise from the PL signal. To reduce the statistical error, each PL signal trace was averaged 128 times on the oscilloscope and every measurement was repeated 10 times. An example of the PL time response is shown in Fig. S4b. To measure the PL spectrum, the out-of-plane PL collection fiber was connected directly to an optical spectrum analyzer (OSA, Yokogawa AQ6375E). No bandpass filter was employed in this case to measure the undistorted PL spectrum. A narrow peak from the scattered pump light in the spectrum was removed in post-processing. Moreover, there were sharp absorption lines between 1800 and 1950 nm, which were caused by humid air inside the OSA and were also removed in post-processing.

PL signals in spectral and time domain were measured at various pump powers ranging from 1.5 to 430 mW. As shown in Fig. S4c, the PL increases in power as the pump power is increased, while the spectral shape is independent of pump power. With more pump power, more thulium ions are excited and thus the number of ions contributing to PL emission also increases. A slow saturation can be observed in the integrated PL power as the pump power is increased, as shown in the inset of Fig. S4c. Due to the large mode area, very high pump power is required to achieve a high level of inversion, which is also the reason for the high gain saturation power and strong gain with high-power signals. As typical for thulium, the emission spectrum is very broad, spanning from ~1600 to ~2100 nm. As shown later in Fig. S5b, the absorption spectrum extends up to ~1800 nm, overlapping with the emission spectrum, which limits the effective gain spectrum from ~1800 to ~2000 nm.

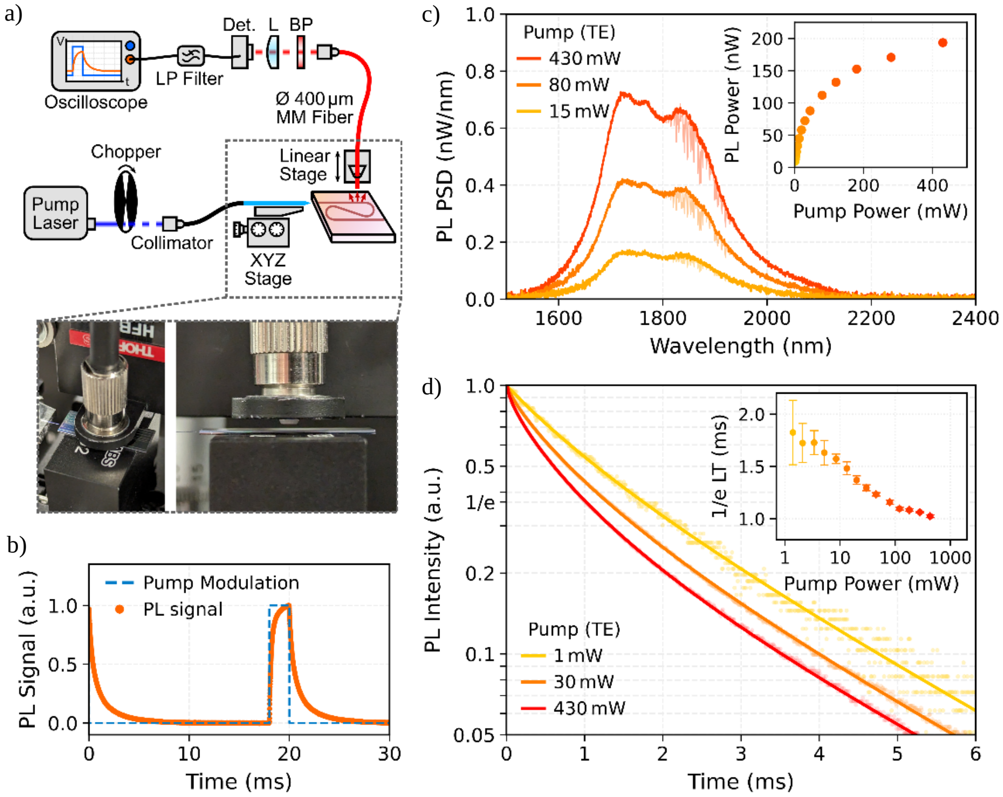

Fig. S4: Photoluminescence characterization of the gain medium. a) Schematic of the PL lifetime measurement setup. b) Measured PL time response (orange dots) and pump pulse signal (blue, dashed line). c) PL spectrum at three pump power levels. The fringes in the spectrum between 1800 and 1950 nm are caused by absorption due to humid air inside the OSA and were removed in post-processing. The inset shows the total power of the integrated PL spectrum as a function of pump power. d) Measured PL decay over time at three pump power levels (dots) and fits with an adaptation of Zubenko's model of ETU (solid lines). The data is normalized to 1 and displayed in semi-logarithmic scale to emphasize the change with different pump powers. The inset shows the 1/e lifetime as a function of pump power and errorbars indicate the standard deviation of the 1/e lifetime from 10 recorded decay curves at each power level.

The PL decay traces after pump pulse excitation are shown in Fig. S4d for three pump power levels. The data is shown in semi-logarithmic scale, and every trace is normalized to its maximum PL intensity to highlight the change at different pump powers. Additionally, an adapted version of Zubenko's model of energy transfer upconversion is fit to the data to extract the intrinsic excited state lifetime and several ETU parameters [8,9]. An initial fast decay of the PL signal can be observed when the pump power is increased, which shortens the effective decay time. This is also highlighted in the inset, where we plot the 1/e lifetime (the time it takes for the PL signal to drop to 1/e ≈ 0.37 of its initial intensity) as a function of pump power, dropping from 1.8 ms at low pump power to 1.0 ms at high pump power. This is the result of the energy transfer and upconversion processes that happen at high excitation densities resulting in the rapid deactivation of excited ions. After > 3 ms most of the ions have dropped to the ground state and the ETU process becomes less significant. In this regime, the PL decay is mostly the result of radiative and multi-phonon decay processes, which are both characteristic properties of the gain material and independent of the pump condition. Both can be combined into the intrinsic excited state lifetime $1/\tau_1 = 1/\tau_{rad} + 1/\tau_{non\text{-}rad}$. The fit with Zubenko's model provides the intrinsic excited state lifetime τ_1 = 2.85 ms, the microscopic energy migration parameter $C_{DD} = 5.1\times10^{-51}$ m^6/s (donor-donor energy transfer) and the microscopic ETU parameter $C_{DA} = 1.3\times10^{-52}$ m^6/s (donor-acceptor energy transfer). The two microscopic parameters can be combined with the Tm^{3+} concentration $N_d = 4.0\times10^{20}$ cm^{-3} into the macroscopic ETU parameter $W_{ETU} = 1.1\times10^{-24}$ m^3/s, which is used in the rate equations to simulate the amplifier performance (see supplementary section 6).

4.2 Intrinsic saturation power and transition cross-sections

Accurate amplifier and laser simulations require careful determination of the absorption and emission cross-sections of the thulium gain medium, as this determines how much gain can be expected at which wavelengths. We used a measurement approach based on the intrinsic saturation power of the amplifier device [22], because the saturation power is directly connected to the cross-sections through

$$P_{\text{sat}} = \frac{h\nu\, A_{\text{eff}}}{\Gamma\,(\sigma_{\text{abs}} + \sigma_{\text{em}})\,\tau_{1/e}} \quad (3)\,,$$

in which hν is the photon energy, A_{eff} is the effective mode area, Γ is the mode overlap factor with the gain layer, σ_{abs} and σ_{em} are the absorption and emission cross-section, and $\tau_{1/e}$ is the effective ETU-dependent excited state lifetime. The small-signal absorption coefficient $\alpha_{abs} = \Gamma N_d \sigma_{abs}$ is also included in the saturation power model. We chose this approach, as it reduces the number of unknown variables and estimations that would have been required with other measurement methods. For example, the Füchtbauer-Ladenburg relation is commonly used to calculate the emission cross-section from the PL spectrum, which requires knowledge of the radiative lifetime [23]. This lifetime value could not be extracted from the PL measurements, because the observed lifetime is always the combination of radiative and non-radiative lifetime. An isolated radiative lifetime value may be obtained from a Judd-Ofelt analysis [24], but this requires already known cross-section values of several other thulium transitions to higher excited states to perform a model fit, which was not feasible with our device, and is known to be somewhat inaccurate in predicting absolute peak cross-sections [25]. With the saturation power method, all parameters were either measured directly or calculated from mode simulations for the given waveguide structure. The intrinsic saturation power was extracted from the power-dependent transmission of a CW signal through a L = 6.5 cm long amplifier device. Subsequently, the transmission data was fit with the transcendental equation

$$\frac{P_{\text{out}}}{P_{\text{in}}} = \exp\left(-\,\alpha_{\text{abs}}L - \frac{(P_{\text{out}} - P_{\text{in}})}{P_{\text{sat}}} - \alpha_{\text{prop}}L\right) \quad (4)\,,$$

which is derived from a two-level amplifier model [22]. σ_{abs} and σ_{em} in α_{abs} and P_{sat} are used as individual fit parameters. The measurement was repeated with various signal wavelengths ranging from 1570 to 1610 nm (pump region) and 1855 to 1948 nm (signal region) to partially retrieve the shape of the absorption and emission spectra, and the transmission data is shown in Fig. S5a.

The saturating quantity in this measurement is the absorption loss. Initially, the input power is too low to excite a significant number of ions, and the transmission is given by the small-signal absorption loss α_{abs} and the propagation loss α_{prop}. As the input power is increased, the fraction of excited ions increases depending on the cross-section values at the wavelength of the input signal. This ultimately leads to a steady-state situation where the probability of signal absorption and stimulated emission are equal, i.e. $N_0\,\sigma_{abs} = N_1\,\sigma_{em}$, and the absorption loss saturates. Now the overall device loss is only given by the background propagation loss. This approach can also be used to directly extract the total propagation loss of an active device, which can be challenging otherwise. In this case, the propagation loss at the signal wavelength was 0.22 dB/cm and at the pump wavelength it was 0.40 dB/cm. It should

be noted that in the propagation loss here we summarize the combination of all passive loss components, including Al_2O_3 film loss, SiN waveguide loss, taper losses and bend losses.

The cross-section values extracted from the saturation power fits are shown in Fig. S5b, including error bars estimated from the uncertainties in the effective mode area simulation, measured effective excited state lifetime, and doping concentration. Finally, the measured emission spectrum and the absorption spectrum of the amplifier (measured with a low-power supercontinuum source) were scaled in amplitude to match the extracted fit values and obtain the complete spectrally resolved cross-sections. We note that there is a slight distortion when the PL intensity spectrum is transformed into the emission cross-section due to a change of the units from intensity to photon emission probability [23]. Lastly, the Füchtbauer-Ladenburg relation can be applied to calculate the radiative lifetime from the now measured absolute emission cross-section values, yielding τ_{rad} = 4.2 ms.

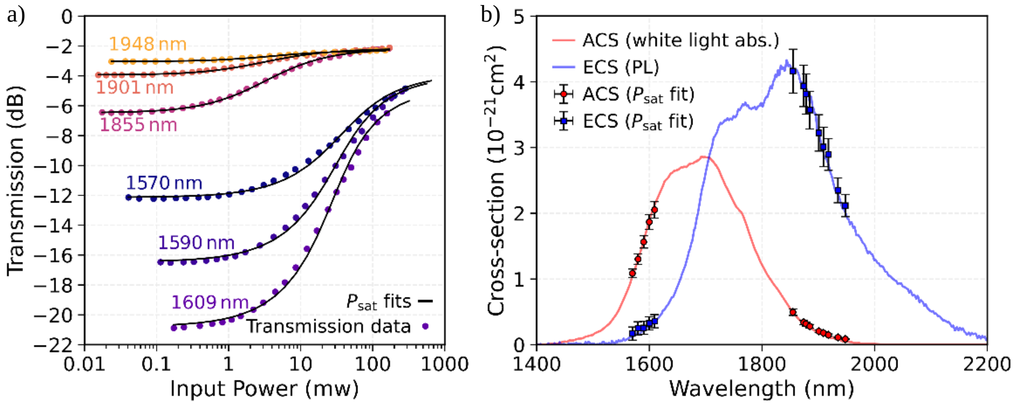

Fig. S5: Intrinsic saturation power of the amplifier and cross-sections of the gain material. a) Measured transmission of CW signals through a 6.5 cm amplifier device as a function of signal input power at various wavelengths (dots) and fits with the saturation power equation (solid lines). b) Absorption (ACS, red dots) and emission cross-section values (ECS, blue squares) extracted from the saturation power fits. The red solid line shows the amplifier absorption spectrum measured with a supercontinuum source and the blue solid line shows the measured PL spectrum, both adjusted in amplitude to match the fit values.

5. Tunable polarization sensitivity

5.1 Device design and measurement details

All PDG measurements were performed on the second amplifier device, which has a 1.1 µm thick gain layer and a protective SiO_2 top cladding deposited via PECVD, which introduces a small amount of propagation loss due to OH-bonds in the film. Post annealing of the amplifier chip at 600°C significantly reduced the absorption around 1.9 µm wavelength from the OH-bonds, but the background loss remained noticeably higher than in the chip without the protective top cladding. The thicker gain film and the top oxide cladding lead to increased mode areas for both TE and TM polarizations, with the TE mode area being 57 µm² and the TM mode area being 26 µm². The TE mode has slightly more overlap with the SiO2 top cladding than the TM mode and the probability of interacting with defects in the gain film is also higher due to the larger mode area, leading to a slightly higher propagation loss for TE polarized pump and signal light. The measurement setup was identical to the one shown before, with the pump and signal light coming from external sources and their polarizations were controlled via quarter- and half-wave plates.

Initially, the signal net gain was measured for all four combinations of TE and TM signal with TE and TM pump polarizations at various pump power levels. With the index-matching fluid (Luvantix SH-548HT) applied to the waveguide facets and lensed fibers, the coupling losses were 4.7 dB and 7.1 dB for the TE and TM polarized pump light, and 3.9 and 4.9 dB for the TE and TM polarized signal light, respectively. Three sets of measurements were performed: one at low signal power (~ 10 µW) and one at high signal power (~ 15 mW) and one at an intermediate power level (~ 1.5 mW) with varying pump powers and the results are shown in Fig. S6a–c with the corresponding PDG (G_{TE} – G_{TM}) for fully TE and fully TM polarized pump. Finally, two more sets of measurements were performed with mixed pump polarizations (~ 95% TE and ~ 70% TE) and the results are shown later in Fig. S7 as well as in the main manuscript. The first two sets of measurements helped to estimate the pump power and polarization states required for 0 dB PDG at various signal powers. The second set of measurements demonstrated that the PDG can be tuned via the pump polarization and highlight how the PDG changes with different signal powers.

5.2 PDG dependence on pump power and pump polarization

The gain dependence on the pump polarization is a result of two effects:

1) The saturation powers of the signal and pump modes are higher for TE polarized light due to the larger mode area ($P_{sat} \sim A_{eff}$). This means that in the case of TE polarized light, more pump power is required to reach a high level of inversion in the amplifier. But if the pump power is high enough, the TE signal gain is also stronger, because the larger mode supports higher power signals due to a slower saturation of the gain. This can be identified by comparing the TE signal gain curves (red curves) with the TM signal gain curves (blue curves) in Fig. S6a. The TM gain increases quickly but flattens out earlier than the TE signal gain, so that the TE gain will surpass it at higher pump powers. This effect is the result of the higher saturation power with the TE signal mode and is independent of the pump polarization or the signal power, meaning that in any case the TE signal will eventually be stronger than the TM signal if the pump power is high enough.

2) The gain depends on the spatial overlap of the pump and signal modes. Optimal gain can be achieved when both the signal and the pump light are in the same polarization, as then the pump and signal modes are very similar in size and the spatial overlap is high. The gain is lowest when the pump is TM polarized and the signal is TE polarized, as the TM pump mode area is smaller and therefore the TE signal light encounters more absorption loss.

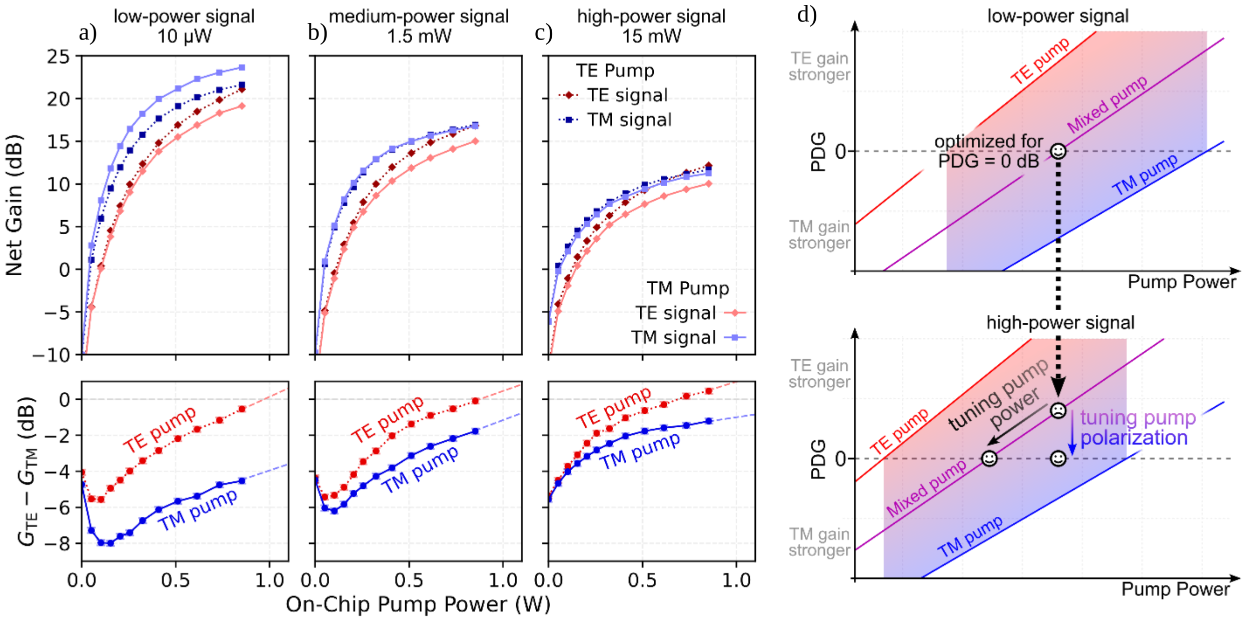

Fig. S6: Gain measurements with different pump and signal polarizations. Measured on-chip net gain as a function of on-chip pump power for all four combinations of TE and TM signal and pump polarizations for a) a low-power signal (10 µW), b) an intermediate signal power (1.5 mW) and c) a high-power signal (15 mW). Red and blue lines indicate TE and TM signal polarization, respectively. Dashed and solid line styles indicate TE and TM pump polarization. The lower plots show the corresponding polarization dependent gain (difference between TE and TM signal gain) when the pump is fully TE polarized (red) or fully TM polarized (blue). d) Sketch of the PDG for TE, TM and mixed pump polarization as extrapolated from the data in a) and b). The shaded area shows the pump parameter space in which the PDG can be tuned to 0 dB for the cases of low and high signal power. In the low-power case, slightly more pump power is required for the overall PDG tunability, which is highlighted by the shaded region shifting to the right compared to the low-power case. An optimized PDG for low-power signals will not generally be also optimized for high-power signals. But the PDG can be re-adjusted by tuning either the pump power or the pump polarization.

A certain level of pump power is required to achieve 0 dB PDG, as can be seen in the lower plots of Fig. S6a–c. In the case of low-power signals, that are well below the intrinsic saturation power ($P_{sat} \sim 10$ mW) as in Fig. S6a, the TE gain only becomes stronger than the TM gain around 1.05 W pump power on-chip. With high-power signals (power comparable to P_{sat}), the TE signal gain surpasses the TM gain already at 0.70 W pump power, as the gain saturation power is higher for the TE mode. The data shown in Fig. S6a–c indicate that the PDG can be tuned to 0 dB for any signal power by tuning the pump power or the pump polarization individually or both together. This principle is sketched in Fig. S6d. In this example, the PDG is initially optimized to 0 dB for low-power signals with a specific mixed pump polarization, here 50% TE and 50% TM. If the signal power is now increased significantly, the PDG will also increase, because high-power signals can extract more gain in TE polarization than in TM polarization. The PDG can now be optimized back to 0 dB by (a) keeping the pump power the same but tuning the pump polarization

more towards TM, or (b) keeping the polarization the same but decreasing the pump power. The advantage of tuning the pump polarization rather than the pump power is that the overall signal gain will only change marginally, while the gain might change more significantly when the pump power is tuned instead.

Alternatively, the pump settings can also be tuned to achieve high polarization dependence with the same device. As seen in Fig. S6a, the gain for low-power TM signals is much higher when the pump power is low and the pump polarization is fully TM. In this case, the PDG can be as high as 8 dB at 0.2 W pump power, with 12 dB gain for TM signals and only 4 dB gain for TE signals. If higher gain is required, the pump power may be increased, but this would lead to a reduction of the PDG. The device may also be specifically designed for a high PDG for example by reducing the gain film thickness similar to the first amplifier device. This leads to a much smaller TM mode that interacts strongly with the rough SiN waveguides below the gain layer, which drastically increases the propagation loss for TM polarized signals. In this case, only the TE signals can achieve high gain and the PDG can be > 10 dB.

5.3 PDG dependence on signal power

The second set of measurements demonstrated the PDG tunability over a wide range of signal powers. The results are shown in Fig. S7 and in the main manuscript. For the first measurement, the PDG was optimized to 0 dB at high signal powers (~ 10 mW) by setting the on-chip pump power to 0.85 W and tuning the pump polarization to a mix of approximately 70% TE and 30% TM. When the signal power is now reduced, the PDG starts to drop gradually until it settles at around – 0.9 dB for signal powers < 30 µW. For the second measurement, the PDG was optimized for medium signal powers (~ 0.5 mW) by keeping the pump power constant and only tuning the pump polarization to be almost completely TE (~ 95%). As seen in Fig. S7b, the shape of the PDG curve with respect to signal power is nearly identical but shifted up such that the 0 dB-point is now around the intermediate signal powers. In fact, a similar S-shaped curve can be observed from the power-dependent transmission data shown in Fig. S5a, indicating that the PDG behavior is a result of the different intrinsic saturation powers with TE and TM modes. Combining this with the data shown in Fig. S6 we estimate that the PDG curve at ~ 20% higher pump power and full TE pump polarization would be optimized for low-power signals. The high pump power was avoided in the measurements as the index-matching fluid already showed degradation due to prolonged high-power pumping and the device was at risk of being damaged.

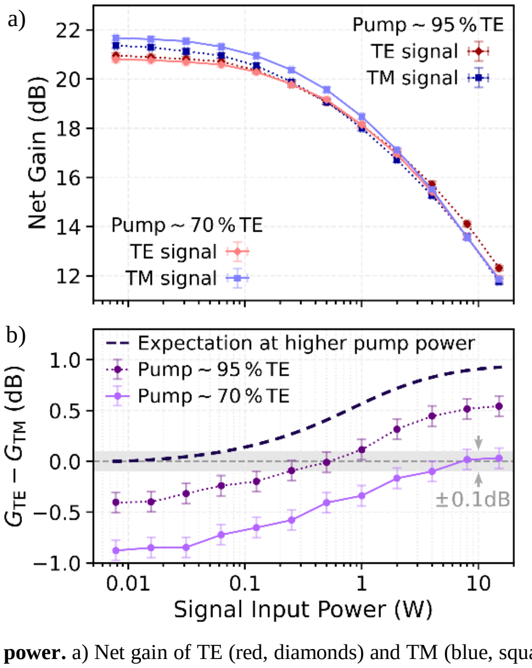

Fig. S7: PDG as a function of signal power. a) Net gain of TE (red, diamonds) and TM (blue, squares) signals as a function of signal input power with two different mixed pump polarizations (solid lines: 95% TE / 5% TM; dotted lines: 70% TE / 30 % TM). The pump power is kept fixed at 0.85 W on-chip. b) Difference between TE and TM signal gain corresponding to the data with mixed polarizations from a). The dashed line is an estimation of the gain difference with 100% TE pump polarization and approximately 20% more pump power, to achieve 0 dB gain difference at low signal power.

5.4 PDG with mixed input polarization

We have performed additional gain measurements with mixed input polarization to verify that the PDG can be determined from measuring the gain difference between TE and TM signal polarizations only. We have found that the cases where the signal is completely TE or TM polarized correspond to either the minimum or maximum gain extraction for any given pump condition. Any arbitrary signal polarization would fall somewhere between these two extremes and converge to the same zero-PDG point.

To test this, we measured the gain with clean TE and TM polarized input signals and also with an equal polarization mix of TE+TM. In the test setup, we rotated the linearly polarized input signal to be diagonal at a ~ 50° angle to the waveguide TE axis (slightly off from 45° to compensate for the 1.2 dB higher coupling loss of the TM component). That way, the signal light couples equally into the TE and TM polarized waveguide modes and both components have the same on-chip input power, which we have confirmed with a short passive reference waveguide. On the amplifier output side, we collimated the amplified signal with a lens to a free-space beam, to be able to determine the output polarization state. Having the signal output as a free-space beam allowed us to measure the power of each polarization component of the amplified signal individually by isolating it with a wire-grid polarizer. For every polarization orientation, we made sure that the same total signal input power of 0.5 mW is coupled to the waveguide. The pump polarization in this case was a 60/40 mix of TE and TM. The measured data is shown in Fig. S8a and our expectation of the gain behavior is schematically shown in Fig. S8b. The measurements were performed with a 6-cm-long amplifier on a different chip to the one shown in the manuscript, as that device has degraded over long periods of manual handling, as mentioned above in section 3.2. The achievable gain with the 6-cm-long device was limited by a relatively early onset of parasitic lasing at ~ 12 dB gain, because of a slightly different fabrication process where the chip facets were also coated with the gain film, leading to higher facet reflections due to the higher refractive index of Al_2O_3 (for the devices discussed in the manuscript, the chip facets were covered during the Al_2O_3deposition to avoid such a facet coating). Application of index-matching glue in this case did not reduce the facet reflections.

The results demonstrate that a signal with a mixed polarization state has the same zero-PDG point as fully TE or TM polarized signals. Moreover, at pump conditions that deviate from the zero-PDG point, the mixed signal gain (green dashed line) is exactly in-between the TE and TM gain curves (red and blue solid lines). We also noticed – when operating far away from the zero-PDG point – that the gain of the TE and TM components in the mixed polarization case (dotted red and blue lines) can have slightly stronger gain differences than when the signal is launched with a clean polarization. We expect that this happens, because the total input power in the mixed case is split into half TE and half TM, so that each component is of lower power and both are competing with each other for the gain. We have seen the same effect in the data shown in the main manuscript (Fig. 4), where the TM polarized signal was more strongly favored at low signal powers over the TE signal. Most importantly, however, the measurements confirm that every input polarization orientation converges to the same zero-PDG point, demonstrating that the zero-PDG condition does not require an alignment of the input polarization with the waveguide axis (i.e. that the input polarization would have to be in TE or TM orientation), and instead every

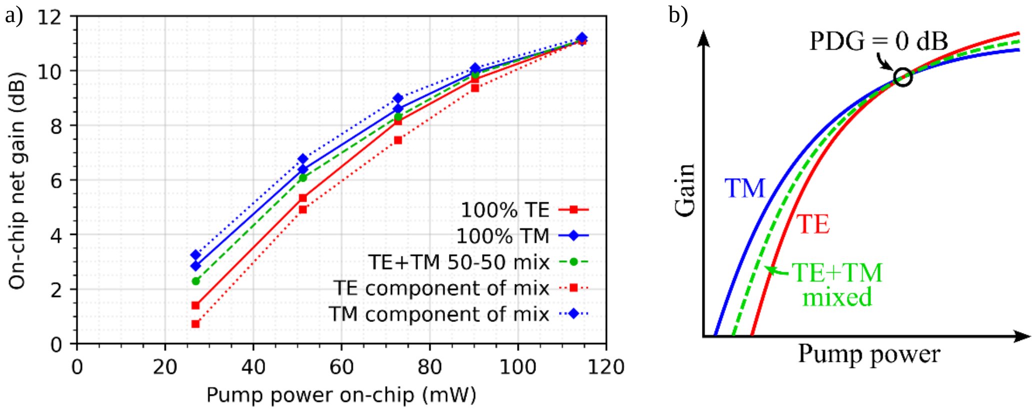

Fig. S8: Gain behaviour with mixed signal polarizations. a) Measured gain curves with a fully TE (red, solid line) and TM (blue, solid line) polarized input signal, a mix of 50% TE and 50% TM (diagonally polarized before coupling to the chip) with overall the same total input power (green, dashed line), and the measured gain of only the TE and TM components in the mixed case (red and blue, dotted lines). b) Concept drawing of the gain curves for TE and TM polarized signals and a signal with mixed polarization. Due to different waveguide and gain properties with the two polarizations (mode area, gain saturation power), the gain curves for every input polarization cross at a specific pump power, where the PDG = 0 dB.

polarization orientation gets amplified equally.

Furthermore, we confirmed that the amplifier waveguide maintains the TE and TM polarization through its entire length including the vertical SiN-to-Al_2O_3 transitions, the bends and the input and output couplers. This means that a completely TE-polarized signal at the input is still completely TE-polarized at the output without any measurable coupling or conversion to the other polarization. This polarization maintaining property does not generally hold for any arbitrarily mixed input polarization, as the input light then couples partially into the TE and partially into the TM waveguide modes, which propagate with slightly different group velocities (due to slightly different effective indices) and the polarization may be different at the output.

We have performed further measurements to verify that the same zero-PDG performance can also be achieved for off-chip (fiber-to-fiber) conditions. In this case, there is a difference in coupling loss of 1.2 dB per facet between TE and TM polarized components of an arbitrarily polarized input signal, favoring the coupling of TE-polarized light. Therefore, to compensate for the coupling loss difference, the amplifier must be operated at a point where the *on-chip* PDG is equal to the input and output coupling loss, i.e. the *on-chip* PDG has to be 2.4 dB. This would not be the case when the couplers are designed for equal coupling, which can be achieved easily with symmetric couplers (e.g. with a thinner SiN waveguide platform). To test the fiber-to-fiber zero-PDG performance, we recorded the amplified out-coupled power over time while manually adjusting the signal polarization with wave plates at the seed laser. We did not have a fiber-based polarization scrambler available for this test, so we chose to manually tune the polarization with wave plates. Before the test, we determined the wave plate positions corresponding to TE, TM and mixed TE+TM polarizations at the waveguide facet and cycled through these positions in 1-minute intervals. The data is shown in Fig. S9. The fiber-to-fiber PDG achieved in this test was ~ 0.05 – 0.10 dB, not exactly 0 dB, which can be mostly attributed to test setup instabilities, such as fluctuations of the fiber coupling stages over time, intensity fluctuations of the seed laser itself, and transmission irregularities of the wave plates at some orientations which caused small variations of the seed power (wave plate irregularities alone already contributed to ~ 1 % signal power variation between TE and TM orientation). Most of these instabilities would not be present in a properly packaged device. The pump conditions were tuned to the point where the measured fiber-to-fiber PDG was minimized. In this amplifier device, similar to the one presented in the main manuscript, the required on-chip PDG of 2.4 dB could only be achieved at low pump powers and relatively low gain (on-chip gain ~ 12 dB, fiber-to-fiber gain ~ 4 dB). Even though the measurement conditions for the fiber-to-fiber PDG were not perfect, we believe this test verifies the principle of zero-PDG for both on-chip and fiber-to-fiber conditions.

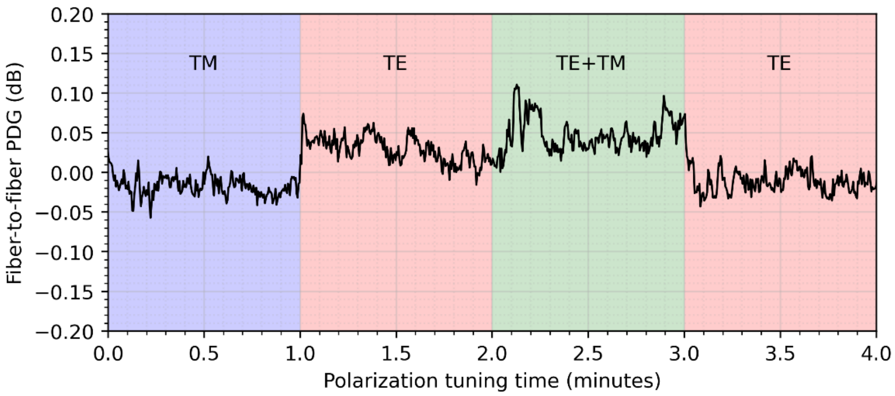

Fig. S9: Fiber-to-fiber PDG with actively changing signal polarization. The signal input polarization is changed in 1-minute intervals by manually tuning wave plates at the seed laser.

6. Gain simulations

A three-level amplifier model is used for the gain simulation including the effects of ETU and concentration quenching [9,10], given by the modified steady-state rate equations below:

$$0 = \frac{dN_{2,(\mathrm{a/q})}}{dt} = W_{\mathrm{ETU}} N_{1,(\mathrm{a/q})}^2 - \frac{1}{\tau_2} N_{2,(\mathrm{a/q})} \quad (5)$$

$$0 = \frac{dN_{1,(a/q)}}{dt} = R_{P,(a/q)} - R_{S,(a/q)} - 2\, W_{ETU} N_{1,(a/q)}^2 + \frac{1}{\tau_2} N_{2,(a/q)} - \frac{1}{\tau_{1,(a/q)}} N_{1,(a/q)} \quad (6)$$

$$f_{(\mathrm{a,q})} N_{\mathrm{d}} = N_{0,(\mathrm{a/q})} + N_{1,(\mathrm{a/q})} + N_{2,(\mathrm{a/q})} \quad (7)$$

$$R_{\mathrm{P},(\mathrm{a/q})} = \frac{\lambda_{\mathrm{P}}}{hc} I_{\mathrm{P}} \left(\sigma_{\mathrm{abs}}(\lambda_{\mathrm{P}}) N_{0,(\mathrm{a/q})} - \sigma_{\mathrm{em}}(\lambda_{\mathrm{P}}) N_{1,\mathrm{a/q}}\right) \quad (8)$$

$$R_{\mathrm{S},(\mathrm{a/q})} = \frac{\lambda_{\mathrm{S}}}{hc} I_{\mathrm{S}} \left(\sigma_{\mathrm{em}}(\lambda_{\mathrm{S}}) N_{1,(\mathrm{a/q})} - \sigma_{\mathrm{abs}}(\lambda_{\mathrm{S}}) N_{0,\mathrm{a/q}}\right) \quad (9)$$

The population densities are denoted by N_i, where i = 0, 1 or 2 represent the thulium ground state ($^3H_6$), first excited state ($^3F_4$), and the higher excited upconversion state ($^3H_4$), respectively. The index a or q denotes two different species of thulium ions, active and quenched. A small fraction of ions appears in pairs or small clusters inside the host matrix, which are referred to as quenched ions. These ions are so close to each other that there is an almost instantaneous energy transfer and upconversion between them. Therefore, only one of the paired ions can be excited at any time, which effectively reduces the number of ions available for amplification. For the simulation here, we use f_q = 1.5% quenched ions. The intrinsic excited state lifetime of the active ions was measured to be τ_{1a} = 2.85 ms, while the quenched lifetime is much shorter at τ_{1q} = 1 µs [9]. The lifetime of the higher excited state $^3H_4$ assumed to be similar to thulium-doped silica fibers with τ_2 = 20 µs [24]. R_P and R_S are the pump absorption and signal amplification rates, respectively, which are calculated from their intensities I_P and I_S, their wavelengths λ_P and λ_S, and the corresponding emission and absorption cross-sections σ_{em} and σ_{abs}. The ETU macroparameter was calculated to be W_{ETU} = 1.1×10^{-24} m^3/s from the microparameters C_{DA} and C_{DD} in the lifetime fit with Zubenko's model. The thulium doping concentration is N_d = 4.0×10^{20} cm^{-3}. The pump and signal power distribution along the amplifier propagation direction z can be calculated with the propagation equations

$$\frac{dP_{\mathrm{P}}(z)}{dz} = P_{\mathrm{P}}(z) \left\{ \iint \Psi_{\mathrm{P}} \left[\sigma_{\mathrm{em}}(\lambda_{\mathrm{P}}) N_{1,(\mathrm{a/q})} - \sigma_{\mathrm{abs}}(\lambda_{\mathrm{P}}) N_{0,(\mathrm{a/q})}\right] dxdy - \alpha_{\mathrm{prop}}(\lambda_{\mathrm{P}}) \right\} \quad (10)\,,$$

$$\frac{dP_{\mathrm{S}}(z)}{dz} = P_{\mathrm{S}}(z) \left\{ \iint \Psi_{\mathrm{S}} \left[\sigma_{\mathrm{em}}(\lambda_{\mathrm{S}}) N_{1,(\mathrm{a/q})} - \sigma_{\mathrm{abs}}(\lambda_{\mathrm{S}}) N_{0,(\mathrm{a/q})}\right] dxdy - \alpha_{\mathrm{prop}}(\lambda_{\mathrm{S}}) \right\} \quad (11)\,.$$

Here P_P and P_S are the pump and signal power, and Ψ_P and Ψ_S are the mode field distributions to calculate the effective mode area A_{eff} and the mode overlap factor Γ with the thulium-doped region. α_{prop} is the background propagation loss, which was estimated to be ~ 0.20 dB/cm at the signal wavelength and 0.40 dB/cm at the pump wavelength. All simulation parameters are also listed in table 1.

Table S1: Parameters for the amplifier simulations. [a] From [9]. [b] From [24].

**Parameter**	**Symbol**	**Value**
Pump wavelength	λ_p	1609 nm
Signal wavelength	λ_s	1833 – 1948 nm
Amplifier length (total)	L	10.7 cm
Amplifier length (active part)	L_{act}	9.85 cm
Propagation loss (pump)	$\alpha_{prop}(\lambda_p)$	0.40 dB/cm
Propagation loss (signal)	$\alpha_{prop}(\lambda_S)$	0.20 - 0.22 dB/cm (1833 – 1948 nm)
Effective mode area (pump)	$A_{eff}(\lambda_p)$	22.80 μm^2
Effective mode area (signal)	$A_{eff}(\lambda_s)$	21.38 – 21.25 μm^2 (1833 – 1948 nm)
Gain overlap factor (pump)	$\Gamma(\lambda_p)$	0.895
Gain overlap factor (signal)	$\Gamma(\lambda_s)$	0.863 – 0.841 (1833 – 1948 nm)
Thulium concentration	N_d	4.0×10^{20} cm^{-3}
Absorption cross-section	σ_{abs}	According to Fig. S5b
Emission cross-section	σ_{em}	According to Fig. S5b
$^3F_4$ intrinsic excited state lifetime	$\tau_{1,a}$	2.85 ms
$^3F_4$ quenched lifetime	$\tau_{1,q}$	1 μs [a]
$^3H_4$ intrinsic excited state lifetime	τ_2	20 μs [b]
ETU donor-donor microparameter	C_{DD}	5.1×10^{-51} m^6/s
ETU donor-acceptor microparameter	C_{DA}	1.3×10^{-52} m^6/s
ETU macroparameter	W_{ETU}	1.1×10^{-24} m^3/s
Fraction of quenched ions	f_q	0.015

7. Overview of various amplifier technologies

In the following table 2 we have compiled an overview of reported gain characteristics comparing different amplifier technologies of the last 3 decades, including rare earth-doped fiber amplifiers, silicon photonics-based amplifiers, and semiconductor optical amplifiers (both integrated on photonic platforms and non-integrated devices). Here we only highlight a few notable results from each type of amplifier technology. For a broader overview on silicon photonics-based amplifiers and on-chip amplifiers (Si- and SiN-based waveguide amplifiers and heterogeneously integrated III-V semiconductor amplifiers) we would like to refer to the supplementary material of reference [26], which was focused on devices operating in the C-band (1.55 µm erbium window). For an overview of erbium-doped waveguide amplifiers, including thin film lithium niobate (TFLN), various oxides (Al_2O_3, TeO_2, Ta_2O_5), silicate and polymer waveguides, we would like to refer to reference [27]. In the following table 2 we are comparing state-of-the-art amplifiers with a focus specifically on net gain, noise figure, PDG and PDG tunability, as well as output saturation power ($P_{sat,out}$). Although our LMA amplifier devices operate in the thulium wavelength window, we include here also mostly amplifiers operating in the erbium wavelength range (~ 1.55 µm), because the development of integrated amplifiers in the 2 µm wavelength range is severely limited. In the vast majority of chip-scale amplifiers, values for PDG are not reported because the devices are usually designed to operate in only one polarization mode and have an intrinsically very high PDG. Furthermore, output saturation powers are often not explicitly mentioned, but can sometimes be roughly estimated from the reported small-signal gain measurements. Lastly, we only list single amplifier architectures and exclude polarization diversity schemes (complex systems based on polarization beam splitters, polarization rotators and/or multiple amplifiers). We believe this overview highlights how much of an improvement in output power the LMA amplifier design provides, which we have shown in our previous works and again in this work. Furthermore, our amplifier device is comparable to or surpasses many commercial rare earth-doped fiber amplifiers in virtually every figure of merit. Meanwhile, semiconductor optical amplifiers (SOA) are able to provide very high output powers close to 1 W (e.g. slab-coupled optical waveguide amplifiers, SCOWA), however, achieving high output power, high gain and low noise figures simultaneously has been a big challenge. Furthermore, most SOAs have vastly different gain confinement factors for the TE and TM

polarized signal modes leading to a very high polarization dependence. Only in special designs can the PDG be reduced to less than 0.5 dB, but mostly at the cost of output power.

Table S2: Overview of key figures of merit of various optical amplifier technologies. TDFA/EDFA = thulium/erbium-doped fiber amplifier; EDFA = erbium-doped fiber amplifier; MQW = Multi-quantum-well; SCOWA = Slab-coupled optical waveguide amplifier; TDWA/EDWA = thulium/erbium-doped waveguide amplifier.

Ref.	Amplifier type	Max. net gain (dB)	Max. output power (mW)	Noise figure (dB)	Best PDG (dB)	Gain at best PDG (dB)	$P_{sat,out}$ (mW)	PDG tunability
**Rare earth-doped fiber amplifiers**								
[28]	TDFA	41	1200	~ 5	n.a.	n.a.	~ 250	n.a.
[29]	TDFA	40	140	3.1	n.a.	n.a.	~ 20	n.a.
[30]	Single EDFA	~ 40	~ 1	< 4.0	n.a.	n.a.	n.a.	n.a.
[30]	Dual-stage EDFA + isolator	54	~ 20	3.1	n.a.	n.a.	n.a.	n.a.
*a	Commercial EDFA	> 25	> 200	4.5	< 0.2	> 21	> 100	n.a.
**Various types of semiconductor optical amplifiers**								
[31]	Strained MQW	< 18	> 200	3.6	n.a.	n.a.	> 91	n.a.
[32]	SCOWA	< 15	~ 900	> 4.6	n.a.	n.a.	~ 800	n.a.
[33]	Quantum Dots	> 12	> 350	> 7	n.a.	n.a.	> 250	n.a.
[34]	Strained MQW	19	~ 90	7	~ 0.2	~ 18	50	No
[35]	Quantum Dots	~ 10	n.a.	n.a.	0.4	8	70	No
*b	Commercial packaged SOA	15	~ 30	8	1.0	15	~ 20	n.a.
*c	Commercial packaged BOA	28	~ 30	7	Very high	n.a.	~ 30	No (amplifies only single polarization)
**Photonic-integrated semiconductor optical amplifiers**								
[36]	III-V gain / Si (C-band)	27	56	~ 8	n.a.	n.a.	~ 30	n.a.
[37]	III-V gain / Si (C-band)	25	> 40	n.a.	n.a.	n.a.	42	n.a.
[38]	III-V gain / SiN (C-band)	14	~ 9	10.6	n.a.	n.a.	8	n.a.
[39]	III-V gain / Si (~ 2.0 µm)	13.1	18	17.2	n.a.	n.a.	n.a.	n.a.
**Silicon photonics-based rare earth-doped amplifiers**								
[10]	EDWA, etched Al_2O_3	20	~ 2.5	3.75	n.a.	n.a.	~ 0.4	n.a.
[40]	EDWA, etched Al_2O_3	33.5	21	n.a.	n.a.	n.a.	< 10	n.a.
[41]	EDWA, etched Al_2O_3 (packaged)	> 24.3	> 54	4.6	n.a.	n.a.	> 6	n.a.
[26]	EDWA, Er implanted SiN	30	145	~ 7	n.a.	n.a.	< 50	n.a.
[1]	LMA TDWA, power amplifier	16	1000	> 4.8	< 0.5	~ 10	n.a.	n.a.
[3]	LMA TDWA, power amplifier	13	1750	~ 5	n.a.	n.a.	n.a.	n.a.
**This work**	**LMA TDWA, high-gain operation**	**~ 30**	**> 800**	**3.6**	**0**	**> 21.5**	**> 115**	**Fully tunable over +1 to -1 dB**

Commercial amplifiers for reference:	
*a	Selection of commercial EDFAs:<br>• Thorlabs EDFA300S<br>• Thor F-PD-EDFA-25<br>• Nuphoton EDFA-CW-C4-HR-16-20-FCA<br>• bktel HPOA-1.5
*b	Thorlabs S7FC1013S (SOA – fiber coupled semiconductor optical amplifier)
*c	Thorlabs S9FC1004P (BOA – fiber coupled booster optical amplifier, only single polarization operation)

References

1. N. Singh, J. Lorenzen, K. Wang, M. A. Gaafar, M. Sinobad, H. Francis, M. Edelmann, M. Geiselmann, T. Herr, S. M. Garcia-Blanco, and F. X. Kärtner, "Watt-class silicon photonics-based optical high-power amplifier," Nat. Photonics **19**, 307–314 (2025).
2. K. Wang, C. O. Martinez, J. Lorenzen, M. Dijkstra, B. Jongebloed, N. Singh, F. X. Kärtner, and S. M. Garcia-Blanco, "Thulium-Doped Al_2 O_3 Waveguide Amplifiers Fabricated via Radio Frequency Reactive Co-Sputtering," in *2025 25th Anniversary International Conference on Transparent Optical Networks (ICTON)* (IEEE, 2025), pp. 1–4.
3. N. Singh, J. Lorenzen, M. Kilinc, K. Wang, M. Sinobad, H. Francis, J. Carreira, M. Geiselmann, U. Demirbas, M. Pergament, S. M. Garcia-Blanco, and F. X. Kärtner, "Sub-2W tunable laser based on silicon photonics power amplifier," Light Sci. Appl. **14**, 18 (2025).
4. A. Sincore, J. D. Bradford, J. Cook, L. Shah, and M. C. Richardson, "High Average Power Thulium-Doped Silica Fiber Lasers: Review of Systems and Concepts," IEEE J. Sel. Top. Quantum Electron. **24**, 1–8 (2018).
5. Th. Förster, "Zwischenmolekulare Energiewanderung und Fluoreszenz," Ann. Phys. **437**, 55–75 (1948).
6. D. L. Dexter, "A Theory of Sensitized Luminescence in Solids," J. Chem. Phys. **21**, 836–850 (1953).
7. J. Cajzl, P. Peterka, M. Kowalczyk, J. Tarka, G. Sobon, J. Sotor, J. Aubrecht, P. Honzátko, and I. Kašík, "Thulium-Doped Silica Fibers with Enhanced Fluorescence Lifetime and Their Application in Ultrafast Fiber Lasers," Fibers **6**, 66 (2018).
8. D. A. Zubenko, M. A. Noginov, V. A. Smirnov, and I. A. Shcherbakov, "Upconversion kinetics in hopping and other energy transfer regimes," in *Advanced Solid State Lasers* (OSA, 1997), p. SC19.
9. L. Agazzi, K. Wörhoff, and M. Pollnau, "Energy-Transfer-Upconversion Models, Their Applicability and Breakdown in the Presence of Spectroscopically Distinct Ion Classes: A Case Study in Amorphous Al_2O_3 :Er^{3+}," J. Phys. Chem. C **117**, 6759–6776 (2013).
10. S. A. Vázquez-Córdova, M. Dijkstra, E. H. Bernhardi, F. Ay, K. Wörhoff, J. L. Herek, S. M. García-Blanco, and M. Pollnau, "Erbium-doped spiral amplifiers with 20 dB of net gain on silicon," Opt. Express **22**, 25993 (2014).
11. B.-C. Hwang, S. Jiang, T. Luo, J. Watson, G. Sorbello, and N. Peyghambarian, "Cooperative upconversion and energy transfer of new high Er3Ƨ- and Yb3Ƨ– Er3Ƨ-doped phosphate glasses," (n.d.).
12. K. Kuroda, M. Nakandakari, and Y. Yoshikuni, "Pump–probe measurement of metastable state lifetime reduced by cooperative upconversion in a high-concentration erbium-doped fiber," Appl. Opt. **57**, 8819 (2018).
13. G. Nykolak, P. C. Becker, J. Shmulovich, Y. H. Wong, D. J. DiGiovanni, and A. J. Bruce, "Concentration-dependent /sup 4/I/sub 13/2/ lifetimes in Er/sup 3+/-doped fibers and Er/sup 3+/-doped planar waveguides," IEEE Photonics Technol. Lett. **5**, 1014–1016 (1993).
14. M. Kamrádek, J. Aubrecht, P. Vařák, J. Cajzl, V. Kubeček, P. Honzátko, I. Kašík, and P. Peterka, "Energy transfer coefficients in thulium-doped silica fibers," Opt. Mater. Express **11**, 1805 (2021).
15. U. Demirbas, J. Thesinga, E. Beyatli, M. Kellert, M. Pergament, and F. X. Kärtner, "Continuous-wave Tm:YLF laser with ultrabroad tuning (1772-2145 nm)," Opt. Express **30**, 41219 (2022).

16. J. D. B. Bradley, L. Agazzi, D. Geskus, F. Ay, K. Wörhoff, and M. Pollnau, "Gain bandwidth of 80 nm and 2 dB/cm peak gain in Al_2O_3 :Er^{3+} optical amplifiers on silicon," J. Opt. Soc. Am. B **27**, 187 (2010).
17. P. M. Becker, A. A. Olsson, and J. R. Simpson, *Erbium-Doped Fiber Amplifiers: Fundamentals and Technology* (Elsevier, 1999).
18. D. M. Baney, P. Gallion, and R. S. Tucker, "Theory and Measurement Techniques for the Noise Figure of Optical Amplifiers," Opt. Fiber Technol. **6**, 122–154 (2000).
19. S. D. Agger and J. H. Povlsen, "Emission and absorption cross section of thulium doped silica fibers," Opt. Express **14**, 50 (2006).
20. D. M. Baney, P. Gallion, and R. S. Tucker, "Theory and Measurement Techniques for the Noise Figure of Optical Amplifiers," Opt. Fiber Technol. **6**, 122–154 (2000).
21. B. Faure, W. Blanc, B. Dussardier, and G. Monnom, "Improvement of the Tm3+:3H4 level lifetime in silica optical fibers by lowering the local phonon energy," (2007).
22. A. A. M. Saleh, R. M. Jopson, J. D. Evankow, and J. Aspell, "Modeling of gain in erbium-doped fiber amplifiers," IEEE Photonics Technol. Lett. **2**, 714–717 (1990).
23. B. Aull and H. Jenssen, "Vibronic interactions in Nd:YAG resulting in nonreciprocity of absorption and stimulated emission cross sections," IEEE J. Quantum Electron. **18**, 925–930 (1982).
24. B. M. Walsh and N. P. Barnes, "Comparison of Tm : ZBLAN and Tm : silica fiber lasers; Spectroscopy and tunable pulsed laser operation around 1.9 μm," Appl. Phys. B **78**, 325–333 (2004).
25. E. Desurvire and M. N. Zervas, "*Erbium-Doped Fiber Amplifiers: Principles and Applications*," Phys. Today **48**, 56–58 (1995).
26. Y. Liu, Z. Qiu, X. Ji, A. Lukashchuk, J. He, J. Riemensberger, M. Hafermann, R. N. Wang, J. Liu, C. Ronning, and T. J. Kippenberg, "A photonic integrated circuit–based erbium-doped amplifier," Science **376**, 1309–1313 (2022).
27. X. He, Z. Zhang, D. Ma, C. Zhou, H. Hou, Y. Shuai, J. Liu, R. Wang, Z. Zhou, and W. Chen, "Erbium-doped/erbium-ytterbium co-doped waveguide amplifiers in silicon-based optoelectronics: recent progress," Adv. Photonics **7**, (2025).
28. Z. Li, A. M. Heidt, J. M. O. Daniel, Y. Jung, S. U. Alam, and D. J. Richardson, "Thulium-doped fiber amplifier for optical communications at 2 µm," Opt. Express **21**, 9289 (2013).
29. C. Romano, R. E. Tench, and J.-M. Delavaux, "Simulation of 2µm single clad thulium-doped silica fiber amplifiers by characterization of the$^3F_4 – ^3H_6$ transition," Opt. Express **26**, 26080 (2018).
30. R. I. Laming, M. N. Zervas, and D. N. Payne, "Erbium-doped fiber amplifier with 54 dB gain and 3.1 dB noise figures," IEEE Photonics Technol. Lett. **4**, 1345–1347 (1992).
31. K. Morito, S. Tanaka, S. Tomabechi, and A. Kuramata, "A broad-band MQW semiconductor optical amplifier with high saturation output power and low noise figure," IEEE Photonics Technol. Lett. **17**, 974–976 (2005).
32. P. W. Juodawlkis, J. J. Plant, W. Loh, L. J. Missaggia, F. J. O'Donnell, D. C. Oakley, A. Napoleone, J. Klamkin, J. T. Gopinath, D. J. Ripin, S. Gee, P. J. Delfyett, and J. P. Donnelly, "High-Power, Low-Noise 1.5-μm Slab-Coupled Optical Waveguide (SCOW) Emitters: Physics, Devices, and Applications," IEEE J. Sel. Top. Quantum Electron. **17**, 1698–1714 (2011).
33. T. Akiyama, M. Ekawa, M. Sugawara, K. Kawaguchi, Hisao Sudo, A. Kuramata, H. Ebe, and Y. Arakawa, "An ultrawide-band semiconductor optical amplifier having an extremely high penalty-free output power of 23 dBm achieved with quantum dots," IEEE Photonics Technol. Lett. **17**, 1614–1616 (2005).
34. K. Morito, M. Ekawa, T. Watanabe, and Y. Kotaki, "High-output-power polarization-insensitive semiconductor optical amplifier," J. Light. Technol. **21**, 176–181 (2003).
35. N. Yasuoka, H. Ebe, K. Kawaguchi, M. Ekawa, S. Sekiguchi, K. Morito, O. Wada, M. Sugawara, and Y. Arakawa, "Polarization-Insensitive Quantum Dot Semiconductor Optical Amplifiers Using Strain-Controlled Columnar Quantum Dots," J. Light. Technol. **30**, 68–75 (2012).
36. K. Van Gasse, R. Wang, and G. Roelkens, "27 dB gain III–V-on-silicon semiconductor optical amplifier with > 17 dBm output power," Opt. Express **27**, 293 (2019).
37. M. L. Davenport, S. Skendzic, N. Volet, J. C. Hulme, M. J. R. Heck, and J. E. Bowers, "Heterogeneous Silicon/III–V Semiconductor Optical Amplifiers," IEEE J. Sel. Top. Quantum Electron. **22**, 78–88 (2016).
38. C. Op De Beeck, B. Haq, L. Elsinger, A. Gocalinska, E. Pelucchi, B. Corbett, G. Roelkens, and B. Kuyken, "Heterogeneous III-V on silicon nitride amplifiers and lasers via microtransfer printing," Optica **7**, 386 (2020).

39. N. Volet, A. Spott, E. J. Stanton, M. L. Davenport, L. Chang, J. D. Peters, T. C. Briles, I. Vurgaftman, J. R. Meyer, and J. E. Bowers, "Semiconductor optical amplifiers at 2.0-µm wavelength on silicon," Laser Photonics Rev. **11**, 1600165 (2017).
40. D. B. Bonneville, C. E. Osornio-Martinez, M. Dijkstra, and S. M. García-Blanco, "High on-chip gain spiral Al_2O_3 :Er^{3+} waveguide amplifiers," Opt. Express **32**, 15527 (2024).
41. C. E. Osornio-Martinez, D. B. Bonneville, M. Dijkstra, A. R. Do Nascimento Jr., and S. M. García-Blanco, "Broadband Packaged Erbium-Doped Polycrystalline Al_2 O_3 Waveguide Amplifier with 24 dB External Net Gain," Opt. Express **33**, 28985 (2025).
