# Supplementary material for: Zero-PDG silicon photonic amplifier with high saturation power and low noise figure
Source: Nat Commun. 2026 Jun 30;17:5670. doi: 10.1038/s41467-026-74486-y (PMC13319230; doi:10.1038/s41467-026-74486-y)
Supplement: Supplementary file 2 — Transparent Peer Review file [file 41467_2026_74486_MOESM2_ESM.pdf]

nature portfolio

Peer Review File

Zero-PDG silicon photonic amplifier with high saturation power and low noise figure

Corresponding Author: Dr Neetesh Singh

This file contains all reviewer reports in order by version, followed by all author rebuttals in order by version.

Attachments originally included by the reviewers as part of their assessment can be found at the end of this file.

Version 0:

Reviewer comments:

Reviewer #1

(Remarks to the Author)
This manuscript presents the development of thulium-doped large mode area (LMA) integrated photonic amplifiers demonstrating 30 dB gain, >115 mW saturation power, and tunable polarization-dependent gain (PDG) control. While the technical execution is competent and the performance metrics are respectable, the work suffers from insufficient novelty and represents primarily incremental improvements over existing technologies rather than the breakthrough advances expected for publication in Nature Communications.
The fundamental approaches employed in this work are well-established in the integrated photonics community. Large mode area waveguide designs have been extensively developed and implemented in both fiber and integrated platforms for power scaling applications. The concept of rare-earth doping in silicon photonic platforms using RF sputtering techniques has been demonstrated multiple times, notably in the seminal Nature Communications work by Rönn et al. (2019) which achieved 20.1 dB/cm gain in erbium-based systems. The current manuscript essentially applies these existing methodologies to thulium-doped systems without introducing fundamentally new design principles or fabrication approaches. The LMA waveguide architecture follows conventional scaling laws and design rules that have been established for in high-power integrated photonics. For example, Singh et al. recently published in Nature Photonics (2024) demonstrating "Watt-class silicon photonics-based optical high-power amplifier" with similar LMA approaches.
The performance improvements demonstrated here, while technically sound, represent evolutionary rather than revolutionary advances. The 30 dB total gain, when normalized by device length, does not dramatically exceed the gain per unit length achieved in previous integrated rare-earth amplifiers. The >115 mW saturation power, though notable, falls within the expected scaling behavior when moving to larger mode areas and represents a predictable engineering outcome rather than a breakthrough. The work by Singh et al. on watt-class silicon photonic amplifiers has already demonstrated power handling capabilities that likely exceed or match the current work's achievements. The authors have not adequately positioned their results relative to this very recent and highly relevant benchmark, raising questions about whether their performance claims represent meaningful advances over the current state-of-the-art.
While the tunable PDG control is the most novel aspect of this work, it represents a relatively narrow technical capability that lacks the broad impact and fundamental significance expected for Nature Communications. The mechanism for PDG tuning, while useful for specific applications, does not reveal new physics or provide fundamental insights into light-matter interactions in integrated platforms. The capability appears to be primarily an engineering solution to a specific problem rather than opening new research directions or enabling transformative applications. Furthermore, the practical utility of precise PDG control in real-world systems is not convincingly demonstrated through system-level experiments or applications.
Nature Communications publishes work that significantly advances our understanding or capabilities in ways that impact multiple research areas and applications. This manuscript, while technically competent, addresses a relatively narrow set of performance parameters within the specific domain of integrated thulium amplifiers. The work does not provide new fundamental insights into rare-earth physics in integrated platforms, novel approaches to light confinement and amplification, or breakthrough system architectures that would influence the broader photonics community.
I recommend that the authors consider submission to specialized optics journals such as OPTICA or IEEE journals, where incremental performance improvements and specialized technical capabilities are more appropriately valued. To potentially reach higher-impact venues in the future, the authors should focus on demonstrating transformative applications, providing deeper fundamental insights into the underlying physics, or achieving performance breakthroughs that clearly exceed

existing benchmarks by substantial margins rather than modest improvements.

Reviewer #2

(Remarks to the Author)
The authors demonstrate a single integrated LMA amplifier capable of achieving both high-power amplification with output saturation powers >115 mW, high small-signal net gain up to 30 dB, and low-noise amplification with noise figures of ~3.6 dB. This concept is novel and the work could be considered for publication in Nature Communications after addressing the following concerns.

1. While high-power amplification over 1818-1948 nm is shown, and emission wavelength up to 2.1 µm is mentioned, a plot of the small-signal gain spectrum across the full potential bandwidth (e.g., 1700-2100 nm) would be very valuable to assess the true broadband potential.

2. The manuscript mentions PDG robustness after tuning but doesn't explicitly show data demonstrating stability over time or against environmental fluctuations (e.g., temperature). Including such data would strengthen the claim of practical polarization insensitivity.

3. The authors describe two approaches to achieve zero PDG: tuning the pump power or adjusting the pump polarization state. While on-chip polarization tuning presents challenges, they propose using a bidirectional pump scheme with two orthogonally polarized diodes as a solution. However, a key consideration arises: how would the significant Tm^{3+} ion absorption affects pump power uniformity along the 10 cm-long waveguide in this configuration?

4. The fabrication process of the device should be provided.

5. Comparing the net gain performance and other key figures of merit of the proposed devices with previously reported works would strengthen the analysis.

Version 1:

Reviewer comments:

Reviewer #1

(Remarks to the Author)

Reviewer #2

(Remarks to the Author)
The authors have fully addressed my questions and now the manuscript is ready to publish.

Reviewer #3

(Remarks to the Author)
The authors have demonstrated integrated waveguide amplifiers based on a dual layer waveguide platform with a silicon nitride waveguide layer (with high confinement) and an Al_2O_3 large mode area waveguide layer. The amplification occurs in the Al_2O_3 layer.

The main impact of this work is in demonstrating amplifiers with high output power and high gain, and simultaneously, with the interesting property that, under some conditions, they can be “tuned” to a condition of zero polarization dependent gain (i.e. PDG = 0 dB). This is a substantial advancement compared to previous works on the same topic, especially because of the addition of the PDG = 0 dB feature which, while not perfect, can be further improved and hopefully optimized in future works. Based on these considerations, I think that this work deserve to be considered for publication on a high impact journal such as Nature Communications.

Furthermore, with respect to the comments made by Reviewer # 1, I don’t agree with his/her comments. Reviewer # 1 objections are based on his/her opinion that this work “represents primarily incremental improvements”, that “suffers from insufficient novelty”, and that essentially is not a breakthrough. In contrast, in my view, demonstrating an amplifier in integrated photonics with high power, high gain, low noise figure, PDG = 0 dB (granted, only in some conditions), all at the same time, is a significant advancement and deserves to be considered for publication on a high impact journal.

This said, I have the following comments/questions for the authors:

- In regards to the measurements of polarization dependent gain (PDG) of Fig. 4 (Section 3.3):
(a) Could the authors please confirm that when they state that the polarization of the signal is TE (for example, red markers in Fig. 4b) what they mean is that the light injected into the silicon nitride waveguide at the chip input facet is TE polarized? Conversely, if this is not the case, and if instead what they mean by “TE signal” is that the light is TE polarized only once it is coupled to the Al_2O_3 layer, then I wouldn’t be sure that stating this amplifier has PDG = 0 dB is correct.

(b) Could the authors please confirm that TE-polarized light in the silicon nitride waveguide is coupled to the TE-polarized mode in the Al2O3 waveguide (and that TM-polarized light in the silicon nitride waveguide couples to the TM mode in the Al2O3 layer)?
(c) Specifically, in reference to the pump power tuning of Fig. 4(b) to search for the pump value that leads to PDG = 0 dB, I think it is a downside of this approach that there is only one specific value of pump that provides PDG = 0 dB for every signal power level.
I understand that the authors may be suggesting that the solution to this problem is to tune the pump polarization as shown in Figs. 4(c-d), but as hinted by the authors as well it is rather impractical to implement this pump tuning approach in a realistic system.
(d) One aspect that makes the authors' approach impractical is that it seems to me that the signals at the amplifier input must align with the waveguide axis (i.e. they must be TE and/or TM), otherwise for a differently polarized signal it would be even harder to find a PDG = 0 dB condition.
(e) I think that the best way to validate the authors' approach to achieve PDG = 0 dB would be to send two signals with orthogonal polarizations (TE and TM) at the waveguide input and check the gain experienced by the two signals. Has this test been performed?

- The authors report power levels and gain of the signals referred to the "on-chip" condition, i.e. they de-embed the coupling loss from the fiber to the chip:
(a)This approach is rather confusing because unless the amplifier is integrated with something else, there is an input fiber and an output fiber. Hence, please report also the "off-chip" values for both signal power levels and gain, and also pump power levels.
(b) Also, what is the fiber to chip coupling loss for the signals and the pumps at their respective wavelengths?

- In the evaluation of the noise figure, have the authors de-embedded the fiber to chip coupling loss from the NF values provided or not? In other words, are the numbers reported in the NF section (3.2) referred to "on-chip" conditions? Once again, if this is the case, it is confusing as you have an input fiber and output fiber, and if this is the case please report the NF values from fiber to fiber.

- The authors call this amplifier a "silicon photonic amplifier", however there are no silicon photonic components or silicon waveguides in this work. The amplifier is made by a dual layer waveguide technology incorporating a silicon nitride waveguide layer and an Al2O3 layer where the amplification occurs. There is no silicon photonic waveguide here, so please do not call this a silicon photonic amplifier. This is an amplifier for silicon nitride waveguides. In my opinion, it can be called a silicon photonics amplifier only if integration with a silicon waveguide is shown.

- The authors state in a few points in the text that SOAs are polarization sensitive. However, today there are several vendors that have commercialized and sell polarization independent SOAs (i.e. where the SOA waveguide is actually polarization insensitive, without using a polarization diversity scheme). Please correct this.

- The authors state that for integrated amplifiers the saturation power is limited to few mW. This is incorrect, in ref. 48 the output power is 145 mW. Also, in the paper "Broadband Packaged Erbium-Doped Polycrystalline Al2O3 Waveguide Amplifier with 24 dB External Net Gain") the output power was 17 dBm. Please correct this.

Version 2:

Reviewer comments:

Reviewer #1

(Remarks to the Author)
Thanks for the authors' response and revisions. I have only a few minor points we'd like to see addressed before the paper is ready for final acceptance.
1.It would help readers to understand, early in the paper, why you can't simply take an amplifier that was designed for one regime (pre-amp or power amp) and expect it to perform well in the other. The introduction or results section feels like the right place for this, and it would give the LMA architecture a clearer motivation. Right now, a reader unfamiliar with the tradeoffs might not fully appreciate what problem is being solved.
2.The 30 dB gain result needs a bit more framing in both the abstract and the main text. As it stands, it could be read as a deployment-ready figure, when in fact it relies on index-matching fluid to suppress facet reflections. That's a perfectly reasonable proof-of-concept approach, but it should be stated plainly. It would also be worth noting that anti-reflection coatings are the practical path forward and are expected to recover comparable performance in a more realistic configuration.
3.The noise figure discussion focuses on the on-chip value of 3.6 dB, but many readers will naturally think in terms of fiber-to-fiber performance. Given that you're looking at roughly 2.2 dB of coupling loss per facet, it's worth walking through what that means for the fiber-to-fiber NF explicitly, and then making the case that improved couplers could bring the fiber-to-fiber figure below 4.0 dB.
4.Given that this work builds on the authors' prior publication on the same platform, a brief statement clarifying what is actually new here would be useful.

Reviewer #3

(Remarks to the Author)
The authors have responded to my questions and have modified the manuscript accordingly. I do not have additional comments or questions. In my view, the manuscript can now be published.

Version 3:

Reviewer comments:

Reviewer #1

(Remarks to the Author)
The authors have addressed all reviewer concerns and the manuscript is ready for publication.

Open Access This Peer Review File is licensed under a Creative Commons Attribution 4.0 International License, which permits use, sharing, adaptation, distribution and reproduction in any medium or format, as long as you give appropriate credit to the original author(s) and the source, provide a link to the Creative Commons license, and indicate if changes were made.
In cases where reviewers are anonymous, credit should be given to 'Anonymous Referee' and the source.
The images or other third party material in this Peer Review File are included in the article’s Creative Commons license, unless indicated otherwise in a credit line to the material. If material is not included in the article’s Creative Commons license and your intended use is not permitted by statutory regulation or exceeds the permitted use, you will need to obtain permission directly from the copyright holder.
To view a copy of this license, visit https://creativecommons.org/licenses/by/4.0/

REVIEWER COMMENTS

We would like to thank the reviewers for taking their time out to evaluate our work and give valuable comments which greatly helped to improve our paper. Please find our detailed response to the comments. In the revised manuscript the text is colored to highlight the changes.

Reviewer #1 (Remarks to the Author):

This manuscript presents the development of thulium-doped large mode area (LMA) integrated photonic amplifiers demonstrating 30 dB gain, >115 mW saturation power, and tunable polarization-dependent gain (PDG) control. While the technical execution is competent and the performance metrics are respectable, the work suffers from insufficient novelty and represents primarily incremental improvements over existing technologies rather than the breakthrough advances expected for publication in Nature Communications.

The fundamental approaches employed in this work are well-established in the integrated photonics community. Large mode area waveguide designs have been extensively developed and implemented in both fiber and integrated platforms for power scaling applications. The concept of rare-earth doping in silicon photonic platforms using RF sputtering techniques has been demonstrated multiple times, notably in the seminal Nature Communications work by Rönn et al. (2019) which achieved 20.1 dB/cm gain in erbium-based systems. The current manuscript essentially applies these existing methodologies to thulium-doped systems without introducing fundamentally new design principles or fabrication approaches.

That is correct. We are using RF sputtering-based film deposition on our amplifier chips, which is unlike the ALD approach used in the work published in Nature Communications by Rönn et al., 2019 [1]. In that work, the authors achieved a net gain of **only 1.98 dB** in a very short 1.2-mm-long device and incorrectly extrapolated it to 20 dB/cm gain coefficient (see the table below taken from the paper). Such a value suggests that 20 dB gain could be achieved with a 1-cm-long device; however, this has never been actually demonstrated with longer devices. In fact, in the paper they have mentioned that the three longer devices in their study (2.2, 3.2, and 4 mm length) did not deliver any net gain.

Table 1 Gain characteristics of the 250, 700, and 1200 µm long hybrid slot waveguides

Waveguide length (µm)	Signal enhancement (dB)	Net modal gain (dB/cm)	Net material gain (dB/cm)
250	1.40 ± 0.15	20.1 ± 7.31	≥63.8 ± 23.2
700	3.69 ± 0.15	16.9 ± 4.70	≥53.7 ± 14.9
1200	6.28 ± 0.15	16.5 ± 4.36	≥52.4 ± 13.8

Table 1. Data taken from Rönn et al., 2019 [1].

The waveguide lengths measured were only 0.25, 0.7 and 1.2 mm and they have not reported the actual gain achieved but the per-cm-gain. In fact, the highest net gain achieved for each device was below 2 dB, when multiplying the extrapolated gain per cm with the device length (as given in Table 2 below), and in their follow-up papers they never achieved a gain coefficient of more than 3 dB/cm let alone 20 dB/cm [2].

Waveguide length (µm)	Net modal gain (extrapolated) dB/cm	**Actually measured gain (dB)**
250	20.1	0.5
700	16.9	1.2

Table 2. Data extracted from Rönn et al., 2019 [1].

1200	16.5	1.98

Moreover, we are not claiming a new method for gain film deposition. As the reviewer mentioned, RF sputtering is a well-established technique, as supported by several references already cited [references 34, 35, 37, 38, 40-42, 44, 46, 47, 49 in the updated main manuscript]. The novelty of our work lies not in the fabrication method itself, but in achieving several key performance milestones within the same device: high output power, a high small-signal net gain of 30 dB, an exceptionally low noise figure of 3.6 dB, and, most notably, the demonstration of polarization dependent gain (PDG) that can be tuned to absolute zero, an accomplishment that, to our knowledge, has not been achieved even in fiber systems, let alone in any integrated photonic platform.

References:

1. J. Rönn et al., "Ultra-high on-chip optical gain in erbium-based hybrid slot waveguides," Nat. Comm. 10, 432 (2019).
2. J. Rönn et al., "Erbium-doped hybrid waveguide amplifiers with net optical gain on a fully industrial 300 mm silicon nitride photonic platform," Opt. Express **28**, 27919 (2020)

The LMA waveguide architecture follows conventional scaling laws and design rules that have been established for in high-power integrated photonics. For example, Singh et al. recently published in Nature Photonics (2024) demonstrating "Watt-class silicon photonics-based optical high-power amplifier" with similar LMA approaches.

The performance improvements demonstrated here, while technically sound, represent evolutionary rather than revolutionary advances. The 30 dB total gain, when normalized by device length, does not dramatically exceed the gain per unit length achieved in previous integrated rare-earth amplifiers.

Yes, large-mode-area (LMA) and high-power operation have already been demonstrated in our earlier work [1]. In that study, more than one watt of output power was achieved in the power-amplification regime, where a seed signal of approximately 35 mW was amplified to beyond one watt. Such LMA devices are highly suitable for power amplification and have diverse applications, including both civilian and defense technologies. However, it has remained unclear whether an LMA device can also provide high amplification for weak input signals, that is, high small-signal gain. In the present work, we demonstrate a high net gain of up to 30 dB. It is important to emphasize that the total device net gain is the meaningful performance metric for any practical application, rather than the gain per centimeter. A common misconception in the community is that achieving, for instance, 3–5 dB/cm gain directly translates to 30–50 dB gain in a 10 cm device, or even 60–100 dB in a 20 cm device. In reality, optical amplifiers do not behave linearly with length in this manner. The achievable gain is fundamentally limited by the available pump power—the signal output can never exceed the pump power, even in the ideal case. In practice, the output signal power is further reduced by several factors, including signal reabsorption by ions, intrinsic propagation losses, gain saturation effects (which can be substantially mitigated through higher saturation power designs such as with large mode area), and amplified spontaneous emission (ASE), which becomes significant once the signal gain approaches 30–40 dB. A representative gain evolution along the waveguide length is illustrated in Fig. 1.

In the example shown in Fig. 1a), the steady-state rate equations were solved (see Supplementary Section 7) for a small-signal condition with a pump power of 1.5 W. As shown, with a short device length (for example, 5 cm), a gain of approximately 24 dB (equivalent to 4.8 dB/cm) can be obtained. However, as the length increases to 11 cm, the net gain saturates at around 30 dB, corresponding to 2.7 dB/cm. Although the

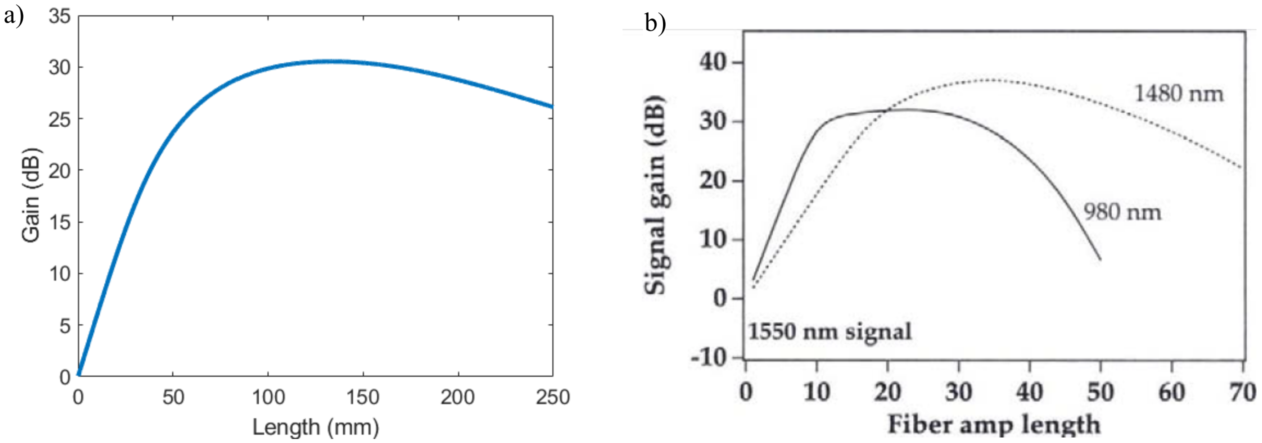

Fig. 1. a) Simulated gain as a function of length solved with steady state rate equations. b) Similar representative amplification curve in fiber amplifiers (length unit is meters) [2].

total gain increases, the gain per centimeter decreases. What ultimately matters from a device application view is the total extractable gain from the amplifier, not the per-unit-length value. While a shorter amplifier may exhibit a higher apparent gain-per-cm (e.g., 4.8 dB/cm), this does not imply that an 11 cm device would yield 48 dB of gain. Beyond approximately 11 cm in this case, the gain decreases due to signal propagation losses and diminished pump intensity along the waveguide, which limits ion excitation to the upper energy state. This effect is further exacerbated when the signal wavelength lies within the absorption band of the dopant ions—as is well known for erbium-doped systems—leading to reabsorption at longer propagation lengths. At higher pump powers, amplified spontaneous emission (ASE) in both forward and backward directions becomes significant, depleting the population inversion and thus capping the achievable signal amplification around 30–40 dB of net gain. Fig. 1b) shows a similar gain simulation of a typical erbium-doped fiber amplifier, which follows the same saturating gain behavior with length. The big difference in fiber amplifiers is the exceptionally low background propagation losses (< 0.01 dB/m, compared to ~ 10 dB/m in typical integrated waveguides), which allows for much longer devices (several meters long) with very low doping concentration that do not suffer as much from concentration quenching effects. Due to the low doping concentration, the gain coefficient can be as low as 0.03 dB/cm as in Fig. 1b) for a 10-cm-long fiber, but the total extracted gain from state-of-the-art fiber amplifiers often exceeds even 40 dB. Therefore, the most relevant performance parameter is the total net gain achievable from the amplifier, which depends critically on the optimized device dimensions, most importantly the combination of doping concentration and length. If the amplifier is too long, reabsorption and propagation losses dominate, while a device that is too short will fail to deplete the upper-state population efficiently, both leading to suboptimal gain.

The situation is even more severe for small-mode-area (SMA) devices, where strong gain saturation (due to lower saturation power, P_{sat}) suppresses amplification at high signal power levels. Consequently, high small-signal gain with SMA is achieved only at very low signal input powers, resulting in a relatively low saturation input power—the input signal power at which the gain drops by 3 dB from its maximum. To maintain the high gain even with high-power signals, the intrinsic saturation power of the amplifier needs to be increased, which we have shown with the large mode area waveguide design. Some experimental results for small-mode-area amplifiers are shown in Fig. 2. In this case, almost all waveguide parameters (device length, propagation losses, doping concentration, modal gain overlap factor, etc.) are very similar to our demonstrated LMA amplifier, only the mode area is roughly a factor 20 smaller compared to the LMA case. From the shown gain data with SMA, it can be seen that the gain starts dropping rapidly at signal input powers > 10 μW (-20 dBm). In comparison, with the LMA amplifier we achieve high gain up to > 200 μW signal input power, which corresponds to the > 100 mW saturation *output* power we have highlighted in the main manuscript (200 μW saturation *input* power + 27 dB gain at that power level = 100 mW saturation

output power). The saturation input power is roughly a factor 20 higher due to the 20 times larger mode area. Thus, one of the key results of this LMA amplifier demonstration is the ability to maintain the high

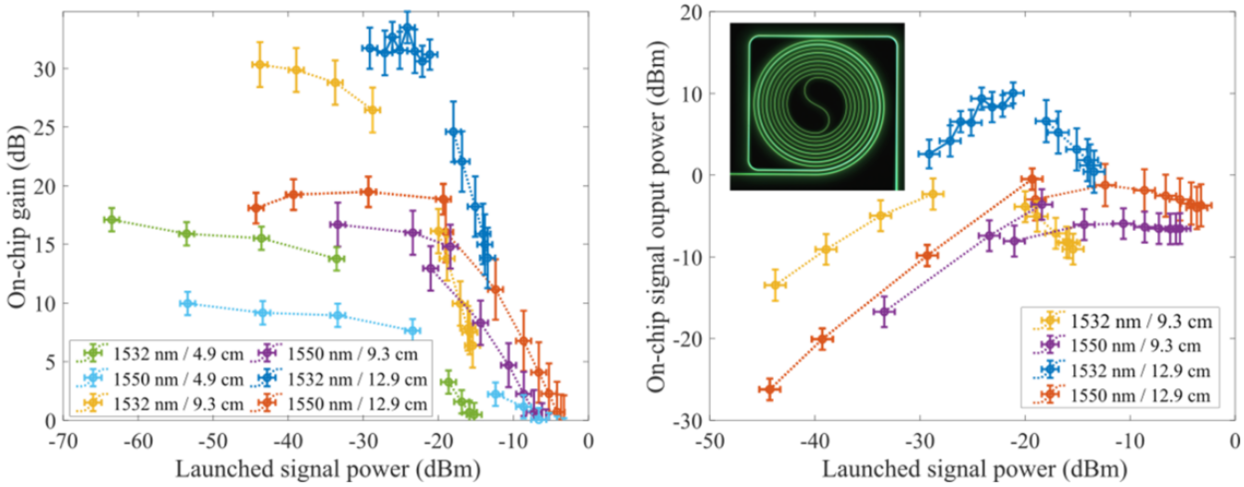

Fig. 2. Taken from reference [3]. Gain coefficients here at 1550 nm wavelength are ~ 2dB/cm (5 cm device), ~1.8 dB/cm (9.3 cm device), and ~ 1.5 dB/cm (12.9 cm device). At 1533 nm wavelength, where the emission strength of erbium is roughly twice as high, the gain coefficients are 3.4 dB/cm (5 cm device), 3.2 dB/cm (9.3 cm device), and 2.4 dB/cm (12.9 cm device).

gain for even very high signal powers, which is otherwise a major challenge in integrated amplifiers.

References:

1. N. Singh, J. Lorenzen, K. Wang, M. A. Gaafar, M. Sinobad, H. Francis, M. Edelmann, M. Geiselmann, T. Herr, S. M. Garcia-Blanco, and F. X. Kärtner, "Watt-class silicon photonics-based optical high-power amplifier," Nat. Photonics (2025).
2. P. M. Becker, A. A. Olsson, and J. R. Simpson, Erbium-Doped Fiber Amplifiers: Fundamentals and Technology (Elsevier, 1999).
3. D. B. Bonneville et. al "High on-chip gain spiral $Al_2O_3:Er^{3+}$ waveguide amplifiers" Opt. Express 32, 2024

The >115 mW saturation power, though notable, falls within the expected scaling behavior when moving to larger mode areas and represents a predictable engineering outcome rather than a breakthrough. The work by Singh et al. on watt-class silicon photonic amplifiers has already demonstrated power handling capabilities that likely exceed or match the current work's achievements. The authors have not adequately positioned their results relative to this very recent and highly relevant benchmark, raising questions about whether their performance claims represent meaningful advances over the current state-of-the-art.

We agree with the reviewer that the recent work has achieved high power, but that work didn't show *high power and high small signal gain from the same device.* For power amplification and pre-amplification usually two separate devices are used which are needed for example in fiber-based amplifiers. In the LMA amplifier, this is possible due to the drastically increased saturation power with the larger mode. This allows the gain to remain high even with high signal input powers, as we have discussed at the end our response to the previous questions. This is not the case in usual integrated amplifiers with a small mode area approach, because the gain saturates very quickly and even higher pump powers do not improve it much. Moreover, noise figures as low as shown here have not been achieved in any work so far. In integrated amplifiers noise figures are consistently well above 4 dB, as shown in Fig. 3. Our device approaches the fundamental

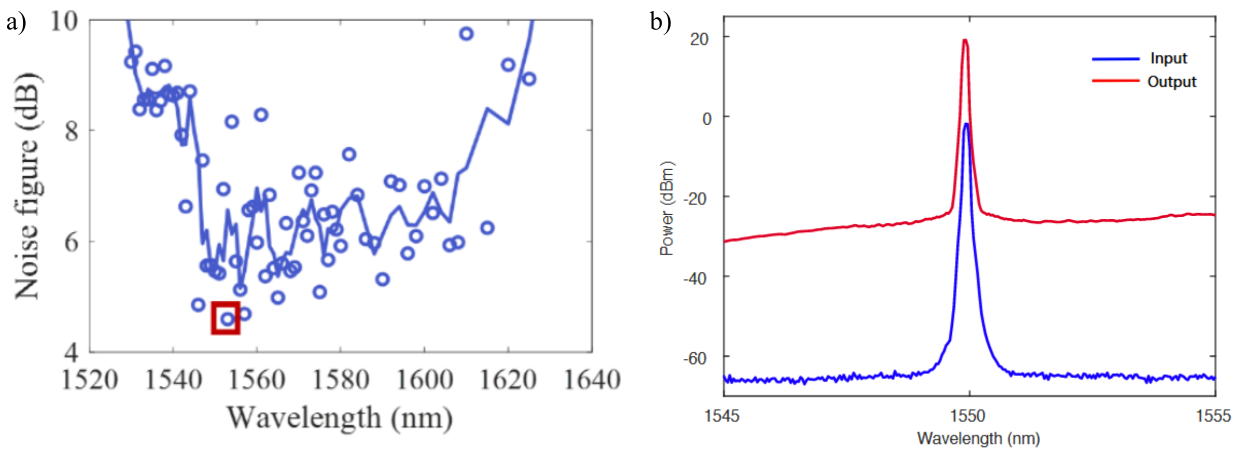

Fig.3. a) and b) Noise figures from recent works in integrated photonics with rare-earth doped waveguides. a) from [1]. b) Input and amplified output spectra from [supplementary of ref. 2], corresponding to a ~ 7 dB noise figure.

quantum limits of low-noise amplification, which is on the same level or even better than the best thulium-doped fiber amplifiers [3,4].

References:

1. C. E. O. Martinez et al., "Broadband Packaged Erbium-Doped Polycrystalline Al_2O_3 Waveguide Amplifier with 24 dB External Net Gain" Opt. Express 33, 2025
2. Y. Liu et al., "A photonic integrated circuit–based erbium-doped amplifier," Science, 376 2022.
3. Z. Li. et al., "Thulium-doped fiber amplifier for optical communications at 2 µm," Opt. Express, 21, 2013.
4. C. Romano et al., "Simulation of 2µm single clad thulium-doped silica fiber amplifiers by characterization of the $^3F_4$ –$^3H_6$ transition," Opt. Express **26**, 26080 (2018).

While the tunable PDG control is the most novel aspect of this work, it represents a relatively narrow technical capability that lacks the broad impact and fundamental significance expected for Nature Communications. The mechanism for PDG tuning, while useful for specific applications, does not reveal new physics or provide fundamental insights into light-matter interactions in integrated platforms. The capability appears to be primarily an engineering solution to a specific problem rather than opening new research directions or enabling transformative applications. Furthermore, the practical utility of precise PDG control in real-world systems is not convincingly demonstrated through system-level experiments or applications.

Yes, we have achieved a level of polarization-dependent gain (PDG) that has not been demonstrated even in fiber amplifiers, let alone in integrated photonic or semiconductor optical amplifiers (SOA). We respectfully disagree with the reviewers' view that this result represents merely an engineering solution. As is well known, PDG poses a significant challenge for inline amplifiers in telecommunications systems, as it directly limits the achievable bandwidth and average channel capacity. If this were an easily addressable issue, the industry would not have invested heavily in the development of symmetric fiber designs specifically to minimize PDG. One of the major reasons, aside from high noise figure and nonlinear effects, that semiconductor optical amplifiers did not succeed in long-haul communication systems is their inherently higher PDG compared to fiber amplifiers. Numerous patents and extensive research efforts have

aimed to mitigate PDG in SOAs; however, such approaches have proven difficult to implement effectively in practical systems (see Table 3).

The method commonly used in the semiconductor amplifier community to reduce PDG is to make use of polarization-diverse architectures, or by amplifying the signal in two passes within the same device, in forward and backward directions, with an added polarization rotation, see Figs. 4 and 5. These methods drastically complicate the device design.

In our work, we present a novel method for achieving **tunable polarization-dependent gain (PDG)** in integrated photonics, with the ability to **reduce PDG to 0 dB**. This level of polarization insensitivity surpasses even that of state-of-the-art single-mode fibers, which still exhibit small residual PDG due to fabrication imperfections. We demonstrate **complete PDG suppression** across a wide range of input signal powers, from a few microwatts to several milliwatts, which is especially challenging in SOAs where a

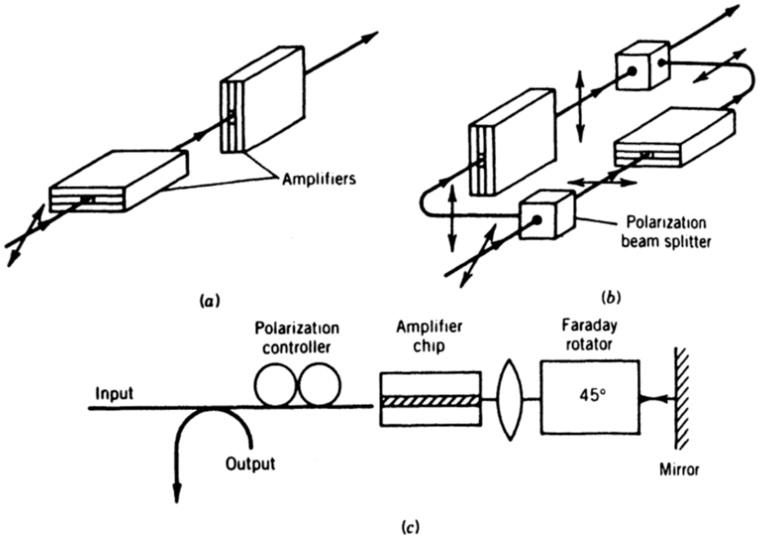

Fig. 4. Several schemes to reduce PDG in SOAs [1].

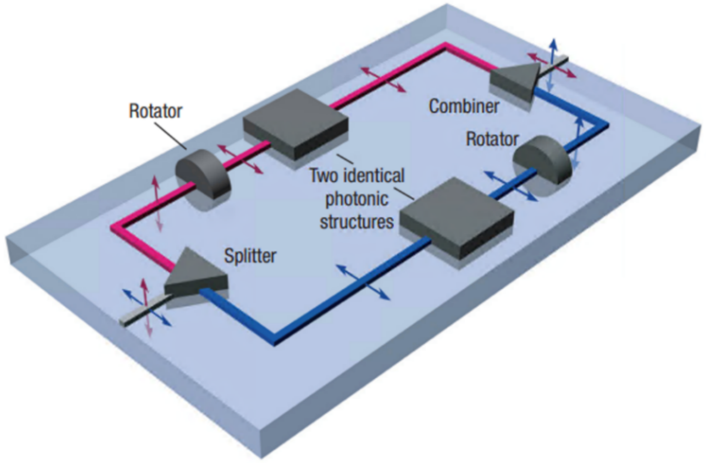

Fig. 5. In integrated photonics polarization diversity scheme is employed [2].

reasonably low PDG can usually only be achieved when the amplifier is driven into gain saturation, i.e. low-gain regime. So, we believe this is a major breakthrough in device development of <u>practical</u> importance and is not simply a matter of scientific curiosity.

References:

1. G. P. Agrawal, "Fiber optic communication systems", 3rd edition, chapter 6
2. T. Barwicz, et al., "Polarization-transparent microphotonic devices in the strong confinement limit" Nature Photonics 1, 2007

Reviewer #2 (Remarks to the Author):

The authors demonstrate a single integrated LMA amplifier capable of achieving both high-power amplification with output saturation powers >115 mW, high small-signal net gain up to 30 dB, and low-noise amplification with noise figures of ~3.6 dB. This concept is novel and the work could be considered for publication in Nature Communications after addressing the following concerns.

1. While high-power amplification over 1818-1948 nm is shown, and emission wavelength up to 2.1 µm is mentioned, a plot of the small-signal gain spectrum across the full potential bandwidth (e.g., 1700-2100 nm) would be very valuable to assess the true broadband potential.

Thank you for this recommendation and indeed we had not yet tested the full amplification bandwidth of our device across the entire possible emission spectrum. In a previous publication, we have demonstrated Watt-level amplification with a supercontinuum signal source that was spectrally filtered to our desired signal wavelength window (1800 – 2000 nm), and we have shown a gain spectrum there [1]. However, this was in the power amplifier operation with high signal input powers around 20-30 mW, so the gain behavior is not completely representative of the small-signal operation and the signal bandwidth was externally limited with the bandpass filter. For these reasons, we have now also measured the small-signal gain spectrum for the 6 and 15 cm-long amplifier devices. As shown in the main manuscript, the best gain performance was achieved with the 10.7 cm-long device, but unfortunately, during further high-power testing with different index-matching fluids the input facet of this waveguide got damaged, most likely due to deterioration of one of the tested fluids which was not able to withstand the prolonged high-power exposure. Due to a long down-time of the sputter machine for the gain film deposition, we did not have any more copies of this amplifier chip available for testing. Only since very recently the machine is functional again, so fabrication of chips with new gain films is now starting again. Therefore, we can only provide small-signal gain spectra for the other two amplifier devices with different lengths on that chip.

Small-signal gain measurements were performed with power-reliable index-matching glue (Norland NOA 148) to reduce facet reflection-based parasitic lasing. A supercontinuum source was used as the signal source with the signal spectrum spanning from 1300 to 2080 nm (there is a sharp drop-off beyond 2080 nm). The integrated signal input power launched into the amplifier waveguide was 0.4 mW and two on-chip pump power values were tested, 0.4 and 0.9 W. The measured gain spectra from the 6 and 15 cm-long amplifier

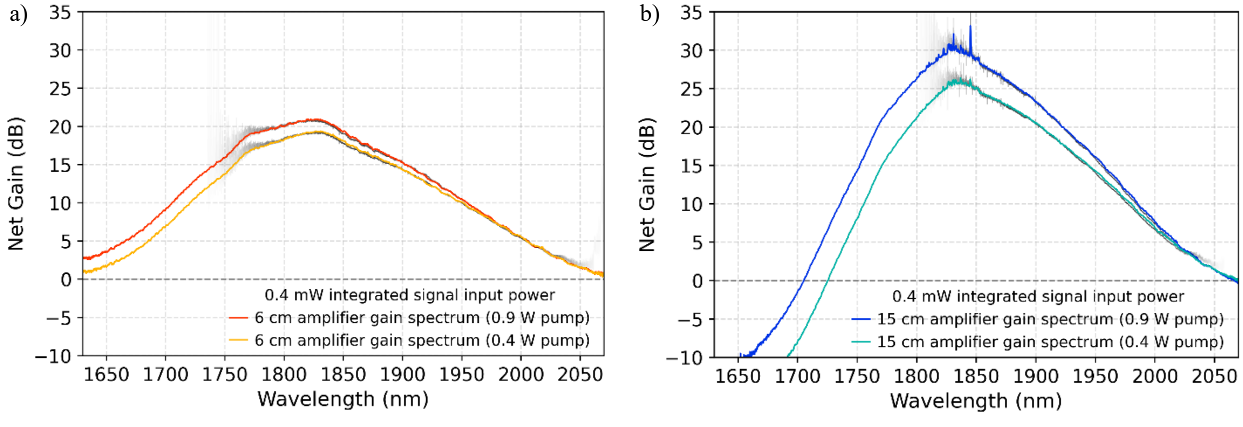

Fig. 6. Measured gain spectra in a) a 6 cm-long amplifier device and b) a 15 cm-long amplifier device. Both devices are on the same chip as the 10.7 cm-long device that was studied in the main manuscript, such that the waveguide and gain film dimensions are identical, only the device length is different. The integrated signal input power is 0.4 mW and the coupled pump powers are 0.4 and 0.9 W.

devices are shown in the following Fig. 6. The total integrated net gain at 0.9 W pump power in the 15 cm-long amplifier is ~ 25.5 dB and in the 6 cm-long amplifier it is ~ 17 dB, and the 3 dB gain bandwidth spans roughly 110 nm (1760 – 1870 nm) in the 6 cm device. In the 15-cm long amplifier, the pump power is not high enough to fully invert the entire amplifier length, so there is significant reabsorption at wavelengths below 1800 nm which reshapes the gain spectrum and reduces the effective gain bandwidth to about 70 nm. Characteristic features in the shape of the gain spectrum (e.g. the bump at 1760 nm) can be linked to the absorption and emission spectra of the thulium-doped gain medium, which are shown in Fig. 7b). A high level of inversion can be achieved in the 6 cm-long amplifier, so there is a little bit of gain even at the pump wavelength window (< 1650 nm). The optimal combination of length and concentration for both high-power and high-gain operation was in the 10.7 cm-long amplifier device, which was studied in the main manuscript, but the full small-signal gain spectrum could not be measured due to the device being damaged. However, before the damage on the device happened, we characterized the high-power amplification with the same supercontinuum signal source with an integrated signal input power of 25 mW coupled to the chip (integrated from 1600 to 2100 nm). This was done without index-matching glue, so the gain was limited to ~ 16 dB before parasitic lasing set in. Gain spectra were measured with 1.2 W on-chip pump power for the 6, 10.7 and 15 cm long amplifier devices and are shown in the following Fig. 7. The 10.7 cm-long device performed best in terms of overall gain. The longer 15 cm device again showed strong re-absorption at

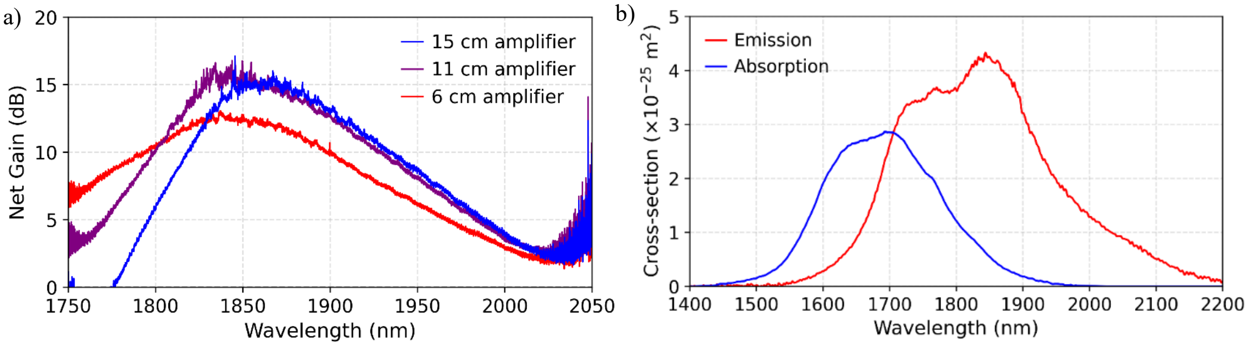

Fig. 7. a) Measured high-power gain spectra in 6, 10.7 and 15 cm-long amplifier devices. In this case the coupled signal input power was 25 mW and the on-chip pump power was 1.2 W. b) Measured absorption and emission cross-section showcasing the broad emission bandwidth of thulium spanning up to 2200 nm.

shorter wavelengths which impacted the overall gain and significantly more pump power would be required to reach a high level of inversion throughout the entire amplifier.

We measured the PL spectrum and the corresponding emission cross-section of the Tm-doped gain medium which shows emission up to 2200 nm as shown in Fig. 7. However, for the net gain of the amplifier also the background and component losses (e.g. taper transition loss, modal gain overlap) have to be factored in, which are optimized for the maximum gain around 1850 nm and losses increase towards shorter and longer wavelengths. For this reason, the effective net gain does not extend completely to the long edge of the emission spectrum and slight positive net gain is only achievable up to 2070 nm wavelength. Even though the emission bandwidth of thulium is quite broad (see Fig. 7), the emission strength and therefore the gain per unit length at long wavelengths > 1950 nm is significantly weaker than at the peak of the emission spectrum (~ 1850 nm). This makes it difficult to achieve uniformly high gain performance over the entire spectrum with just a single device, because the long signal wavelengths require more amplifier length to compensate for the lower emission cross-section. It is however possible to optimize the components of the amplifier waveguide design for operation at longer wavelengths and even introduce higher losses for short signal wavelengths to suppress the parasitic lasing at the peak of the emission spectrum. A longer amplifier waveguide would be required to achieve overall high gain and ideally the pump should be launched in

counter-propagating direction compared to the signal to improve the population inversion towards the end of the amplifier where the signal is strongest (see more on that later in response to question 3).

Reference:

1. N. Singh et al., "Watt-class silicon photonics-based optical high-power amplifier," Nat. Photonics 19, 307–314 (2025).

2. The manuscript mentions PDG robustness after tuning but doesn't explicitly show data demonstrating stability over time or against environmental fluctuations (e.g., temperature). Including such data would strengthen the claim of practical polarization insensitivity.

This is a valid concern considering that we are in a prototype testing phase of the amplifier device and we have now performed a 12-hour-long gain stability test. Recording the gain over longer periods of time is very challenging, because our amplifier chips are not fully packaged, so the fiber-to-chip coupling is not very stable. In our case, we require manual edge coupling with fibers on high-precision alignment stages (Thorlabs Nanomax) to be able to freely test also the various other test structures on every chip. This type of edge coupling tends to drift out of alignment within a few minutes when operating with high optical powers, which requires continuous manual realignment and makes long-term stability tests over several days practically impossible. However, for the high-gain measurements we have applied index-matching glue (Norland NOA 148, not UV-cured) to the chip's facets and fibers, which helped stabilize the coupling

a)

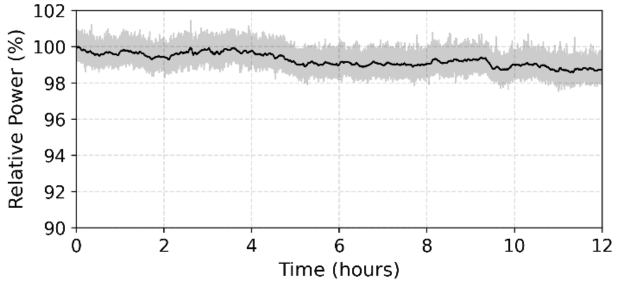

b)

c)

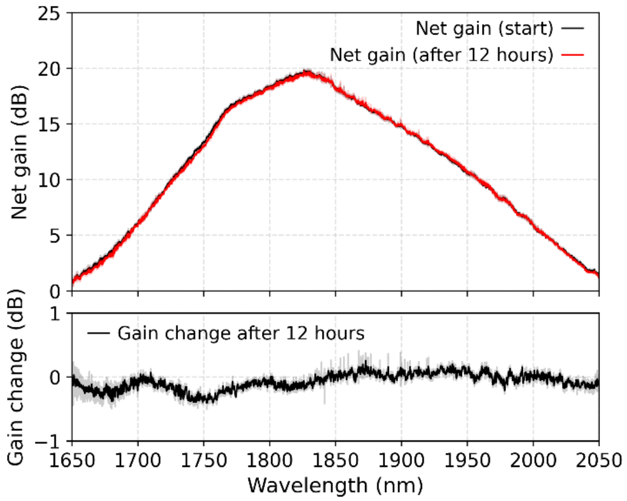

Fig. 8. a) Signal output power stability of a 6 cm-long amplifier device recorded over a 12-hour time period. The faint grey line is the measured raw signal showing power fluctuations of the seed laser, while the solid black line is a rolling average. The coupled signal power was 0.5 mW and the on-chip pump power was 0.7 W. b) Measured net gain spectrum before (black line) and after the 12-hour stability test (red line). The fiber-to-chip coupling was re-optimized after the 12-hour test to compensate the very slow drift over time. c) Change of the gain spectrum before and after the 12-hour test.

conditions by reducing thermal drifts and mechanical vibrations due to the high viscosity of the glue, such that no manual realignments were needed. With this, we performed a 12-hour power stability test with 0.7 W pump and 0.5 mW signal power launched into a 6 cm-long amplifier waveguide. The signal output power was recorded in 300 µs-intervals on a digital powermeter (Thorlabs PM100D), which is shown in the following Fig. 8. The power dropped very slowly by roughly 1 % over the 12-hour period due to still some slight misalignment over time, which could be manually readjusted afterwards. Small changes in the gain spectrum before and after the 12-hour measurement are not due to changes in the amplifier device, but are due to power fluctuations of the seed laser, which are transferred to the wavelength-axis in the spectrum due to the slow wavelength sweep of the spectrum analyzer. Because the power stability over time is an important aspect for any practical amplifier application, we have added the measured data also to the main manuscript as section 3.4.

Regarding temperature stability, it is known from the fiber-based amplifier technology that the operating temperature can have a noticeable impact on the gain performance. This has been studied for example in erbium-doped aluminosilicate fibers, which are commonly used in fiber-optic telecommunication [1]. There, a small reduction of gain at temperatures > 60°C can be linked to an increased absorption cross-section of the rare earth ions in the signal wavelength range. The higher temperature leads to a change of the occupation of the sublevels in each laser level manifold according to the Boltzmann distribution. This increases the occupation of higher sublevels in the ground state manifold, which leads to an increased absorption probability at longer wavelengths, i.e. more absorption loss at the signal wavelengths. This effect has also been observed in erbium-doped waveguide amplifiers based on a erbium-doped polycrystalline Al_2O_3 gain material [2], where the small-signal gain has dropped by 6.6 dB (from 24 to 17.4 dB) when changing the operating temperature from 30°C to 95°C. In another study of an erbium-doped amorphous Al_2O_3 amplifier [3], only a marginal net gain decrease of roughly 0.5 dB was observed when increasing the temperature from 20 to 140°C, indicating slight differences in temperature sensitivity between amorphous and polycrystalline hosts. In our device, while an overall small reduction of gain at high temperatures appears to be mostly unavoidable, we expect no significant effect on the PDG, because all characteristics of the TE and TM modes are nearly identical (eff. refractive index, spatial overlap with the gain layer, etc.) except for the mode area. Therefore, if an increased temperature leads to a gain reduction, it would reduce the gain for both polarization modes equally, thus leaving the PDG unchanged. To verify this experimentally in the future, we are currently acquiring the necessary equipment and built a setup to quantify the effects of high temperature on absorption and emission strengths.

References:

1. L. V. Kotov, A. D. Ignat'ev, M. M. Bubnov, and M. E. Likhachev, "Effect of temperature on the active properties of erbium-doped optical fibres," Quantum Electron. **46**, 271–276 (2016).
2. C. E. Osornio-Martinez, D. B. Bonneville, M. Dijkstra, A. R. Do Nascimento Jr., and S. M. García-Blanco, "Broadband Packaged Erbium-Doped Polycrystalline Al_2 O_3 Waveguide Amplifier with 24 dB External Net Gain," Opt. Express **33**, 28985 (2025)
3. S. A. Vázquez-Córdova, M. Dijkstra, E. H. Bernhardi, F. Ay, K. Wörhoff, J. L. Herek, S. M. García-Blanco, and M. Pollnau, "Erbium-doped spiral amplifiers with 20 dB of net gain on silicon," Opt. Express **22**, 25993 (2014)

3. The authors describe two approaches to achieve zero PDG: tuning the pump power or adjusting the pump polarization state. While on-chip polarization tuning presents challenges, they propose using a bidirectional pump scheme with two orthogonally polarized diodes as a solution. However, a key consideration arises: how would the significant Tm3+ ion absorption affects pump power uniformity along the 10 cm-long waveguide in this configuration?

Thank you for bringing up this question. This is a very important consideration especially in long amplifier devices such as the 10.7 cm-long amplifier used in this case, where a full population inversion cannot be maintained throughout the entire amplifier length as the pump is absorbed very strongly in the initial few cm (this device can already be considered to be “effectively long” due to the high thulium concentration, even though other low-concentration silicon photonics-based amplifiers are sometimes longer than 20 cm). To study the effects of different pump directions, we tested the gain with co- and counter-propagating pump setups with a 25-mW input signal, which are shown in the following Fig. 9. We see initially at low pump power that the counter-propagating pump setup provides approximately 0.5 dB more net gain than the co-propagating configuration. In the counter-propagating setup, the pump is inserted at the signal output side of the amplifier, meaning that the highest local population inversion is at the end of the amplifier where the signal is also the strongest. On the other hand, with the co-propagating setup the pump is inserted where the input signal is still weak and some pump light is lost due to propagation loss before it reaches the end of the amplifier, such that the inversion towards the signal output side is reduced. This is especially noticeable at low pump power, however, at high pump power the difference between the pump directions vanishes, as now the pump is strong enough to maintain a good level of inversion throughout the entire amplifier.

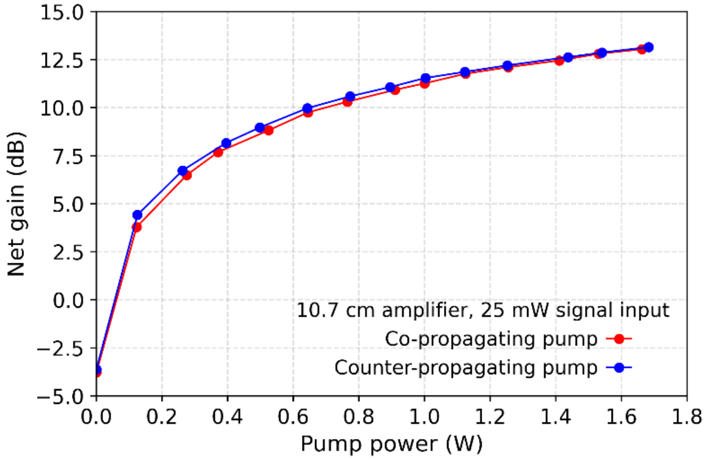

Fig. 9. Measured high-power net gain with the 10.7 cm-long amplifier as a function of pump power with a co-propagating pump (red) and a counter-propagating pump (blue). The coupled signal input power was 25 mW.

For the envisioned implementation of two pump diodes in a bidirectional pump configuration, there are some considerations: The PDG tests shown in the main manuscript were all performed with a co-propagating pump setup. These measurements indicated that the pump needs to be mostly TE polarized to obtain high gain for the TE signal mode, which implies that it would be more beneficial to place the TE polarized pump diode at the output side of the amplifier, launching the TE polarized pump in a counter-propagating way. Based on the previous pump direction test, this would also provide slightly more gain for the TE signal mode compared to the TM mode, which was necessary anyway to achieve 0 dB PDG. With this slight improvement of the TE signal gain, the overall required pump polarization state for 0 dB PDG would also shift away from a mostly TE polarized pump and gets instead closer to an equally mixed pump polarization state. This would also be a desired effect, because requiring a 100% TE polarized pump state in the amplifier with such an orthogonally polarized bidirectional pump setup would mean that only the TE polarized diode is actually on, while the TM polarized diode is off, which is not an effective way of pumping the amplifier. Placing the TE polarized pump diode towards the signal output side would improve the TE gain slightly, shifting the required overall pump state closer to an equal TE/TM mix, meaning that the total pump power can be more equally distributed to both diodes rather than just one diode having to provide most of the power. This would reduce the high power output requirements from each pump diode, as it is already a challenging tasks to implement high-power pump diodes in integrated photonics platforms to begin with.

4. The fabrication process of the device should be provided.

We have added a detailed description of the fabrication process in the first section of the supplementary material. We have put a special focus on the sputter deposition process of the thulium-doped alumina, because the quality of the deposited gain film is highly sensitive to the various parameters during the sputter process (e.g. applied power to the aluminum and thulium targets, argon and oxygen gas flow rates, deposition temperature, vacuum base pressure in the chamber, etc.). More information can be obtained from [1].

Reference:

1. K. Wang et al., "Thulium-Doped Al2O3 Waveguide Amplifiers Fabricated via Radio Frequency Reactive Co-Sputtering," 2025 25th Anniversary International Conference on Transparent Optical Networks (ICTON), Barcelona, Spain, 2025, pp. 1-4

5. Comparing the net gain performance and other key figures of merit of the proposed devices with previously reported works would strengthen the analysis.

Thank you for this suggestion. We have compiled an overview of reported gain characteristics comparing different amplifier technologies of the last 3 decades, including rare earth-doped fiber amplifiers, silicon photonics-based amplifiers, and semiconductor optical amplifiers (both integrated on photonic platforms and non-integrated devices). Here we only highlight a few notable results from each type of amplifier technology. For a broad overview on silicon photonics-based amplifiers and on-chip amplifiers (Si- and SiN-based waveguide amplifiers and heterogeneously integrated III-V semiconductor amplifiers) we would like to refer to the supplementary material of reference [1], which was focused on devices operating in the C-band (1.55 µm erbium window). For an overview of erbium-doped waveguide amplifiers, including thin film lithium niobate (TFLN), various oxides (Al_2O_3, TeO_2, Ta_2O_5), silicate and polymer waveguides, we would like to refer to reference [2]. In the following table we are comparing state-of-the-art amplifiers with a focus specifically on net gain, noise figure, PDG and PDG tunability, as well as output saturation power ($P_{sat,out}$). Although our LMA amplifier devices operate in the thulium wavelength window, we include here also many amplifiers operating in the erbium wavelength range (~ 1.55 µm), because the development of integrated amplifiers in the 2 µm wavelength range is severely limited. In the vast majority of chip-scale amplifiers, values for PDG are not reported because the devices are usually designed to operate in only one polarization mode and have an intrinsically very high PDG. Furthermore, output saturation powers are often not explicitly mentioned, but can sometimes be roughly estimated from the reported small-signal gain measurements. Lastly, we only list single amplifier architectures and exclude polarization diversity schemes (complex systems based on polarization beam splitters, polarization rotators and/or multiple amplifiers).

We believe this overview highlights how much of an improvement in output power the LMA amplifier design provides, which we have shown in our previous works and again in this work. Furthermore, our amplifier device is comparable or surpasses many commercial rare earth-doped fiber amplifiers in virtually every figure of merit. Meanwhile, semiconductor optical amplifiers (SOA) are able to provide very high output powers close to 1 W (e.g. slab-coupled optical waveguide amplifiers, SCOWA), however, achieving high output power, high gain and low noise figures simultaneously has been a big challenge. Furthermore, most SOAs have vastly different gain confinement factors for the TE and TM polarized signal modes leading to a very high polarization dependence. Only in special designs can the PDG be reduced to less than 0.5 dB, but mostly at the cost of output power.

We have also added this this comparison to the supplementary material as Section 6.

Table 3: Overview of key figures of merit of various optical amplifier technologies (TDFA/EDFA = thulium/erbium-doped fiber amplifier; EDFA = erbium-doped fiber amplifier; MQW = Multi-quantum-well; SCOWA = Slab-coupled optical waveguide amplifier; TDWA/EDWA = thulium/erbium-doped waveguide amplifier).

Ref.	Amplifier type	Max. net gain (dB)	Max. output power (mW)	Noise figure (dB)	Best PDG (dB)	Gain at best PDG (dB)	$P_{sat,out}$ (mW)	PDG tunability
**Rare earth-doped fiber amplifiers**								
[3]	TDFA	41	1200	~ 5	n.a.	n.a.	~ 250	n.a.
[4]	TDFA	40	140	3.1	n.a.	n.a.	~ 20	n.a.
[5]	Single EDFA	~ 40	~ 1	< 4.0	n.a.	n.a.	n.a.	n.a.
[5]	Dual-stage EDFA + isolator	54	~ 20	3.1	n.a.	n.a.	n.a.	n.a.
*a	Commercial EDFA	> 25	> 200	4.5	< 0.2	> 21	> 100	n.a.
**Various types of semiconductor optical amplifiers**								
[6]	Strained MQW	< 18	> 200	3.6	n.a.	n.a.	> 91	n.a.
[7]	SCOWA	< 15	~ 900	> 4.6	n.a.	n.a.	~ 800	n.a.
[8]	Quantum Dots	> 12	> 350	> 7	n.a.	n.a.	> 250	n.a.
[9]	Strained MQW	19	~ 90	7	~ 0.2	~ 18	50	No
[10]	Quantum Dots	~ 10	n.a.	n.a.	0.4	8	70	No
*b	Commercial packaged SOA	15	~ 30	8	1.0	15	~ 20	n.a.
*c	Commercial packaged BOA	28	~ 30	7	Very high	n.a.	~ 30	No (amplifies only single polarization)
**Photonic-integrated semiconductor optical amplifiers**								
[11]	III-V gain / Si (C-band)	27	56	~ 8	n.a.	n.a.	~ 30	n.a.
[12]	III-V gain / Si (C-band)	25	> 40	n.a.	n.a.	n.a.	42	n.a.
[13]	III-V gain / SiN (C-band)	14	~ 9	10.6	n.a.	n.a.	8	n.a.
[14]	III-V gain / Si (~ 2.0 µm)	13.1	18	17.2	n.a.	n.a.	n.a.	n.a.
**Silicon photonics-based rare earth-doped amplifiers**								
[15]	EDWA, etched Al_2O_3	20	~ 2.5	3.75	n.a.	n.a.	~ 0.4	n.a.
[16]	EDWA, etched Al_2O_3	33.5	21	n.a.	n.a.	n.a.	< 10	n.a.
[17]	EDWA, etched Al_2O_3 (packaged)	> 24.3	> 54	4.6	n.a.	n.a.	> 6	n.a.
[1]	EDWA, Er implanted SiN	30	145	~ 7	n.a.	n.a.	< 50	n.a.
[18]	LMA TDWA, power amp	16	1000	> 4.8	< 0.5	~ 10	n.a.	n.a.
[19]	LMA TDWA, power amp	13	1750	~ 5	n.a.	n.a.	n.a.	n.a.
**This work**	**LMA TDWA, high-gain operation**	**~ 30**	**> 800**	**3.6**	**0**	**> 21.5**	**> 115**	**Fully tunable over +1 to -1 dB**

Commercial amplifiers for reference:	
*a	Selection of commercial EDFAs:<br>• Thorlabs EDFA300S<br>• Thor F-PD-EDFA-25<br>• Nuphoton EDFA-CW-C4-HR-16-20-FCA<br>• bktel HPOA-1.5
*b	Thorlabs S7FC1013S (SOA – fiber coupled semiconductor optical amplifier)
*c	Thorlabs S9FC1004P (BOA – fiber coupled booster optical amplifier, only single polarization operation)

References

1. Y. Liu, Z. Qiu, X. Ji, A. Lukashchuk, J. He, J. Riemensberger, M. Hafermann, R. N. Wang, J. Liu, C. Ronning, and T. J. Kippenberg, "A photonic integrated circuit–based erbium-doped amplifier," Science **376**, 1309–1313 (2022).
2. X. He, Z. Zhang, D. Ma, C. Zhou, H. Hou, Y. Shuai, J. Liu, R. Wang, Z. Zhou, and W. Chen, "Erbium-doped/erbium-ytterbium co-doped waveguide amplifiers in silicon-based optoelectronics: recent progress," Adv. Photonics **7**, (2025).
3. Z. Li, A. M. Heidt, J. M. O. Daniel, Y. Jung, S. U. Alam, and D. J. Richardson, "Thulium-doped fiber amplifier for optical communications at 2 µm," Opt. Express **21**, 9289 (2013).
4. C. Romano, R. E. Tench, and J.-M. Delavaux, "Simulation of 2µm single clad thulium-doped silica fiber amplifiers by characterization of the^3 F_4 –^{3} H_6 transition," Opt. Express **26**, 26080 (2018).
5. R. I. Laming, M. N. Zervas, and D. N. Payne, "Erbium-doped fiber amplifier with 54 dB gain and 3.1 dB noise figures," IEEE Photonics Technol. Lett. **4**, 1345–1347 (1992).
6. K. Morito, S. Tanaka, S. Tomabechi, and A. Kuramata, "A broad-band MQW semiconductor optical amplifier with high saturation output power and low noise figure," IEEE Photonics Technol. Lett. **17**, 974–976 (2005).
7. P. W. Juodawlkis, J. J. Plant, W. Loh, L. J. Missaggia, F. J. O'Donnell, D. C. Oakley, A. Napoleone, J. Klamkin, J. T. Gopinath, D. J. Ripin, S. Gee, P. J. Delfyett, and J. P. Donnelly, "High-Power, Low-Noise 1.5-µm Slab-Coupled Optical Waveguide (SCOW) Emitters: Physics, Devices, and Applications," IEEE J. Sel. Top. Quantum Electron. **17**, 1698–1714 (2011).
8. T. Akiyama, M. Ekawa, M. Sugawara, K. Kawaguchi, Hisao Sudo, A. Kuramata, H. Ebe, and Y. Arakawa, "An ultrawide-band semiconductor optical amplifier having an extremely high penalty-free output power of 23 dBm achieved with quantum dots," IEEE Photonics Technol. Lett. **17**, 1614–1616 (2005).
9. K. Morito, M. Ekawa, T. Watanabe, and Y. Kotaki, "High-output-power polarization-insensitive semiconductor optical amplifier," J. Light. Technol. **21**, 176–181 (2003).
10. N. Yasuoka, H. Ebe, K. Kawaguchi, M. Ekawa, S. Sekiguchi, K. Morito, O. Wada, M. Sugawara, and Y. Arakawa, "Polarization-Insensitive Quantum Dot Semiconductor Optical Amplifiers Using Strain-Controlled Columnar Quantum Dots," J. Light. Technol. **30**, 68–75 (2012).
11. K. Van Gasse, R. Wang, and G. Roelkens, "27 dB gain III–V-on-silicon semiconductor optical amplifier with > 17 dBm output power," Opt. Express **27**, 293 (2019).
12. M. L. Davenport, S. Skendzic, N. Volet, J. C. Hulme, M. J. R. Heck, and J. E. Bowers, "Heterogeneous Silicon/III–V Semiconductor Optical Amplifiers," IEEE J. Sel. Top. Quantum Electron. **22**, 78–88 (2016).
13. C. Op De Beeck, B. Haq, L. Elsinger, A. Gocalinska, E. Pelucchi, B. Corbett, G. Roelkens, and B. Kuyken, "Heterogeneous III-V on silicon nitride amplifiers and lasers via microtransfer printing," Optica **7**, 386 (2020).
14. N. Volet, A. Spott, E. J. Stanton, M. L. Davenport, L. Chang, J. D. Peters, T. C. Briles, I. Vurgaftman, J. R. Meyer, and J. E. Bowers, "Semiconductor optical amplifiers at 2.0-µm wavelength on silicon," Laser Photonics Rev. **11**, 1600165 (2017).
15. S. A. Vázquez-Córdova, M. Dijkstra, E. H. Bernhardi, F. Ay, K. Wörhoff, J. L. Herek, S. M. García-Blanco, and M. Pollnau, "Erbium-doped spiral amplifiers with 20 dB of net gain on silicon," Opt. Express **22**, 25993 (2014).
16. D. B. Bonneville, C. E. Osornio-Martinez, M. Dijkstra, and S. M. García-Blanco, "High on-chip gain spiral Al_2 O_3 :Er^{3+} waveguide amplifiers," Opt. Express **32**, 15527 (2024).
17. C. E. Osornio-Martinez, D. B. Bonneville, M. Dijkstra, A. R. Do Nascimento Jr., and S. M. García-Blanco, "Broadband Packaged Erbium-Doped Polycrystalline Al_2 O_3 Waveguide Amplifier with 24 dB External Net Gain," Opt. Express **33**, 28985 (2025).
18. N. Singh, J. Lorenzen, K. Wang, M. A. Gaafar, M. Sinobad, H. Francis, M. Edelmann, M. Geiselmann, T. Herr, S. M. Garcia-Blanco, and F. X. Kärtner, "Watt-class silicon photonics-based optical high-power amplifier," Nat. Photonics **19**, 307–314 (2025).
19. N. Singh, J. Lorenzen, M. Kilinc, K. Wang, M. Sinobad, H. Francis, J. Carreira, M. Geiselmann, U. Demirbas, M. Pergament, S. M. Garcia-Blanco, and F. X. Kärtner, "Sub-2W tunable laser based on silicon photonics power amplifier," Light Sci. Appl. **14**, 18 (2025).

RESPONSE TO REVIEWER COMMENTS

We would like to thank the reviewers for taking their time to evaluate our work and give valuable comments which have further improved our paper. Please find our detailed response to the comments. In the revised manuscript the text is colored to highlight the changes.

I have carefully reviewed the revised manuscript, the authors' comprehensive response letter, and the additional data provided. While I appreciate the authors' detailed rebuttals and the effort to address previous concerns, I remain unconvinced that this manuscript presents the breakthrough advances required for publication in Nature Communications. The authors have provided extensive technical arguments defending their work, but these responses inadvertently highlight that this work represents incremental characterization and optimization of a recently published platform rather than transformative science. The following are the detailed reasons:

1. The authors dedicate substantial effort in their rebuttal to defending the novelty of their work, yet their arguments actually underscore the incremental nature of this contribution. The comparison with Rönn et al. (Nature Communications 2019) is particularly revealing. The authors point out that Rönn achieved only 1.98 dB actual gain in a 1.2 mm device despite reporting a 20 dB/cm gain coefficient, and they emphasize that "total device net gain is the meaningful performance metric for any practical application, rather than the gain per centimeter." This argument is entirely reasonable. However, the authors then acknowledge in their response that "large-mode-area (LMA) and high-power operation have already been demonstrated in our earlier work," referring to their Nature Photonics 2025 paper, where "more than one watt of output power was achieved in the power amplification regime." They state that what remained unclear was "whether an LMA device can also provide high amplification for weak input signals." This reveals the fundamental problem: the authors are essentially asking whether measuring small-signal gain in addition to large-signal gain in the same device architecture constitutes sufficient novelty for Nature Communications. In fact, demonstrating that a power amplifier can also function as a pre-amplifier when operated at different signal levels is an expected capability, not a breakthrough discovery. The detailed simulations provided in their rebuttal (Figure 1 showing gain saturation with length, comparisons with erbium-doped fiber amplifiers) explain amplifier physics, but these are well-established principles from the fiber amplifier literature that the authors are applying to their integrated platform. The authors themselves cite textbooks (Becker et al., 1999) showing that these gain saturation behaviors have been understood for decades.

Usually, power amplifiers and pre-amplifiers are implemented as two separate modules. A pre-amplifier is designed to boost very weak input signals, typically in the −40 to −30 dBm range, to output levels of a few milliwatts (and in some cases below 100 mW). In contrast, a power amplifier is designed to amplify relatively strong seed signals, ranging from hundreds of microwatts to milliwatt-level inputs, to output powers at the watt level. Consequently, a single system is generally not used to serve both functions. An example of commercially available amplifier modules illustrating this separation is shown in Fig. 1 (see, for example, products from DK Photonics: https://www.dkphotonics.com/product/). In our work, we demonstrate that the same device architecture can operate effectively in both regimes, serving as either a pre-amplifier or a power amplifier depending on the operating conditions. This is noteworthy because different design rules typically apply to fiber pre-amplifiers and power amplifiers, particularly regarding fiber length, core size, and numerical aperture (see Chapter 8 of Becker et al., *Erbium-Doped Fiber Amplifiers*). These parameters are usually optimized differently depending on whether the goal is high small-signal gain or high output power with saturation management.

Regarding Fig. 1 in our previous rebuttal: the purpose of that figure was to emphasize the importance of total net gain, rather than gain per unit length, which is often used as a comparison metric in integrated rare-earth amplifiers. The reviewer previously commented that “The 30 dB total gain, when normalized by device

length, does not dramatically exceed the gain per unit length achieved in previous integrated rare-earth amplifiers.” In response, we aimed to clarify that the most relevant performance parameter for an amplifier is the total net gain achievable from the device, not the gain per centimeter. The total net gain critically depends on the optimized device dimensions and system-level design, rather than on a normalized metric alone. We apologize if this point was already understood by the reviewer.

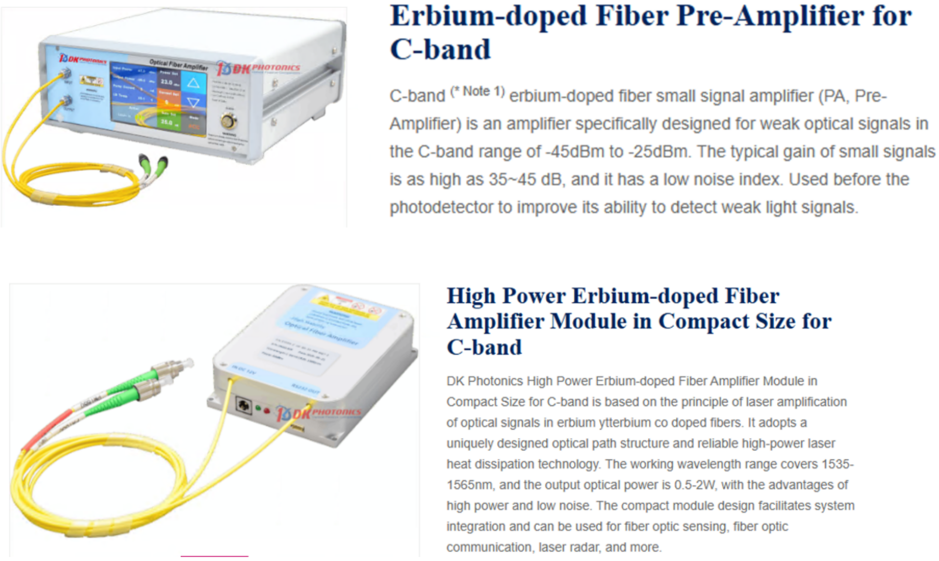

Fig. 1. Commercially available separate pre and high-power amplifier (DK Photonics).

2. The achievement of 30 dB gain is presented as a major contribution, but examination of how this was accomplished raises serious concerns about both scientific novelty and practical viability. In their response, the authors explicitly state that achieving gains above 16 dB required applying index-matching fluid or glue (Norland NOA 148) to suppress parasitic lasing from facet reflections. While this demonstrates good experimental technique, it fundamentally undermines claims of an integrated photonic breakthrough. More concerning is what the authors reveal about the experimental difficulties this approach created. They state multiple times that "during further high-power testing with different indexmatching fluids the input facet of this waveguide got damaged, most likely due to deterioration of one of the tested fluids which was not able to withstand the prolonged high-power exposure." This occurred specifically with their best performing 10.7 cm device. Later they note they "could not confirm the small signal 0-dB-point directly by measurement, because we avoided pump powers > 0.9 W since the index-matching fluid on the waveguide facets began to degrade from the prolonged high-power pumping." This fragility is not a minor implementation detail, but it prevented them from completing their characterization and destroyed their best device. They cannot claim to advance integrated photonics while relying on manually applied fluids that degrade under operating conditions and prevent full device characterization.

We fully agree with the reviewer that index-matching glue is not a reliable long-term solution and that anti-reflection (AR) coatings are required for a robust and practical device implementation. However, the primary objective of this experiment was to demonstrate the feasibility of achieving high net gain by reducing facet reflections. We employed index-matching glue because it is readily available and straightforward to apply, allowing us to experimentally validate the concept without the need for specialized fabrication processes. In contrast, implementing AR coatings requires carefully designed multilayer stacks of precise thicknesses deposited using dedicated sputtering or evaporation tools, which was beyond the scope of the present feasibility study. Importantly, our results confirm that suppressing facet reflections

significantly improves the achievable net gain. We believe that with an optimized inverse taper design, proper mode-size matching at the interface, and high-quality AR coatings engineered for both TE and TM polarizations, even higher net gains should be achievable. Based on our analysis, net gains in the range of 40–45 dB appear feasible and will be the focus of future work. We acknowledge and agree that index-matching glue is not a reliable long-term solution, as already stated in the manuscript, and we appreciate the reviewer's comment in highlighting this important practical consideration.

3. The authors report a noise figure of 3.6 dB at 30 dB gain and present extensive arguments in their rebuttal defending this as a key contribution. However, their own analysis reveals this is fundamentally expected performance rather than breakthrough science. They calculate the theoretical quantum-limited noise figure as 3.18 dB for their in-band pumped configuration, meaning they achieved performance only 0.42 dB above the fundamental limit. They correctly explain the physical mechanism: "the low spectral overlap of the pump and emission bands" in thulium results in "large differences between σem and σabs at the pump wavelength, which enables a high population inversion" leading to low spontaneous emission factor and thus low noise. They cite Romano et al. (Opt. Express 2018) demonstrating 3.1 dB noise figure in thulium-doped fiber amplifiers using the same pumping approach. The authors provide a comparison table (Table 3 in their rebuttal) showing noise figures across various amplifier technologies. This table actually undermines their novelty claim. Commercial EDFAs achieve 4.5 dB noise figures, and the 3.1 dB demonstrated by Romano in fiber TDFAs already approached the quantum limit seven years ago. They have not discovered new physics, broken fundamental limits, or enabled qualitatively new capabilities. Furthermore, the authors' own data show the noise figure increases substantially (approaching 5 dB) at higher signal powers due to gain saturation effects, as expected from well-understood amplifier physics. The authors provide no evidence that integrated implementation provides advantages over fiber solutions for low-noise applications.

We agree that the achieved noise figure is not lower than the best results reported in fiber amplifiers. However, the key point is that achieving comparable noise figure performance within an integrated photonics platform should itself be considered a significant milestone. As the reviewer noted already our previous 1 W output power work (Nature Photonics 2025): there also, generating 1 W is not a capability beyond what can be achieved with fiber amplifiers, in fact, fiber systems can readily deliver several tens of watts. However, the relevance of that work, as well as the present study, lies not in surpassing benchtop performance, but in reaching performance levels within an integrated photonic platform that were previously achievable only with benchtop technology. Integrated devices are subject to fundamentally different constraints, including higher propagation losses, tighter mode confinement, stronger parasitic reflections, and thermal limitations. Therefore, demonstrating fiber-level specifications, whether in output power or noise figure, within an integrated platform represents a substantial advance. In this sense, reaching fiber-class performance strengthens rather than weakens the impact of our results.

4. The PDG tuning capability is presented as the most novel contribution, and the authors' rebuttal includes the most vigorous defense of this aspect. They state that achieving "a level of polarization-dependent gain (PDG) that has not been demonstrated even in fiber amplifiers, let alone in integrated photonic or semiconductor optical amplifiers" represents "a major breakthrough in device development of practical importance." They argue that if PDG were easily addressable, "the industry would not have invested heavily in the development of symmetric fiber designs specifically to minimize PDG." However, examination of their experimental demonstration reveals significant limitations that contradict claims of a practical breakthrough. First, the PDG tuning requires continuous active control that is not currently implementable in an integrated fashion. The
authors demonstrate two tuning approaches: adjusting pump power or adjusting pump polarization. For pump power tuning, their data (Figure 4b) shows every change in signal power necessitates corresponding pump power adjustment to maintain zero PDG—a requirement for continuous active feedback control that adds substantial system complexity and is antithetical to the passive, stable

operation preferred in telecommunications. For pump polarization tuning, the authors acknowledge the fundamental implementation challenge. They propose integrating two orthogonally polarized pump diodes in a bidirectional scheme, but this remains entirely conceptual. Second, they present complex polarization diversity architectures from SOA literature as evidence that PDG is difficult to address, implying their approach is simpler. However, their approach requires bidirectional pumping with orthogonal polarization control and active feedback— comparable complexity to the polarization diversity schemes they criticize.

We agree with the reviewer's observation. In our approach, the desired polarization-dependent gain (PDG) can be achieved either by adjusting the pump power for a given signal power, or alternatively by tuning the pump polarization while keeping the pump power fixed. In most practical systems, the input signal power to an amplifier is determined by the fixed loss of the transmission fiber preceding it. For a given link length, this typically results in a relatively well-defined signal power at the amplifier input [1,2]. Under such conditions, the appropriate pump power or pump polarization state can be predetermined and optimized accordingly. However, we acknowledge that in certain scenarios, such as unknown transmission links or dynamically varying environments, the input signal power may fluctuate. In this case, our amplifier already demonstrates robust performance: as shown in Fig. 4d, the PDG remains within ±0.1 dB for signal power variations of ± 5 dB. For larger signal power variations, a straightforward solution would be to implement an input power monitor to dynamically adjust either the pump power or pump polarization.

It is worth noting that automatic gain control (AGC) and automatic power control (APC) are already standard features in commercial EDFAs, where dynamic pump power adjustment is routinely used to stabilize signal gain and output power. Therefore, the control strategy required here is fully compatible with established amplifier operation schemes.

Finally, we would like to clarify that pump polarization control does not necessarily require bidirectional pumping. It can also be implemented from a single side using a silicon photonics polarization combiner, which offers a compact and practical on-chip solution [3], see Fig.2.

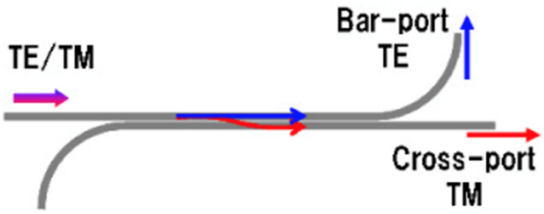

Fig. 2. TE and TM polarization splitter demonstrated in [3], which can be used as polarization combiner.

References

1. R. S. Tucker et al., "Green optical communications—Part I: Energy limitations in transport." IEEE Journal of selected topics in quantum electronics 17.2: 245-260, (2010).
2. M. Yamada et al., "Broadband and gain-flattened amplifier composed of a 1.55 μm-band and a 1.58 μm-band Er3+-doped fibre amplifier in a parallel configuration." Electronics letters 33.8: 710-711, (1997).
3. H. Fukuda et al., "Ultrasmall polarization splitter based on silicon wire waveguides," Opt. Express 14, 12401, (2006).

5. The timeline and overlap with the authors' recent publications remain deeply problematic despite their attempts to differentiate the work. The Nature Photonics paper (Ref. 50, published January 2025) demonstrated "Watt-class silicon photonics-based optical high-power amplifier" using the same LMA waveguide architecture. The Light Science & Applications paper (Ref. 51, also January 2025) demonstrated "Sub-2W tunable laser based on silicon photonics power amplifier" using the same platform. Both papers

are authored by the same research group, use the same silicon nitride/aluminum oxide platform, employ the same RF sputtering deposition techniques, and operate on the same physical principles. The authors argue in their rebuttal that the novelty lies in demonstrating high output power, a high small-signal net gain of 30 dB, an exceptionally low noise figure of 3.6 dB, and, most notably, the demonstration of polarization-dependent gain (PDG) that can be tuned to absolute zero all within the same device. However, Nature Communications explicitly discourages fragmenting research into multiple papers. Simply adding characterization metrics (noise figure, PDG) to a recently published platform is not enough.

In this work, we focus on a specific set of amplifier characteristics: high saturation power, high net gain (30 dB), ultra-low noise figure, and a novel tunable zero-PDG capability. Each of these aspects is nontrivial on its own, and combining all of them within a single study already represents a substantial scope. It would not be practical to simultaneously address every other possible functionality of the same device platform within one manuscript. It is common in integrated photonics for a single device platform to enable multiple distinct demonstrations over time. For example, a low-loss integrated waveguide may first be introduced in the context of linear photonics. Subsequently, the same structure may be explored for nonlinear phenomena such as four-wave mixing (FWM), Raman amplification, parametric gain, or supercontinuum generation. These represent fundamentally different operational regimes and research objectives, and they are typically reported in separate works. A similar situation applies to ring resonators. They may initially be studied for low-loss spectral filtering, and later investigated for nonlinear soliton generation, octave-spanning frequency comb formation, or low-noise microwave generation. Each of these represents a distinct and substantial research direction that cannot realistically be consolidated into a single publication. Historically, many integrated photonic platforms were first characterized for their linear properties, and only after years of exploration by multiple groups were their nonlinear or advanced functionalities uncovered. Such progressive and multifaceted investigation of the same physical device has been central to the evolution of integrated photonics as a field. Therefore, while the device presented here may enable additional functionalities beyond those emphasized in this manuscript, it would be neither practical nor appropriate to attempt to cover all possible aspects in a single paper. Our intention in this work is to highlight the specific novel amplifier features described above, which we believe constitute a clear and focused contribution.

Reviewer #2 (Remarks to the Author):

The authors have fully addressed my questions and now the manuscript is ready to publish.

Thank you.

Reviewer #3 (Remarks to the Author):

The authors have demonstrated integrated waveguide amplifiers based on a dual layer waveguide platform with a silicon nitride waveguide layer (with high confinement) and an Al2O3 large mode area waveguide layer. The amplification occurs in the Al2O3 layer.

The main impact of this work is in demonstrating amplifiers with high output power and high gain, and simultaneously, with the interesting property that, under some conditions, they can be “tuned” to a condition of zero polarization dependent gain (i.e. PDG = 0 dB). This is a substantial advancement compared to previous works on the same topic, especially because of the addition of the PDG = 0 dB feature which, while not perfect, can be further improved and hopefully optimized in future works. Based on these considerations, I think that this work deserve to be considered for publication on a high impact journal such as Nature Communications.

We would like to thank the reviewer for the positive feedback to our work. We have found the questions by the reviewer very constructive and they have helped us to further investigate our amplifier design and performance and improve our own understanding of the device.

Furthermore, with respect to the comments made by Reviewer # 1, I don't agree with his/her comments. Reviewer # 1 objections are based on his/her opinion that this work "represents primarily incremental improvements", that "suffers from insufficient novelty", and that essentially is not a breakthrough. In contrast, in my view, demonstrating an amplifier in integrated photonics with high power, high gain, low noise figure, PDG = 0 dB (granted, only in some conditions), all at the same time, is a significant advancement and deserves to be considered for publication on a high impact journal.

This said, I have the following comments/questions for the authors:

1. In regards to the measurements of polarization dependent gain (PDG) of Fig. 4 (Section 3.3): (a) Could the authors please confirm that when they state that the polarization of the signal is TE (for example, red markers in Fig. 4b) what they mean is that the light injected into the silicon nitride waveguide at the chip input facet is TE polarized? Conversely, if this is not the case, and if instead what they mean by "TE signal" is that the light is TE polarized only once it is coupled to the Al2O3 layer, then I wouldn't be sure that stating this amplifier has PDG = 0 dB is correct.

Thank you for pointing this out. Indeed, the signal is already TE polarized at the facet before coupling to the waveguide. We carefully ensure that the signal and pump light exiting the lensed fibers is in the desired polarization before placing the chip into the setup. We use a wire-grid polarizer in front of the lensed fiber and then adjust the signal and pump polarization with quarter- and half-wave plates at the laser sources. The polarization extinction ratio achieved with this method is typically > 23 dB, so that the signal and pump light injected into the waveguide is in a well-controlled polarization state. Specifically, during the PDG test with mixed pump polarization (i.e. Fig. 4c and d: 70% TE + 30% TM), the mix was achieved by tuning the half-wave plate at the pump laser source in-between its TE and TM orientation. This means that the pump light polarization at the waveguide facet is actually diagonal and the light couples partially into the TE and partially into the TM waveguide modes, because the waveguide only supports the fundamental TE and TM modes.

(b) Could the authors please confirm that TE-polarized light in the silicon nitride waveguide is coupled to the TE-polarized mode in the Al2O3 waveguide (and that TM-polarized light in the silicon nitride waveguide couples to the TM mode in the Al2O3 layer)?

We confirm that the TE or TM-polarized light maintains its polarization throughout the amplifier, including the vertical taper coupler (SiN-to-Al_2O_3 transition). We have tested this by injecting light, which was linearly polarized before the chip, into a waveguide and measuring its polarization at the waveguide output. Instead of using a lensed fiber at the waveguide output, we now put a lens to collimate the beam from the waveguide

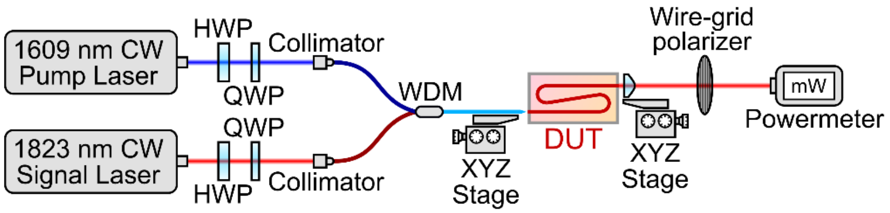

Fig. 1. Setup to measure polarization conversion through the amplifier waveguide. Signal and pump light are coupled to the waveguide with a lensed fiber, while the output is collimated with a lens and the polarization state is determined with a wire-grid polarizer.

and determine the output polarization state with a wire-grid polarizer. The setup is shown schematically in Fig. 1 below. To ensure that there are no polarization-dependent variations coming from the input fiber setup, we measured the input polarization exiting the lensed fiber in the same setup *without* the chip, and afterwards placed the chip into the setup without moving any of the input fibers.

First, we used signal light at 1823 nm wavelength in TE and TM polarization and confirmed that the polarization through the passive reference waveguide maintained the input polarization at the waveguide output. This waveguide only supports the fundamental TE and TM modes and no higher order modes. We could not detect any coupling from one polarization to the other within the measurement uncertainties of about – 24 dB (this was the minimum polarization extinction ratio we could reliably measure, meaning in the TE-polarized case, there was about – 24 dB residual TM polarized light component before and after the waveguide).

We then followed the same procedure with a 2-cm-long amplifier waveguide, which had only a single vertical coupler (SiN to Al_2O_3) on the input side and an extended LMA waveguide section all the way to the edge of the chip. The light then couples out directly from the Al_2O_3 layer to the lens, and we can observe the effects of a single vertical transition in isolation. Here we also did not see any conversion of one polarization to the other at the waveguide output. Only when coupling TE-polarized signal light, the main TE component was slightly weaker relative to the residual TM component by ~ 1 dB. This roughly matches the difference in propagation and absorption losses between TE and TM modes through the gain film, since the large TE mode interacts weakly with neighboring structures (due to limited space budget on the reticle), which leads to slightly higher losses compared to the smaller TM mode. Still, we conclude from this that the vertical coupler maintains the polarization without measurable coupling from one polarization to the other.

Lastly, we measured the output polarization state of a TE-polarized signal (~ 10 mW on-chip input power) through a 6-cm-long amplifier while pumping the amplifier with TE-polarized pump light (~ 300 mW pump power). In this case, we saw a relative improvement of the output polarization extinction ratio of ~ 2 dB favoring the TE component, which is expected due to the higher gain of the TE signal light component over the TM component when the pump is in TE, which is essentially just the on-chip PDG at this pump condition and signal power level. From these measurements we further concluded that pumping of the amplifier also does not affect the signal polarization state.

(c) Specifically, in reference to the pump power tuning of Fig. 4(b) to search for the pump value that leads to PDG = 0 dB, I think it is a downside of this approach that there is only one specific value of pump that provides PDG = 0 dB for every signal power level.
I understand that the authors may be suggesting that the solution to this problem is to tune the pump polarization as shown in Figs. 4(c-d), but as hinted by the authors as well it is rather impractical to implement this pump tuning approach in a realistic system.

We agree with the reviewer that the pump polarization tunability in practice remains somewhat challenging. However, we believe that the approach with two orthogonally polarized pump diodes can be realistically implemented, which would effectively allow for such a polarization tuning by changing the pump current of each diode. The pump light from the two diodes could be inserted in a bi-directional pump setup, which can be optimized to reduce the overall PDG even further. For example, based on our tests with co- and counter-propagating pump setups, we have concluded that inserting the TE-polarized pump in counter-direction would benefit the TE signal gain slightly more than the TM signal gain. This we have also discussed in response to the second reviewer's question 3. With the bi-directional pump setup, we expect a slightly more robust zero-PDG point against signal power fluctuations, because TE and TM signal gain would be slightly spatially separated due to the different pump insertion points. Otherwise, pumping from one direction with two orthogonal polarizations can also be achieved with polarization combiners based on directional couplers, in which the differently polarized pumps are combined from two individual waveguides into one amplifier waveguide. This has been demonstrated already in silicon waveguides (in this case it was a polarization splitter, but can be used in reverse to combine TE and TM polarization from two waveguides into one) [1], shown in Fig. 2, which can also be applied to the SiN waveguide platform.

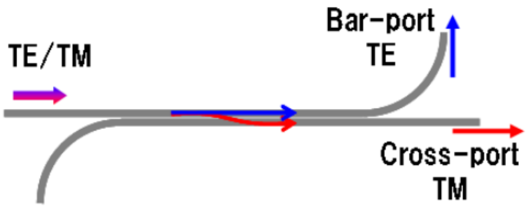

Fig. 2. Principle of a polarization splitter/combiner. Taken from [1].

Generally, we are very confident that the waveguide design can still be greatly improved to make the PDG performance overall more robust against pump and signal fluctuations. For example, in the demonstrated device, we tested the PDG performance over a 30 dB range of signal powers from 10 µW to 10 mW with fixed pump conditions. Within this range the PDG changed by 0.9 dB (manuscript Fig. 4d) without any pump tuning. If the signal input power in the system is roughly known within ± 3 dB, the PDG variation is already below 0.2 dB. This small amount of PDG tuning can already be achieved with a ~ 5 % change of pump power (estimated from manuscript Fig. 4b), would not affect the overall gain very much. For a field-deployed device, one could create a look-up table connecting any signal input power to the corresponding pump conditions for the optimal zero-PDG point. Then, for a given input power, one can adjust the pump power accordingly and the PDG will remain robust within < 0.1 dB for these pump and signal conditions, as long as the signal input power does not change too significantly. Such an automatic gain control (AGC) and automatic power control (APC) are already standard features in commercial EDFA systems. Hence, to minimize the PDG against any power fluctuations, one could include a small tap port to monitor the signal power and a feedback loop to adjust the drive current on the pump diodes to realign the PDG point.

Nevertheless, polarization insensitive amplification without requiring any tuning can be achieved with an improved waveguide design, that focuses on making the TE and TM modes equal in size, such that there is no difference in gain saturation behavior. This is somewhat challenging on the 800-nm-thick SiN platform used in this demonstration, because the TE mode in the LMA section is always larger than the TM mode (in this demonstration it is almost a factor 2 larger). In our demonstration, the large difference in mode sizes and saturation powers enabled the PDG tunability, but resulted in a potentially high PDG depending on the pump conditions (which could possibly be also a desired effect in some scenarios). One solution to keep the overall PDG low, is to shrink the Al_2O_3 layer in the horizontal direction to only ~ 50 µm width instead of having it extended over > 100 µm width, which would confine both TE and TM modes more strongly and with similar mode size and correspondingly similar gain behavior. On the other hand, realizing similar mode areas is very achievable on platforms with thinner SiN (e.g. 300 – 400 nm), which is also more common in most SiN foundries. With correct waveguide dimensions, the two polarization modes can be of identical size, which would lead to nearly identical gain behavior, but with a limited ability to tune the PDG. As an example, we show in Fig. 3 the mode profiles for the fundamental TE and TM modes, simulated with Lumerical's FDE solver. The effective mode area for both polarizations is identical at 20.6 μm^2. Here, the SiN waveguide is 350 nm thick and 660 nm wide. The SiO_2 layer between the SiN and Al_2O_3 is 300 nm thick (same as in the presented devices with 800-nm-thick SiN) and the Al_2O_3 gain film is 1100 nm thick (also similar to the presented devices).

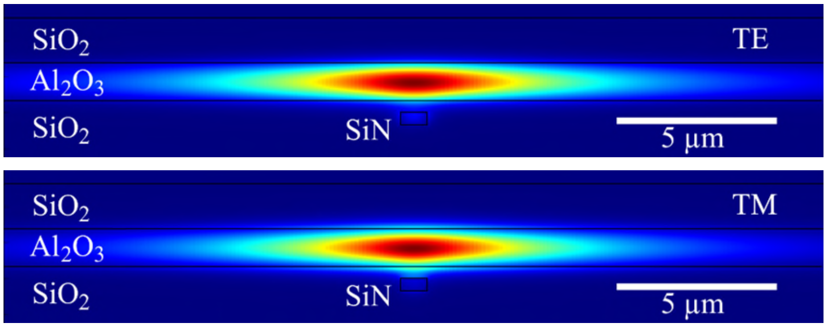

Fig. 3. Simulated polarized mode profiles for TE and TM polarization at 1550 nm wavelength in a 350 nm-thick SiN platform. The SiN width is 660 nm, and the Al_2O_3 film is 1100 nm thick. With these dimensions, the two modes are identical in effective mode area at 20.6 μm^2.

(d) One aspect that makes the authors' approach impractical is that it seems to me that the signals at the amplifier input must align with the waveguide axis (i.e. they must be TE and/or TM), otherwise for a differently polarized signal it would be even harder to find a PDG = 0 dB condition.

(e) I think that the best way to validate the authors' approach to achieve PDG = 0 dB would be to send two signals with orthogonal polarizations (TE and TM) at the waveguide input and check the gain experienced by the two signals. Has this test been performed?

Thank you for this suggestion. In response to both questions (d) and (e), we show here the test results with orthogonal polarizations at the waveguide input. The waveguide geometry only supports the fundamental TE and TM modes. Any differently polarized signal would couple partially into the TE and partially into the TM waveguide modes. In the test setup, we rotated the linearly polarized input signal to be diagonal at

a ~ 50° angle to the waveguide TE axis (slightly off from 45° to compensate for the 1.2 dB higher coupling loss of the TM component). That way, the signal light couples equally into the TE and TM polarized waveguide modes and both components have the same *on-chip* input power, which we have confirmed with a short passive reference waveguide. On the amplifier output side, we collimated the amplified signal with a lens to a free-space beam, to be able to determine the output polarization state. The setup is identical to the one shown in the Fig. 1 above. Having the signal output as a free-space beam allowed us to measure the power of each polarization component of the amplified signal individually by isolating it with a wire-grid polarizer. We also compare the results with completely TE and TM polarized signals. For every polarization orientation, we made sure that the same total signal input power of 0.5 mW is coupled to the waveguide. The pump polarization in this case was a 60/40 mix of TE and TM. The measured data is shown in Fig. 4a) and our expectation of the gain behavior is schematically shown in Fig. 4b). The achievable gain in this case was limited by a relatively early onset of parasitic lasing in the 6-cm-long amplifier at ~ 12 dB gain, because of a slightly different fabrication process where the chip facets were also coated with the Al_2O_3 gain film, leading to higher facet reflections due to the higher refractive index of Al_2O_3 (for the devices discussed in the manuscript, the chip facets were covered during the Al_2O_3 deposition to avoid such a facet coating). The 10.7-cm-long amplifier on this chip had slightly higher losses due to a small fabrication defect in the gain film. We would also like to note that the 6-cm device requires slightly lower pump power to achieve positive net gain compared to the 10.7-cm-long device shown in the main manuscript, because of the weaker re-absorption effects in the shorter length. Hence, the gain at 100 mW pump power in the short device is already ~ 10 dB, while it is only ~ 8 dB in the longer device. However, the longer device allows for more gain extraction when the pump power is further increased.

The results demonstrate that a signal with a mixed polarization state has the same zero-PDG point as fully TE or TM polarized signals. Moreover, at pump conditions that deviate from the zero-PDG point, the mixed signal gain (green dashed line) is exactly in-between the TE and TM gain curves (red and blue solid lines). We also noticed – when operating far away from the zero-PDG point – that the gain of the TE and TM components in the mixed polarization case (dotted red and blue lines) can have slightly stronger gain differences than when the signal is launched with a clean polarization. This happens, because the total input power in the mixed case is split into half TE and half TM, so that each component is of lower power and both are competing with each other for the gain. We have seen the same effect in the data shown in the main manuscript (Fig. 4), where the TM polarized signal was more strongly favored at low signal powers over the TE signal. Most importantly, however, the measurements confirm that every input polarization orientation converges to the same zero-PDG point, demonstrating that the zero-PDG condition does not require an alignment of the input polarization with the waveguide axis, and in fact every polarization orientation gets amplified equally.

We have now also added this data to the supplementary material section 5.4.

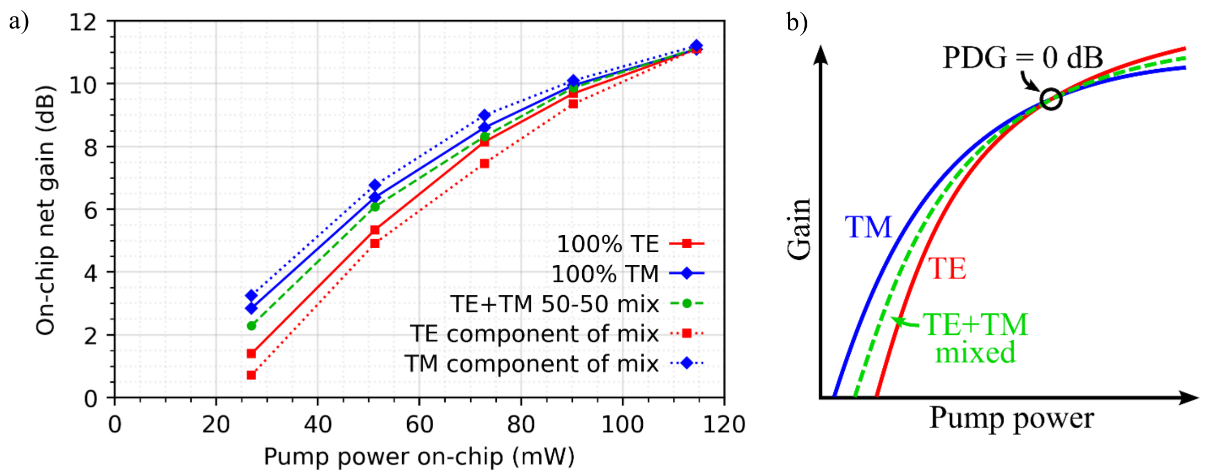

Fig. 4. a) Measured gain curves with a fully TE (red, solid line) and TM (blue, solid line) polarized input signal, a mix of 50% TE and 50% TM (diagonally polarized before coupling to the chip) with overall the same total input power (green, dashed line), and the measured gain of only the TE and TM components in the mixed case (red and blue, dotted lines). b) Concept drawing of the gain curves for TE and TM polarized signals and a signal with mixed polarization. Due to different waveguide and gain properties with the two polarizations (mode area, gain saturation power), the gain curves cross at a specific pump power, where the PDG = 0 dB.

The situation may appear different under fiber-to-fiber conditions, because of the difference in coupling loss between TE and TM polarized components of an arbitrarily polarized input signal at the facet. This would not be the case when the couplers are designed for equal coupling, which can be achieved easily with symmetric couplers (e.g. with a thin SiN waveguide platform), more on that in response to question 2. We have performed further measurements to verify that the same zero-PDG performance can also be achieved for off-chip (fiber-to-fiber) conditions. In this case, the amplifier has to be operated at a point where the *on-chip* PDG compensates the coupling loss difference between the TE and TM components of the input signal. Because the TM component couples slightly less to the waveguide than the TE component, it has to make up for the additional coupling loss with correspondingly higher *on-chip* gain. In this case, the coupling loss difference of 1.2 dB per facet requires an on-chip PDG of 2.4 dB to achieve the same overall fiber-to-fiber net gain for both polarization modes. To test the fiber-to-fiber zero-PDG performance, we recorded the amplified out-coupled power over time while manually adjusting the signal polarization with wave plates at the seed laser. We had no fiber-based polarization scrambler available for this test, so we chose to manually tune the polarization with the wave plates. Before the test, we determined the wave plate positions corresponding to TE, TM and mixed TE+TM polarizations at the waveguide facet and cycled through these positions in 1-minute intervals. The data is shown below in Fig. 5. The fiber-to-fiber PDG achieved in this test was ~ 0.05 – 0.10 dB, not exactly 0 dB, which can be mostly attributed to test setup instabilities, such as fluctuations of the fiber coupling stages over time, intensity fluctuations of the seed laser itself, and transmission irregularities of the wave plates at some orientations which caused small variations of the seed power (wave plate irregularities alone already contributed to ~ 1 % signal power variation between TE and TM orientation). Most of these instabilities would not be present in a properly packaged device. The pump conditions were tuned to the point where the measured fiber-to-fiber PDG was minimized. In this amplifier device, the required on-chip PDG of 2.4 dB could only be achieved at low pump powers and relatively low gain (on-chip gain ~ 12 dB, fiber-to-fiber gain only ~ 4 dB). This is identical to the device demonstration in the main manuscript (Fig. 4b), where the *on-chip* PDG is high *only* when the pump power is low (low gain) and is minimized in high-gain operation. This happens because the different gain saturation effects between TE and TM are more prominent when the gain is overall low and unsaturated and these differences get smaller when both TE and TM gain are saturated. Even though the measurement conditions for the fiber-to-

fiber PDG were not perfect, we believe this test verifies the principle of zero-PDG for both on-chip and fiber-to-fiber conditions.

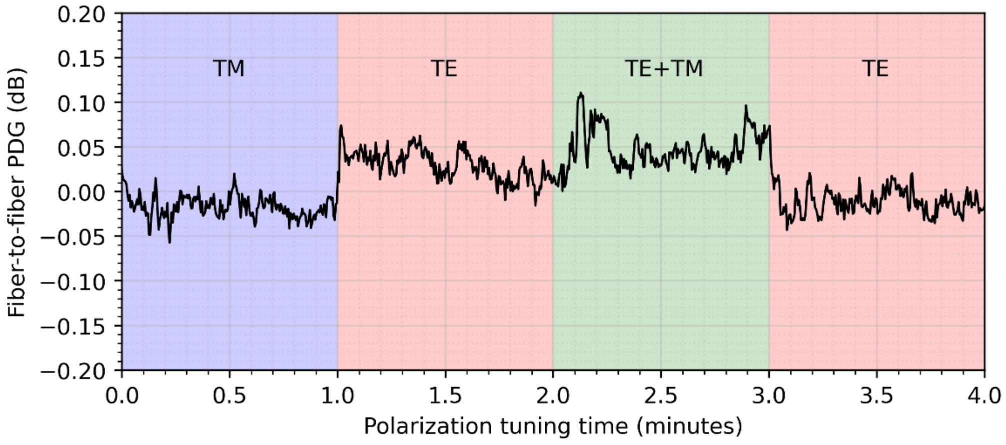

Fig. 5. Fiber-to-fiber PDG measurement over time. The signal input polarization is changed in 1-minute intervals by manually tuning wave plates at the seed laser.

In any case, the overall fiber-to-fiber performance (not only PDG, but also overall gain and noise figure) would benefit strongly from an improved fiber-to-chip coupler design, which emphasizes not only improved coupling loss, but also more similar losses for TE and TM polarization. In the tested devices, the coupler design was far from optimized for these test conditions. This was also one of the reasons why we decided to focus on *on-chip* performance values to disentangle the highly-performing amplifier waveguide from the relatively low-performing coupler design. A very thorough coupler optimization is still required for an actual packaged device solution, and we have already measured greatly improved coupling performance down to 0.7 dB per facet with negligible polarization dependence on new devices, which we discuss more in response to the next question.

2. The authors report power levels and gain of the signals referred to the “on-chip” condition, i.e. they de-embed the coupling loss from the fiber to the chip:
(a) This approach is rather confusing because unless the amplifier is integrated with something else, there is an input fiber and an output fiber. Hence, please report also the “off-chip” values for both signal power levels and gain, and also pump power levels.
(b) Also, what is the fiber to chip coupling loss for the signals and the pumps at their respective wavelengths?

We agree with the reviewer that it is more transparent to state the off-chip or fiber-to-fiber values for the amplifier performance metrics. This is especially relevant for the noise characteristics, since the *input* fiber-to-chip coupling loss leads directly to a degradation of the overall amplifier noise figure, as we discuss later in response to question 3. We had only listed the measured coupling loss values in the methods section at the end of the main manuscript, but have now also added important off-chip performance values in the main text next to their on-chip values. The coupling losses depended on the specific index-matching glue that was applied to the lensed fibers and the chip facets. With the glue applied for the high-gain and noise figure measurements, the coupling losses were 2.2 dB/facet at the signal wavelength (~ 1818 nm) and 2.0 dB/facet for the pump wavelength (1609 nm) in TE polarization. For the PDG measurements, another glue with different refractive index was chosen, which lead to different coupling losses: 3.9 and 4.9 dB for the TE and TM polarized signal light, and 4.7 dB and 7.1 dB for the TE and TM polarized pump light, respectively.

Our focus in this report so far was on the *on-chip* amplifier performance. We were interested in the isolated gain waveguide performance, that is specifically independent of coupling losses, because these losses can be relatively easily changed with improved coupler designs. By focusing on on-chip performance, we can reliably compare our LMA waveguide design with other recently published waveguide architectures. In our devices, we have used a PDK element from the SiN foundry (LIGENTEC SA) for the inverted taper couplers, which were designed for 1550 nm wavelength, not 1850 nm. At 1550 nm, the coupling loss is around 1.5 – 2.0 dB per facet with 2.5 µm spot size lensed fibers. At longer wavelengths ~ 1850 nm with the same lensed fiber, the coupling is quite different due to the different mode profile at the waveguide facet at longer wavelengths. Even more variation was brought in with different index-matching fluids applied to the waveguide facet to reduce reflections. This changes the lensing behavior of the coupling fibers, creating very different focus spot sizes depending on the exact index of the fluid. Therefore, the only way to obtain really comparable device performances under different coupling conditions was to de-embed coupling losses and use only the on-chip values. In this demonstration we were limited by the 800-nm-thick SiN platform and the available PDK elements, however, for our new and improved amplifier waveguide designs, we have put more effort into optimizing the fiber-to-chip coupling to also improve the off-chip performance. For example, we have already tested inverted taper couplers on a 350-nm-thick SiN platform with measured coupling losses of 0.7 – 0.8 dB per facet and only ~ 0.1 dB coupling difference between TE and TM polarization. This may be further improved down to 0.2 dB loss per facet, which has been already demonstrated on a SiN platform [2], and such modifications can also be implemented for wavelengths around 1.8 – 2.0 µm. To avoid the need of index-matching glue, we have also tested couplers at an angle to the chip facet, for which we have measured significantly lower facet reflections down to roughly – 50 dB instead of – 15 dB when the couplers is not angled. We have measured this using optical frequency domain reflectometry (OFDR). Such angled couplers help to suppress the parasitic lasing drastically but limit the flexibility in coupling and packaging, as the fiber has to be introduced with an angle to the facet. This approach is commonly used in lasers based on reflective semiconductor optical amplifier technology (RSOA) combined with an external cavity, where the waveguide of the RSOA element is angled to reduce internal reflections and parasitic lasing in the RSOA. A more practical solution is to deposit an anti-reflective coating (AR coating) onto the facet, which can be engineered to the targeted waveguide index and wavelength and would ease the fiber coupling conditions, as no angled couplers are needed.

We believe that the ~ 2 dB coupling loss in the high-gain and noise figure tests shown in the manuscript is already respectable. However, we are confident that more improvement can be found in a proper optimization process, especially when a specific target application is desired (i.e. how exactly the chip is packaged, type of fiber-to-chip bonding, etc.). We have already seen nearly polarization-insensitive coupling losses down to 0.7 dB per facet in a new amplifier design and there are reports of 0.2 dB coupling loss on a thinner SiN platform demonstrating such improvements [2].

3. In the evaluation of the noise figure, have the authors de-embedded the fiber to chip coupling loss from the NF values provided or not? In other words, are the numbers reported in the NF section (3.2) referred to "on-chip" conditions? Once again, if this is the case, it is confusing as you have an input fiber and output fiber, and if this is the case please report the NF values from fiber to fiber.

Thank you for highlighting this. We had indeed not specified that the reference points used for the noise figure measurements were all on-chip values, which we have now added to the manuscript to avoid this confusion. The noise figure was extracted with gain and power levels calibrated to the on-chip conditions. This means explicitly, that the signal input power was considered to be the coupled on-chip input power,

and for the signal output power, the output coupling loss and additional fiber component losses after the amplifier were added back to the power levels measured on the OSA to obtain the on-chip signal output level and on-chip gain. The coupling loss per facet was 2.2 dB and the total losses on the output side (output coupling loss, WDM loss, spectrum measured on a 10% drop port, connector losses, and insertion loss to the OSA) were measured to be 25.5 dB. To the best of our knowledge, this is one of the correct procedures to obtain an isolated noise figure value for only the on-chip amplifier. Alternatively, we also used the measured fiber-to-fiber gain and power levels to obtain the fiber-to-fiber noise figure and subsequently calculate the corresponding on-chip noise figure by de-embedding the coupling losses according to Friis' formula. It seems that both methods lead to the same results. We would like to explain our analysis process in some detail in the hopes that the reviewer can ensure that our approach and understanding is correct.

As we understand, it is important to specify the relevant reference planes for the input and output power level of the signal and ASE to properly evaluate either the on-chip or fiber-to-fiber NF value. In a practical implementation of the amplifier system, all external losses can ideally be reduced to only the fiber-to-chip coupling loss of 2.2 dB. Therefore, for the fiber-to-fiber noise figure, we consider only the input and output coupling fibers as the reference planes, while all the extra fiber component losses on the output side for performance monitoring were combined into a correction factor to the measured power levels. We understand that most important are any losses *before* the amplifier, as any lossy element would lead to a degradation of the *input* SNR equal to the loss of the element [3]. As the input coupling loss to the chip is 2.2 dB, this would already lead to an increase of the fiber-to-fiber NF by 2.2 dB. Any lossy element *after* the amplifier has to be treated with Friis' formula for cascaded amplifier/loss components. We also compare this with a similar example in [3]. As we noticed, the additional loss *after* the amplifier has only a minor effect on the total noise figure of the system, unless the additional loss becomes similar in size to the amplifier gain. We use Friis' formula for the total noise factor of a cascade of three amplifiers/loss components as given by

$$F_{\mathrm{tot}} = F_1 + \frac{F_2 - 1}{G_1} + \frac{F_3 - 1}{G_1 G_2} \quad (1).$$

In our case, $F_1 = 1/L$ and $F_3 = 1/L$ are the noise factors due to the coupling loss L (decreasing SNR due to shot-noise) at the input and output [3], respectively. F_2 is the *isolated on-chip* noise factor (calculated with NF equation 2 below, using on-chip power levels) of the amplifier (signal-spontaneous beat noise + the shot-noise component $1/G_2$ with the amplifier gain factor G_2), and $G_1 = L$ is the input coupling loss. All units are in linear scale meaning output power divided by input power. The measured coupling loss was $L = 0.60$ (-2.2 dB) and the lowest tested amplifier gain was $G_2 = 10$ (10 dB) with a corresponding on-chip noise factor of $F_2 = 5.5$ (7.4 dB). Using equation 1, the resulting fiber-to-fiber noise figure is $\mathrm{NF_{f\text{-}f}} = 9.65$ dB, which includes now the 2.2 dB coupling loss per facet and is 2.25 dB higher than the measured on-chip noise figure of 7.4 dB. Crucially, the *output* coupling loss only adds 0.05 dB to the total noise figure. The best measured on-chip noise figure of 3.6 dB at 29.8 dB gain corresponds to a fiber-to-fiber noise figure of 5.8 dB. In that case, the output coupling loss adds practically nothing to the noise figure, because $G_2 >> L$, such that the output coupling loss becomes negligible (third component in eq. 1). We see that even if the entire 25.5 dB loss of the fiber test setup on the output side were considered (meaning there is almost no fiber-to-OSA net gain) the noise figure would still only increase by another 0.6 dB, because still $G_2 > L$.

To verify these noise figure values, we also calculated directly the fiber-to-*OSA* noise figure with the output signal as measured on the OSA, which includes all 25.5 dB fiber component losses on the output side and also the input coupling loss. In that case, the total fiber-to-OSA net gain measured on the OSA was only G_{tot} = 2.15 dB, which matches to the 29.8 dB on-chip gain with -25.5 dB output loss and -2.2 dB input coupling loss. The measured ASE power at the signal wavelength was P_{ASE} = 3.28 nW in the equivalent noise bandwidth of the OSA B_0 = 0.053 nm = 4.81 GHz (at 1818 nm signal wavelength), and G_{lin} = 1.64 (2.15 dB). Using the noise figure formula

$$\mathrm{NF} = \frac{P_{\mathrm{ASE}}}{G_{\mathrm{lin}}\, h\nu\, B_0} + \frac{1}{G_{\mathrm{lin}}} \quad (2),$$

we calculate directly a fiber-to-OSA noise figure of 6.4 dB. Inverting Friis' formula and using $G_{\mathrm{tot}} = G_2 -$ 25.5 dB – 2.2 dB to obtain the isolated on-chip gain value, we obtain an on-chip noise figure of 3.6 dB, which is identical to the value reported in the main manuscript.

As an exercise for ourselves and to deepen our understanding, we also performed a simple test to confirm that additional losses *after* the amplifier do not significantly increase the noise figure, as long as the amplifier gain is higher than the losses. We believe this confirms that we calculate on-chip and off-chip NF values correctly. For this, we measured the noise figure of a 6-cm-long amplifier using a similar setup to the one described in the main manuscript, only that we now inserted additional losses directly before the OSA in the form of fiber attenuators with fixed losses. We tested the amplifier in low-gain operation with 11.4 dB on-chip net gain. The coupling losses in this case were 2.6 dB per facet and the total losses after the amplifier (including output coupling loss, fiber components and the additional fiber attenuators) ranged from 10 to 29 dB. We measured the noise figure off-chip (fiber-to-OSA) by taking the ASE power and fiber-to-fiber gain directly from the OSA data without any power level adjustments (Red dots in Fig. 6). From this measured *off*-chip NF, we calculated the corresponding *on*-chip NF by inverting Friis' equation as above (+ symbols in Fig. 6). Then, for comparison, we reversed the procedure and extracted first the *on*-chip noise figure by adjusting the gain and the measured power levels on the OSA with the coupling and fiber losses (Green diamonds in Fig. 6). Subsequently we used the so-measured *on*-chip NF in Friis' formula again to calculate the corresponding *off*-chip NF including all input and output losses (x symbols in Fig. 6). As we expected from Friis' equation, both methods yield the same results, as it is only a matter of changing the power levels to the relevant reference points in the system. We can also identify, that the additional losses *after* the amplifier only become significant when they become similar or larger than the amplifier gain, which is around 10 dB in this case. In the case of 0 additional output losses, the on-chip and fiber-to-fiber noise figure differ by exactly the *input* coupling loss, which can be improved down to 0.2 dB as discussed above, making the difference between the on-chip and off-chip noise figure negligible.

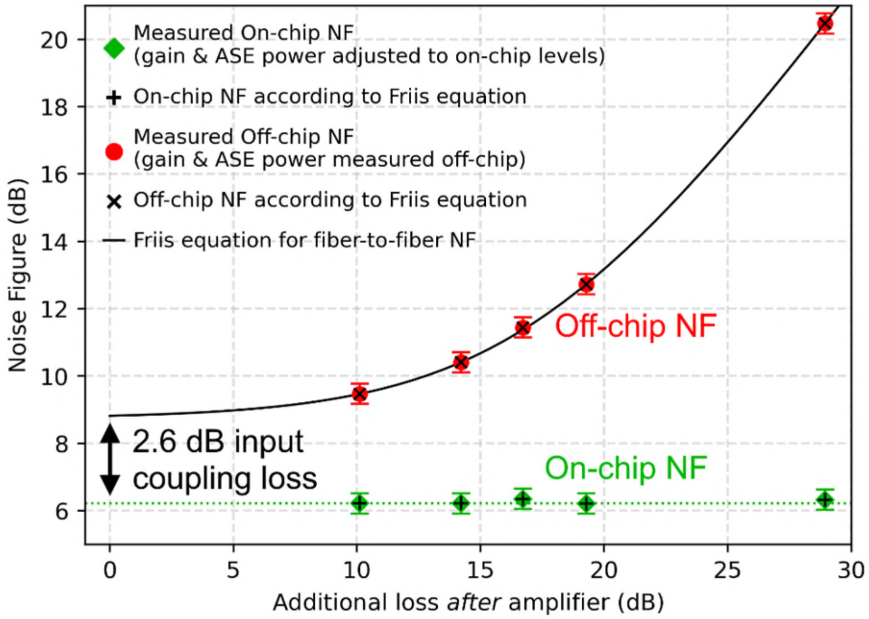

Fig. 6. Measured noise figure of a 6-cm-long amplifier according to different reference planes: On-chip and off-chip (fiber-to-OSA) power levels and comparison with corresponding values calculated with Friis' formula. The on-chip net gain of the amplifier was 11.4 dB.

4. The authors call this amplifier a “silicon photonic amplifier”, however there are no silicon photonic components or silicon waveguides in this work. The amplifier is made by a dual layer waveguide technology incorporating a silicon nitride waveguide layer and an Al2O3 layer where the amplification occurs. There is no silicon photonic waveguide here, so please do not call this a silicon photonic amplifier. This is an amplifier for silicon nitride waveguides. In my opinion, it can be called a silicon photonics amplifier only if integration with a silicon waveguide is shown.

We apologies if this was confusing and not clear. We use the term silicon photonics because we obtain the wafer (with chips) from a silicon photonics foundry (LIGENTC SA) which uses a deep UV process on a silicon photonics wafer to pattern the photonic devices used here (e.g. imec, https://www.imec-int.com/en/what-we-offer/development/photonics, or CEA-Leti, https://www.leti-cea.com/cea-tech/leti/english/Pages/Applied-Research/Technology-Fields/Silicon-Photonics.aspx).. Such a wafer includes essential layers such as silicon, silica and silicon nitride which are standard layer stack provided by any silicon photonics foundry. We previously had a description of the fabrication process only in the supplementary material, but had missed to mention the foundry and fabrication steps in the main manuscript. We have now added this information in the methods section.
If the reviewer was referring to “a CMOS-compatible amplifier”, then we agree that the fabrication process does not use CMOS wafers, in which highly doped silicon handle wafers are used (for active components) and therefore high-temperature processes such as LPCVD silicon nitride cannot be used (but progress is also being made on that front [4]). Hence, many foundries offer LPCVD silicon nitride, which is used here and is considered part of a silicon photonics process rather than a CMOS-process. But if the reviewer feels strongly against the term “silicon photonics” then we are happy to remove it from the title.

5. The authors state in a few points in the text that SOAs are polarization sensitive. However, today there are several vendors that have commercialized and sell polarization independent SOAs (i.e. where the SOA waveguide is actually polarization insensitive, without using a polarization diversity scheme). Please correct this.

It is true, there are many commercially available SOAs marketed as polarization-insensitive. However, we have found that (according to available specifications from the vendors) these SOAs still have a residual PDG, often around 1 dB, and are not on the same low-PDG level of fiber amplifiers, which are around 0.1 dB or even lower. To our understanding, semiconductor-based amplifiers are mostly split into SOAs and BOAs (booster amplifier), where BOAs are often highly polarization-dependent with PDG up to 10 dB or more and only amplify one input polarization. On the other hand, SOAs are often labeled as polarization-insensitive, because they do not exhibit such a strong polarization dependence and all polarizations are amplified. However, there remains an inherent difference between the TE and TM modes in the SOA waveguide due to waveguide geometry, confinement factors and strain, and a small residual polarization dependence still remains. Often, vendors do not show measured PDG data publicly or if they do, it is usually more than 0.5 dB. We list here in table 1 some examples of SOAs marketed as polarization-insensitive, where data or specifications on PDG were provided, and also include two screenshots. We were otherwise not able to find commercially available SOAs where PDG below 0.5 dB is explicitly mentioned in the specifications. We have seen, however, that PDG as low as 0.2 dB has been reported many years ago in SOA research [5], which we had previously only listed in the comparison table in the supplementary, but have now explicitly referenced also in the introduction in the main manuscript.

Table 1: A selection of commercially available SOAs marketed as polarization-insensitive, where PDG values were explicitly mentioned in the specifications.

Vendor	Product	PDG according to specs. [dB]
Thorlabs	S7FC1013S, SOA1117S, SOA1117P	1.0
Inphenix	SOAs from 1300 – 1600 nm	1.0 – 1.5
BOX Optronics Tech	1550nm 8dBm SM SOA	0.5 – 1.0
Idealphotonics	MP-SOA-1550-13db-34-SA-ns (C-band)	1.0
Idealphotonics	MP-SOA-1610-15db-20-PA (L-band)	1.5
Agiltron	SOAB-1511211T5N1 (C-band)	1.0 – 2.5

https://www.thorlabs.com/c-band-optical-amplifiers-boas-and-soas-1550-nm
https://inphenix.com/products/semiconductor-optical-amplifier/
https://www.box-laser.com/1550nm-8dbm-sm-soa-semiconductor-optical-amplifier.html
https://www.idealphotonics.com/product/list/772.html
https://photonwares.com/product/semiconductor-optical-amplifier-soa-c-band/

Description

Thorlabs' SOA1013S is a polarization-insensitive optical amplifier with advanced epitaxial wafer growth and opto-electronic packaging techniques that enable a high output saturation power, low noise figure, and large gain across a broad spectral bandwidth.

Specifications

T_{CHIP} = 25 °C, T_{CASE} = 0 - 70 °C

SOA1013S				
	**Symbol**	**Min**	**Typical**	**Max**
**Operating Current**	I_{OP}	-	500 mA	750 mA
**Operating Wavelength Range (C-Band)**	λ	1528 nm	-	1562 nm
**Center Wavelength**	λ_C		1500 nm	
**Saturation Output Power @ -3 dB**	P_{SAT}	12 dBm	14 dBm	-
**Small Signal Gain over C-Band λ @ P_{IN} = -20 dBm**	G	10 dB	13 dB	-
**Gain Flatness over C-Band λ @ P_{IN} = -20 dBm**	ΔG	-	5 dB	7 dB
**Gain Ripple (p-p) @ I_{op}, λ_C**	δG	-	0.1 dB	0.5 dB
**Polarization Dependent Gain**	PDG	-	1.0 dB	1.8 dB
**Noise Figure**	NF	-	8 dB	9.5 dB
**Forward Voltage**	V_F	-	1.6 V	1.8 V
**TEC Operation (Typical / Max @ T_{CASE} = 25 °C / 70 °C)**				
**-TEC Current**	I_{TEC}	-	0.23 A	1.5 A
**-TEC Voltage**	V_{TEC}	-	0.5 V	4.0 V
**-Thermistor Resistance**	R_{TH}	-	10 kΩ	-

Semiconductor Optical Amplifier (SOA) C-Band

1550nm, SM, PM, 14dBm

DATASHEET

Return to the Webpage

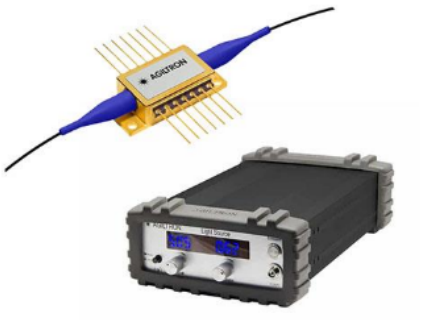

Features

- C-Band Optical Amplification
- Inline Optical Signal Boost

Applications

- Instrument
- Sensor
- Communication

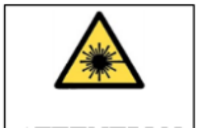

The SOAB is a high-saturation-output-power, high-bandwidth, low-noise booster optical amplifier. It features a highly efficient InP/InGaAsP Quantum Well (QW) layer structure and a reliable ridge waveguide design, ensuring robust performance. The amplifier is housed in a standard 14-pin butterfly package, complete with an integrated thermoelectric cooler (TEC) and thermistor for precise thermal management. The SOAB is polarization-insensitive, amplifying all polarization states, making it an excellent choice for in-line amplification. It is available with either single-mode (SM) or polarization-maintaining (PM) fiber pigtails, both terminated with FC/APC connectors. For PM-fiber-pigtailed models, the connector key is aligned to the slow axis. Options include configurations with no isolators or polarization-insensitive isolators at the input, output, or both, to eliminate reflection induced noise. The SOAB provides a versatile and reliable solution for optical amplification needs.

The basic working principle of an SOAB is the same as a semiconductor laser but without feedback. SOABs amplify incident light through stimulated emission. When the light traveling through the active region, it causes these electrons to lose energy in the form of photons and get back to the ground state. Those stimulated photons have the same wavelength as the optical signal, thus amplifying the optical signal.

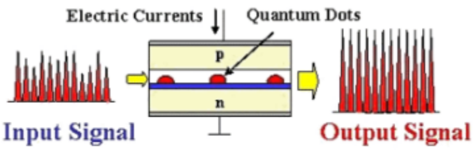

Specifications

Parameter	Min	Typical	Max	Unit
Operating Current		450	500	mA
Operating Wavelength Range (C-Band)	1525	1550	1565	nm
Peak Gain Wavelength		1545		nm
Saturation Output Power @ -3 dB	10	13	14	dBm
Small Signal Gain across λ @ P_{IN} = -20 dBm	15	20		dB
Gain Ripple (p-p) @ I_{op}		0.2	0.8	dB
Polarization Dependent Gain		1	2.5	dB
Noise Figure		6	10	dB
Forward Voltage		1.8	2.0	V
TEC Operation		25	70	°C
TEC Current		0.2	1.5	A
TEC Voltage		0.4	4.0	V
Thermistor Resistance		10		kΩ

6. The authors state that for integrated amplifiers the saturation power is limited to few mW. This is incorrect, in ref. 48 the output power is 145 mW. Also, in the paper "Broadband Packaged Erbium-Doped Polycrystalline Al2O3 Waveguide Amplifier with 24 dB External Net Gain") the output power was 17 dBm. Please correct this.

The reviewer highlights two remarkable results in the integrated rare earth-based amplifier technology. To our knowledge, the 145 mW output power reported in [6] was the first time the 100 mW-level has been surpassed in a truly integrated photonic platform. The 17 dBm (54 mW) output power reported in [7] is a very notable result, as this is a packaged device with high internal and external net gain. However, we believe there might be a slight confusion with the terminology. We have found in literature sometimes the term "saturated *output* power", which is often used to refer to the maximum output power the amplifier can deliver. In other publications, the term "*output* saturation power" is used, which refers to the amplified signal output power level at which the net gain has decreased by 3 dB from its small-signal value due to gain compression with a higher-power signal. This definition with the 3-dB-point is also the one we used in the manuscript. We have now added this as a sentence to the main manuscript to carefully differentiate between "saturated output power" (maximum output power achieved with the amplifier) and the "saturation power" (signal power at which the gain has compressed by 3 dB).

We would still like to clarify, that even though the maximum output power of the listed amplifier devices clearly far exceeds the few mW, their *output* saturation power (3-dB point) is still quite low – in most cases below 10 mW. To the best of our knowledge, more than 10 mW on integrated rare earth-based platforms has only been demonstrated in ref. 48, as also mentioned by the reviewer. We would like to explain in a bit more details how we extracted these numbers, since they were not explicitly mentioned in the publications.

In the manuscript, we specify the *output* saturation power $P_{sat,out}$, which we have seen as a prominent figure of merit in semiconductor optical amplifiers, defined as the amplified signal *output* power at which the gain has reduced by 3 dB. This property is directly related to the *input* saturation power via the gain: $P_{sat,out} = G P_{sat,in}$ [8]. We have noticed that the maximum output power of an amplifier can be significantly higher than the saturation power. For example, in the main manuscript we highlight the 115-mW output saturation power of our LMA device, however, the total output power achieved was more than 500 mW and even up to 800 mW. Although the exact 3-dB-saturation powers are often not explicitly reported in the integrated amplifier literature, it is usually possible to get a good estimate from the provided gain data. With this, we were able to get comparable numbers, which we have listed in the overview of different amplifier technologies in supplementary section 7. In the two examples mentioned by the reviewer, the saturation powers were not explicitly reported, only the maximum output power. Therefore, we performed the following estimation from the gain data provided in the publications as shown in Fig. 7. First, we identified the small-signal gain value at low input power. Then we found the point where the gain has dropped by 3 dB with the corresponding signal input power. This signal power level is the *input* saturation power, which can be transformed into the *output* saturation power by multiplying with the gain at that power level. Following this procedure, we estimate the *input* saturation power to be -13 dBm (50 µW) in [6] and -15 dBm (31 µW) in [7]. The corresponding *output* saturation powers are 8 dBm (6 mW) and 15 dBm (31 mW), respectively. Although, for the latter estimation, the provided gain data at low signal power appears somewhat unreliable due to the presence of parasitic lasing. This tends to quench the gain for the actual signal because some of the gain is now used for the parasitic lasing mode, which we have seen also during our own experiments. In an optimal case without parasitic lasing, the small-signal gain would then be higher, which would shift the 3-dB saturation point to lower power levels. Therefore, the estimation of 31 mW in this case provides rather an upper limit and the actual saturation power is likely lower. For comparison, the *output* saturation power of our LMA amplifier was as high as 115 mW (increasing linearly with pump power), while the *input* saturation power was measured around 250 µW (independent of pump power).

We agree that, with the recently reported saturation powers, the more actual state-of-the-art saturation power level on-chip is more in the few tens of mW, so we have changed the wording in introduction of the main manuscript to reflect that.

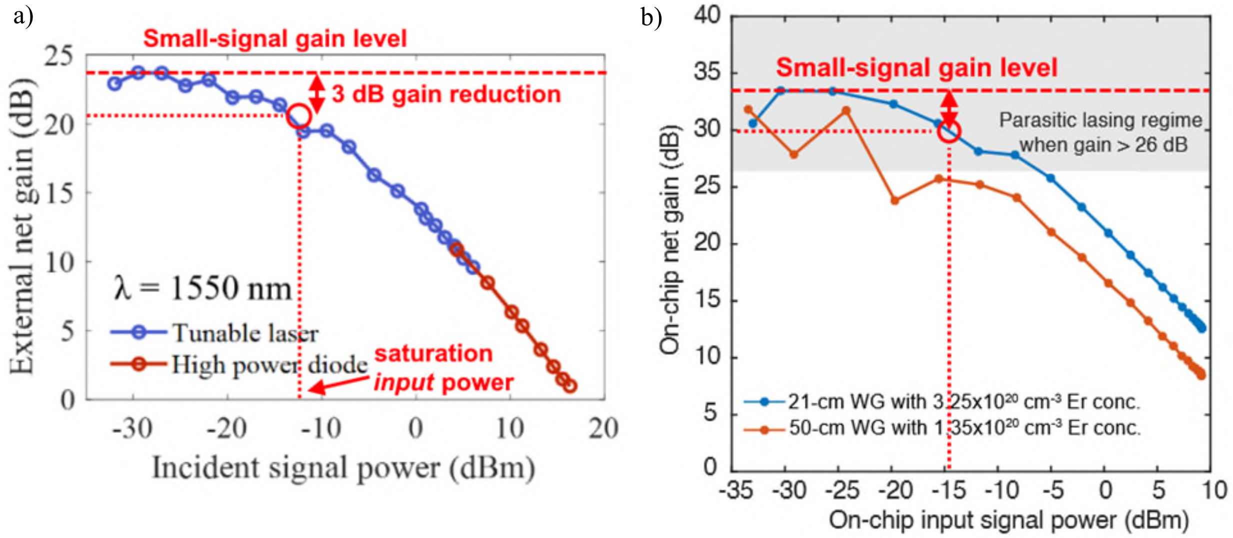

Fig. 7. Two examples for the estimation of the saturation input power from gain data provided in recently published reports. a) is taken from Fig. 2d from [7]. b) is taken from Fig. S9c from the supplementary material of [6]. Annotated as the dashed line is the small-signal gain level at low signal input power. The circle marks the point where the gain has dropped by 3 dB from the small-signal value due to the increased signal input power.

References:

1. H. Fukuda, K. Yamada, T. Tsuchizawa, T. Watanabe, H. Shinojima, and S. Itabashi, "Ultrasmall polarization splitter based on silicon wire waveguides," Opt. Express **14**, 12401 (2006).
2. G. Brunetti, R. Heuvink, E. Schreuder, M. N. Armenise, and C. Ciminelli, "Silicon Nitride Spot Size Converter With Very Low-Loss Over the C-Band," IEEE Photonics Technol. Lett. **35**, 1215–1218 (2023).
3. D. M. Baney, P. Gallion, and R. S. Tucker, "Theory and Measurement Techniques for the Noise Figure of Optical Amplifiers," Opt. Fiber Technol. **6**, 122–154 (2000).
4. C. Sun, M. T. Wade, Y. Lee, J. S. Orcutt, L. Alloatti, M. S. Georgas, A. S. Waterman, J. M. Shainline, R. R. Avizienis, S. Lin, B. R. Moss, R. Kumar, F. Pavanello, A. H. Atabaki, H. M. Cook, A. J. Ou, J. C. Leu, Y.-H. Chen, K. Asanović, R. J. Ram, M. A. Popović, and V. M. Stojanović, "Single-chip microprocessor that communicates directly using light," Nature **528**, 534–538 (2015).
5. K. Morito, M. Ekawa, T. Watanabe, and Y. Kotaki, "High-output-power polarization-insensitive semiconductor optical amplifier," J. Light. Technol. **21**, 176–181 (2003).
6. Y. Liu, Z. Qiu, X. Ji, A. Lukashchuk, J. He, J. Riemensberger, M. Hafermann, R. N. Wang, J. Liu, C. Ronning, and T. J. Kippenberg, "A photonic integrated circuit–based erbium-doped amplifier," Science **376**, 1309–1313 (2022).
7. C. E. Osornio-Martinez, D. B. Bonneville, M. Dijkstra, A. R. Do Nascimento Jr., and S. M. García-Blanco, "Broadband Packaged Erbium-Doped Polycrystalline Al_2O_3 Waveguide Amplifier with 24 dB External Net Gain," Opt. Express **33**, 28985 (2025).
8. E. Desurvire and M. N. Zervas, "*Erbium-Doped Fiber Amplifiers: Principles and Applications*," Phys. Today **48**, 56–58 (1995).

REVIEWER COMMENTS

We would like to thank the reviewers again for taking their time to evaluate our work and give valuable comments. Please find our response to the comments. In the revised manuscript the texts are colored to highlight the changes.

Reviewer #1 (Remarks to the Author):

Thanks for the authors' response and revisions. I have only a few minor points we'd like to see addressed before the paper is ready for final acceptance.

1. It would help readers to understand, early in the paper, why you can't simply take an amplifier that was designed for one regime (pre-amp or power amp) and expect it to perform well in the other. The introduction or results section feels like the right place for this, and it would give the LMA architecture a clearer motivation. Right now, a reader unfamiliar with the tradeoffs might not fully appreciate what problem is being solved.

Thank you for pointing this out. We have added a short overview of the two common approaches for pre-amps and power amps in the final paragraph of the introduction, highlighting how the two amplifier operating modes are typically realized with separate devices due to different performance goals.

2. The 30 dB gain result needs a bit more framing in both the abstract and the main text. As it stands, it could be read as a deployment-ready figure, when in fact it relies on index-matching fluid to suppress facet reflections. That's a perfectly reasonable proof-of-concept approach, but it should be stated plainly. It would also be worth noting that anti-reflection coatings are the practical path forward and are expected to recover comparable performance in a more realistic configuration.

We have added sentences to the abstract and the beginning of section 3.1 of the main text to highlight this (marked in red).

3. The noise figure discussion focuses on the on-chip value of 3.6 dB, but many readers will naturally think in terms of fiber-to-fiber performance. Given that you're looking at roughly 2.2 dB of coupling loss per facet, it's worth walking through what that means for the fiber-to-fiber NF explicitly, and then making the case that improved couplers could bring the fiber-to-fiber figure below 4.0 dB.

Thank you for this suggestion. We have added corresponding fiber-to-fiber performance values to the text in the main manuscript, section 3.2. (marked in red). Especially for the noise figure considerations, we have now outlined the measurement method to obtain the on-chip noise figure and how this corresponds to the fiber-to-fiber situation by including the coupling loss. We now mention both values specifically (3.6 dB on-chip and 5.8 dB fiber-to-fiber) to avoid any confusion. We have also added a short paragraph at the end of that section highlighting the difference between on-chip and off-chip noise figure values due to coupling losses, and that the fiber-to-fiber noise figure may be brought to below 4 dB with improved couplers.

4. Given that this work builds on the authors' prior publication on the same platform, a brief statement clarifying what is actually new here would be useful.

Thank you for your comment. Connected also to the discussion of pre-amplifiers and power amplifiers, we have now added the distinction that our previous amplifier designs and demonstrations targeted only the power amplification regime by focusing on high concentration and a short amplifier length. In this work, we reduced the doping concentration slightly (from ~ 6.0e20/cm3 to 4.0e20/cm^3) and increased

the amplifier length (from 6 cm to 10 cm) to achieve high performance in both operating regimes with the same device.

In the introduction we have paragraphs clearly stating that and the novelty over previous works - “In recent demonstrations [51, 52], such LMA-based devices were optimized for the power amplification regime, designed to amplify seed signals of several milliwatts to well over 1 watt of output power on-chip. In these cases, the amplifier length was kept short, the doping concentration was very high, and the maximum net gain was limited to approximately 16 dB constrained by parasitic lasing due to reflections from the waveguide facets.

In this work, we demonstrate LMA-based silicon photonic amplifiers capable of both high small-signal net gain**,** reaching up to 30 dB**,** and high output saturation power of 115 mW**,** enabling both high-gain amplification and power amplification within a single device. The amplifier length and concentration were optimized to achieve high performance in both amplifier regimes. Typically, in fiber amplifier technology, high-gain amplification and high-power amplification are achieved with separate devices and different amplifier designs. For example, in telecommunication links, pre-amplifiers target high-gain and low-noise amplification of very weak signals in the µW-range just before the receiver. These amplifiers focus on small mode areas to increase optical intensities for more intense gain extraction and longer gain sections to accumulate gain [54]. Power booster amplifiers, on the other hand, aim to amplify already relatively high-power signals from the transmitter in the few-mW-range and boost them to several 100 mW or even Watt-level output powers. Such amplifiers employ shorter gain sections and large mode area designs (e.g. LMA or double-clad fiber) to improve gain saturation limitations and reduce signal intensities to avoid non-linear effects. In addition, compared to previous reports [51, 52] we demonstrate an ultra-low on-chip noise figure of approximately 3.6 dB at 30 dB gain and importantly present a novel method for achieving tunable polarization-dependent gain (PDG) in integrated photonics, with the ability to reduce PDG to 0 dB**.** This level of polarization insensitivity surpasses even that of state-of-the-art single-mode fibers, which still exhibit residual PDG due to fabrication imperfections. We demonstrate complete PDG suppression across a wide range of input signal powers, from a few microwatts to several milliwatts, by adjusting either the pump power or the pump polarization state launched into the amplifier. In the following, section 2 describes the device design and section 3 contains the results on high net gain, noise figure and the concept and results on PDG, which is followed by discussion and conclusion.”

I have carefully reviewed the revised manuscript, the authors' comprehensive response letter, and the additional data provided. While I appreciate the authors' detailed rebuttals and the effort to address previous concerns, I remain unconvinced that this manuscript presents the breakthrough advances required for publication in Nature Communications. The authors have provided extensive technical arguments defending their work, but these responses inadvertently highlight that this work represents incremental characterization and optimization of a recently published platform rather than transformative science. The following are the detailed reasons:

1. The authors dedicate substantial effort in their rebuttal to defending the novelty of their work, yet their arguments actually underscore the incremental nature of this contribution. The comparison with Rönn et al. (Nature Communications 2019) is particularly revealing. The authors point out that Rönn achieved only 1.98 dB actual gain in a 1.2 mm device despite reporting a 20 dB/cm gain coefficient, and they emphasize that "total device net gain is the meaningful performance metric for any practical application, rather than the gain per centimeter." This argument is entirely reasonable. However, the authors then acknowledge in their response that "large-mode-area (LMA) and high-power operation have already been demonstrated in our earlier work," referring to their Nature Photonics 2025 paper, where "more than one watt of output power was achieved in the power-amplification regime." They state that what remained unclear was "whether an LMA device can also provide high amplification for weak input signals." This reveals the fundamental problem: the authors are essentially asking whether measuring small-signal gain in addition to large-signal gain in the same device architecture constitutes sufficient novelty for Nature Communications. In fact, demonstrating that a power amplifier can also function as a pre-amplifier when operated at different signal levels is an expected capability, not a breakthrough discovery. The detailed simulations provided in their rebuttal (Figure 1 showing gain saturation with length, comparisons with erbium-doped fiber amplifiers) explain amplifier physics, but these are well-established principles from the fiber amplifier literature that the authors are applying to their integrated platform. The authors themselves cite textbooks (Becker et al., 1999) showing that these gain saturation behaviors have been understood for decades.
2. The achievement of 30 dB gain is presented as a major contribution, but examination of how this was accomplished raises serious concerns about both scientific novelty and practical viability. In their response, the authors explicitly state that achieving gains above 16 dB required applying index-matching fluid or glue (Norland NOA 148) to suppress parasitic lasing from facet reflections. While this demonstrates good experimental technique, it fundamentally undermines claims of an integrated photonic breakthrough. More concerning is what the authors reveal about the experimental difficulties this approach created. They state multiple times that "during further high-power testing with different index-matching fluids the input facet of this waveguide got damaged, most likely due to deterioration of one of the tested fluids which was not able to withstand the

prolonged high-power exposure." This occurred specifically with their best-performing 10.7 cm device. Later they note they "could not confirm the small-signal 0-dB-point directly by measurement, because we avoided pump powers > 0.9 W since the index-matching fluid on the waveguide facets began to degrade from the prolonged high-power pumping." This fragility is not a minor implementation detail, but it prevented them from completing their characterization and destroyed their best device. They cannot claim to advance integrated photonics while relying on manually applied fluids that degrade under operating conditions and prevent full device characterization.

3. The authors report a noise figure of 3.6 dB at 30 dB gain and present extensive arguments in their rebuttal defending this as a key contribution. However, their own analysis reveals this is fundamentally expected performance rather than breakthrough science. They calculate the theoretical quantum-limited noise figure as 3.18 dB for their in-band pumped configuration, meaning they achieved performance only 0.42 dB above the fundamental limit. They correctly explain the physical mechanism: "the low spectral overlap of the pump and emission bands" in thulium results in "large differences between σ_{em} and σ_{abs} at the pump wavelength, which enables a high population inversion" leading to low spontaneous emission factor and thus low noise. They cite Romano et al. (Opt. Express 2018) demonstrating 3.1 dB noise figure in thulium-doped fiber amplifiers using the same pumping approach. The authors provide a comparison table (Table 3 in their rebuttal) showing noise figures across various amplifier technologies. This table actually undermines their novelty claim. Commercial EDFAs achieve 4.5 dB noise figures, and the 3.1 dB demonstrated by Romano in fiber TDFAs already approached the quantum limit seven years ago. They have not discovered new physics, broken fundamental limits, or enabled qualitatively new capabilities. Furthermore, the authors' own data show the noise figure increases substantially (approaching 5 dB) at higher signal powers due to gain saturation effects, as expected from well-understood amplifier physics. The authors provide no evidence that integrated implementation provides advantages over fiber solutions for low-noise applications.
4. The PDG tuning capability is presented as the most novel contribution, and the authors' rebuttal includes the most vigorous defense of this aspect. They state that achieving "a level of polarization-dependent gain (PDG) that has not been demonstrated even in fiber amplifiers, let alone in integrated photonic or semiconductor optical amplifiers" represents "a major breakthrough in device development of practical importance." They argue that if PDG were easily addressable, "the industry would not have invested heavily in the development of symmetric fiber designs specifically to minimize PDG." However, examination of their experimental demonstration reveals significant limitations that contradict claims of a practical breakthrough. First, the PDG tuning requires continuous active control that is not currently implementable in an integrated fashion. The authors demonstrate two tuning approaches: adjusting pump power or adjusting pump polarization. For pump power tuning, their data (Figure 4b) shows every

change in signal power necessitates corresponding pump power adjustment to maintain zero PDG—a requirement for continuous active feedback control that adds substantial system complexity and is antithetical to the passive, stable operation preferred in telecommunications. For pump polarization tuning, the authors acknowledge the fundamental implementation challenge. They propose integrating two orthogonally polarized pump diodes in a bidirectional scheme, but this remains entirely conceptual. Second, they present complex polarization diversity architectures from SOA literature as evidence that PDG is difficult to address, implying their approach is simpler. However, their approach requires bidirectional pumping with orthogonal polarization control and active feedback—comparable complexity to the polarization diversity schemes they criticize.

5. The timeline and overlap with the authors' recent publications remain deeply problematic despite their attempts to differentiate the work. The Nature Photonics paper (Ref. 50, published January 2025) demonstrated "Watt-class silicon photonics-based optical high-power amplifier" using the same LMA waveguide architecture. The Light Science & Applications paper (Ref. 51, also January 2025) demonstrated "Sub-2W tunable laser based on silicon photonics power amplifier" using the same platform. Both papers are authored by the same research group, use the same silicon nitride/aluminum oxide platform, employ the same RF sputtering deposition techniques, and operate on the same physical principles. The authors argue in their rebuttal that the novelty lies in demonstrating high output power, a high small-signal net gain of 30 dB, an exceptionally low noise figure of 3.6 dB, and, most notably, the demonstration of polarization-dependent gain (PDG) that can be tuned to absolute zero all within the same device. However, Nature Communications explicitly discourages fragmenting research into multiple papers. Simply adding characterization metrics (noise figure, PDG) to a recently published platform is not enough.
